# Long-term Clinical and Cost-effectiveness of Early Endovenous Ablation in Venous Ulceration: A Randomized Clinical Trial

**DOI:** 10.1001/jamasurg.2020.3845

**Published:** 2020-09-23

**Authors:** Manjit S. Gohel, Jocelyn Mora, MSc, Matyas Szigeti, David M. Epstein, Francine Heatley, Andrew Bradbury, Richard Bulbulia, Nicky Cullum, Isaac Nyamekye, Keith R. Poskitt, Sophie Renton, Jane Warwick, Alun H. Davies

**Affiliations:** 1 Cambridge University Hospitals National Health Service Foundation Trust, Cambridge, United Kingdom; 2 Department of Surgery and Cancer, Imperial College London, London, United Kingdom; 3 Imperial Clinical Trials Unit, School of Public Health, Imperial College London, London, United Kingdom; 4 Department of Applied Economics, University of Granada, Granada, Spain; 5 Institute of Cardiovascular Sciences, University of Birmingham, Birmingham, United Kingdom; 6 Gloucestershire Hospitals National Health Service Foundation Trust, Cheltenham, United Kingdom; 7 Medical Research Council Population Health Research Unit, Nuffield Department of Population Health, University of Oxford, Oxford, United Kingdom; 8 Clinical Trial Service Unit and Epidemiological Studies Unit, Nuffield Department of Population Health, University of Oxford, Oxford, United Kingdom; 9 University of Manchester & Manchester University National Health Service Foundation Trust, Manchester, United Kingdom; 10 Worcestershire Acute Hospitals National Health Service Trust, Worcestershire, United Kingdom; 11 North West London Hospitals National Health Service Trust, London, United Kingdom; 12 Warwick Clinical Trials Unit, University of Warwick, Warwick, United Kingdom

## Abstract

**Importance:**

One-year outcomes from the Early Venous Reflux Ablation (EVRA) randomized trial showed accelerated venous leg ulcer healing and greater ulcer-free time for participants who are treated with early endovenous ablation of lower extremity superficial reflux.

**Objective:**

To evaluate the clinical and cost-effectiveness of early endovenous ablation of superficial venous reflux in patients with venous leg ulceration.

**Design, Setting, and Participants:**

Between October 24, 2013, and September 27, 2016, the EVRA randomized clinical trial enrolled 450 participants (450 legs) with venous leg ulceration of less than 6 months’ duration and superficial venous reflux. Initially, 6555 patients were assessed for eligibility, and 6105 were excluded for reasons including ulcer duration greater than 6 months, healed ulcer by the time of randomization, deep venous occlusive disease, and insufficient superficial venous reflux to warrant ablation therapy, among others. A total of 426 of 450 participants (94.7%) from the vascular surgery departments of 20 hospitals in the United Kingdom were included in the analysis for ulcer recurrence. Surgeons, participants, and follow-up assessors were not blinded to the treatment group. Data were analyzed from August 11 to November 4, 2019.

**Interventions:**

Patients were randomly assigned to receive compression therapy with early endovenous ablation within 2 weeks of randomization (early intervention, n = 224) or compression with deferred endovenous treatment of superficial venous reflux (deferred intervention, n = 226). Endovenous modality and strategy were left to the preference of the treating clinical team.

**Main Outcomes and Measures:**

The primary outcome for the extended phase was time to first ulcer recurrence. Secondary outcomes included ulcer recurrence rate and cost-effectiveness.

**Results:**

The early-intervention group consisted of 224 participants (mean [SD] age, 67.0 [15.5] years; 127 men [56.7%]; 206 White participants [92%]). The deferred-intervention group consisted of 226 participants (mean [SD] age, 68.9 [14.0] years; 120 men [53.1%]; 208 White participants [92%]). Of the 426 participants whose leg ulcer had healed, 121 (28.4%) experienced at least 1 recurrence during follow-up. There was no clear difference in time to first ulcer recurrence between the 2 groups (hazard ratio, 0.82; 95% CI, 0.57-1.17; *P* = .28). Ulcers recurred at a lower rate of 0.11 per person-year in the early-intervention group compared with 0.16 per person-year in the deferred-intervention group (incidence rate ratio, 0.658; 95% CI, 0.480-0.898; *P* = .003). Time to ulcer healing was shorter in the early-intervention group for primary ulcers (hazard ratio, 1.36; 95% CI, 1.12-1.64; *P* = .002). At 3 years, early intervention was 91.6% likely to be cost-effective at a willingness to pay of £20 000 ($26 283) per quality-adjusted life year and 90.8% likely at a threshold of £35 000 ($45 995) per quality-adjusted life year.

**Conclusions and Relevance:**

Early endovenous ablation of superficial venous reflux was highly likely to be cost-effective over a 3-year horizon compared with deferred intervention. Early intervention accelerated the healing of venous leg ulcers and reduced the overall incidence of ulcer recurrence.

**Trial Registration:**

ClinicalTrials.gov identifier: ISRCTN02335796

## Key Points

**Question:**

In patients with venous leg ulceration and superficial reflux, what is the clinical and cost-effectiveness of early endovenous ablation of reflux?

**Findings:**

In this 450-patient, multicenter, randomized clinical trial, early endovenous ablation with compression accelerated venous ulcer healing, reduced the overall incidence of ulcer recurrence, and was highly cost-effective compared with compression with deferred intervention.

**Meaning:**

To deliver clinical and cost benefits, leg ulcer care pathways should be revised to include early assessment and treatment of superficial venous reflux.

## Teaser

This randomized clinical trial uses data from the Early Venous Reflux Ablation trial to evaluate the long-term clinical and cost benefits of combined early endovenous ablation and compression compared with compression therapy alone and deferred ablation for treating superficial venous reflux of the leg.

## Introduction

Venous leg ulceration is the most extreme manifestation of chronic venous disease, and worldwide prevalence is increasing.^1,2^ Compression therapy has been shown to improve ulcer healing, and 1-year outcomes from the Early Venous Reflux Ablation (EVRA) trial revealed that early endovenous ablation of superficial venous reflux (varicose veins) accelerated healing of venous leg ulcers compared with deferred intervention.^3,4^ Early intervention was also shown to be cost-effective in the short term.^5^ In the Effect of Surgery and Compression on Healing and Recurrence (ESCHAR) study, superficial venous surgery reduced venous ulcer recurrence at 4 years from 56% in participants treated with compression alone to 31% in the group treated with compression and varicose vein surgery.^6^ Ulcer recurrence rates are likely to be higher than the 56% in the ESCHAR trial because compression is often not prescribed and compliance is poor, particularly outside clinical trials. Superficial venous surgery has largely been superseded by endovenous ablation procedures (ultrasound-guided foam sclerotherapy and thermal and nonthermal ablation), but long-term outcomes in patients with venous leg ulcers are unknown.

Extended follow-up was performed for participants in the EVRA trial to evaluate the influence of early endovenous ablation of superficial venous reflux on outcomes up to 5 years for participants with venous leg ulcers.

## Methods

### Study Design and Population

This parallel-group randomized controlled trial was conducted in 20 centers in the United Kingdom (trial protocol in Supplement 1 and eTable 1 in Supplement 2), where potential participants were screened from October 24, 2013, to September 27, 2016. Eligible participants had venous leg ulceration that had been present for 6 weeks to 6 months in addition to significant superficial venous reflux as assessed by the treating clinician. All trial centers had established leg ulcer referral and treatment pathways and were able to provide endovenous intervention within 2 weeks.

The study design and 1-year outcomes of the EVRA trial have been published previously.^3,4^ Extended follow-up was approved by the South West-Central Bristol Research Ethics Committee on May 24, 2017. The independent trial steering committee and independent data and safety monitoring committees were retained to provide ongoing oversight for the study extension. All patients provided written informed consent for long-term follow-up at randomization, and this consent was reaffirmed for all participants contacted for long-term data collection. The EVRA trial was funded by the UK National Institute for Health Research Health Technology Assessment Programme, and the funder of the study had no role in design and conduct of the study; collection, management, analysis, and interpretation of the data; preparation, review, or approval of the manuscript; or the decision to submit the manuscript for publication. During protocol development, a patient focus group was used to guide study design, and a patient was also included as a member of the trial steering committee. This study followed the Consolidated Standards of Reporting Trials (CONSORT) reporting guideline.

### Randomization

Participants were assigned randomly in a 1:1 ratio to receive compression therapy and endovenous ablation within 2 weeks (early-intervention group) or to receive compression therapy alone with deferred endovenous ablation once the ulcer had healed, or after 6 months if the ulcer had not healed (deferred-intervention group). Randomization sequences were created in advance for each center by a trial statistician, and randomly permuted blocks were used with 2 block sizes. Surgeons, participants, and follow-up assessors were not blinded to the treatment group. Photographic verification for healing of primary ulceration was performed by clinical experts blinded to treatment allocation.

### Procedures

Wound care and compression therapy were guided by local protocols, and multilayer elastic compression (2, 3, or 4 layers), short-stretch bandaging, and compression hosiery were all accepted. The endovenous treatment was left to the discretion of the responsible clinical teams, with endovenous thermal ablation modalities (laser or radiofrequency ablation), ultrasound-guided foam sclerotherapy, or nonthermal nontumescent endovenous interventions performed alone or in combination. Decisions regarding treatment of branch varicosities or perforators were left to physician choice. EVRA trial centers had extensive experience in performing endovenous ablation procedures. Participants in the early-intervention group underwent follow-up duplex ultrasound assessment 6 weeks after endovenous ablation, and additional interventions for superficial venous reflux in either group were performed at the discretion of the treating clinical teams. All participants were advised to use compression hosiery after ulcer healing, guided by local policy; additional duplex ultrasound assessment was not in the study protocol.

Telephone follow-up for all living participants was performed between October 2018 and March 2019 to obtain primary and secondary end point data. Where possible, participants in the EVRA trial were reminded at the 12-month visit to record any recurrent ulcers and health care visits in a participant diary to aid in subsequent recall. In the extended-phase follow-up, participants were asked on the telephone (using a standardized questionnaire) about ulcer recurrences (defined as any wound on the study leg) and asked to recall dates of recurrence, subsequent healing, and details of additional treatments. Hospital and community clinical records were reviewed for further verification, and further calls were made to participants to clarify discrepancies.

A disease-specific quality-of-life assessment (Aberdeen Varicose Vein Questionnaire) and 2 generic quality-of-life assessments (the EuroQol Group 5-Dimension 5-Level questionnaire [EQ-5D-5L] and the Medical Outcomes Study 36-Item Short-Form Health Survey) (eTable 2 in Supplement 2) were performed between October 2018 and March 2019 (either on telephone or by mail). Adverse events were recorded in accordance with Good Clinical Practice guidelines.

### Outcome Assessments

The primary outcome for the extended follow-up phase of the study was time to first ulcer recurrence from date of ulcer healing. The 1-year results, with time to ulcer healing as the primary outcome measure, were reported previously.^3,4^ Healing of the primary venous leg ulcer was defined as complete re-epithelialization of all ulceration on the randomized (reference) leg with no scab or requirement for dressings, and a blinded verification process was used to confirm healing.^7^

The secondary outcome measures were time to first ulcer recurrence from date of randomization, the proportion of participants with recurrent ulceration at different time points (ulcer recurrence rate), time to healing of index and recurrent ulcers, length of time free from ulcers from randomization to final follow-up (ulcer-free time), recurrent ulcer incidence rate and incidence rate ratio, participant-reported health-related quality of life, and cost-effectiveness.

### Statistical Analysis

Statistical analyses of the data were performed from August 11, 2019, to November 4, 2019. The trial was designed to detect a 15% absolute difference in ulcer-healing rates at 24 weeks (assuming a 60% rate of ulcer healing in participants randomized to compression alone) with 90% power and 2-sided alpha level of 5%. Assuming 90% of the participants in the EVRA trial would achieve ulcer healing and 15% losses to follow-up, death, or withdrawal from the study, we estimated that 344 participants would be available for analysis of ulcer recurrence. For extended follow-up analysis, we calculated that this was sufficient to detect a 15% difference in ulcer recurrence (30% in the early-intervention group vs 45% in the deferred-intervention group) with 82% power or a 20% difference in ulcer recurrence (30% in the early-intervention group and 50% in the deferred-intervention group) with 97% power.

The null hypothesis was that there is no difference in time to ulcer recurrence between the early-intervention group and the deferred-intervention group. This was tested using Cox regression with center as a random effect and participant age, ulcer size, and chronicity as fixed effects. We used Cox regression, adjusted as mentioned, to test for differences in time to healing of primary ulcer and recurrent ulcers. Ulcer recurrence rates (unadjusted) were calculated at annual time points up to 4 years with 95% CIs using the Kaplan-Meier method.^8^ Moreover, the incidence rate of recurrent ulcers (ulcers per person-years) and incidence rate ratios with 95% CIs were calculated. Ulcer-free time was defined as the total number of days that the reference leg remained healed during the entire follow-up period. We used a Cox regression model adjusted for center, patient age, ulcer size, and ulcer chronicity, as mentioned, as well as length of follow-up (as a fixed effect) to test the hypothesis that there was no difference in ulcer-free time between the early-intervention and deferred-intervention groups. Participants who did not consent to the extended follow-up are included to 12 months only. Adverse events were recorded.

Differences between study groups to 1 year for each quality-of-life measure have been published previously.^3^ We used 3-level mixed models to assess differences in each quality-of-life measure between the 2 treatment groups. All analyses were performed on intention-to-treat. Participants whose primary ulcer did not heal were not eligible for analysis for ulcer recurrence, but were included in all other secondary analyses. There were no statistical adjustments for multiple testing. We performed per-protocol analyses for time to ulcer healing and time to first ulcer recurrence, and statistical significance was set at 5%.

### Health Economic Analysis

We performed an in-trial health economic evaluation and estimated costs and quality-adjusted life years (QALYs) from the perspective of the UK National Health Service and Personal Social Services over a 3-year time horizon. Results to 1 year have been published previously.^5^ The price year was the 2017 to 2018 period. Discounting was applied according to UK Government guidelines (3.5% per year for costs and health outcomes).^9^ Study conduct and reporting complied with current guidelines for economic evaluation.^10^ We collected details of resource use in hospital and community care related to venous leg ulcer treatment, adverse events, or complications of venous leg ulcers or treatments. We used case note review and questionnaires completed at baseline and monthly thereafter to 1 year, plus 1 further telephone follow-up between October 2018 and March 2019, with notes review for additional verification. Each item of resource use was multiplied by unit costs obtained from published literature,^11^ national unit costs,^12,13^ and manufacturers’ list prices to calculate overall costs for each participant (eTable 3 in Supplement 2).

The EQ-5D-5L was completed at baseline, 6 weeks, 6 months, 12 months, and 1 further follow-up between October 2018 and March 2019. Utility indices for each individual at each follow-up time were calculated from the EQ-5D-5L questionnaire using the tariff recommended by the National Institute for Health and Care Excellence.^14^ Cost and EQ-5D-5L data were analyzed using mixed models and total mean costs, and total mean QALYs were estimated for the 3-year time horizon. Sensitivity analyses used an alternative tariff for the EQ-5D-5L, per-protocol analysis and 4- and 5-year time horizons.^15^ Uncertainty in mean costs and QALYs was quantified using bootstrapping and presented using cost-effectiveness acceptability curves (full description in eMethods and eTable 3 of Supplement 2).

## Results

### Patients

From October 24, 2013, through September 27, 2016, we randomly assigned 450 participants to undergo early intervention (224 participants) or deferred intervention (226 participants) in addition to compression therapy. The early-intervention group consisted of 224 participants (mean [SD] age, 67.0 [15.5] years; 127 men [56.7%] and 97 women [43.3%]; 206 White participants [92%]). The deferred-intervention group consisted of 226 participants (mean [SD] age, 68.9 [14.0] years; 120 men [53.1%] and 106 women [46.9%]; 208 White participants [92%]) (Table 1).^3,4^ Of 224 participants randomized to early intervention, 203 (90.6%) underwent endovenous ablation within 2 weeks of randomization. Of 226 participants in the deferred-intervention group, 171 (75.6%) were treated with endovenous ablation within 12 months (Table 1). The final telephone follow-up was completed on March 28, 2019.

**Table 1.** Baseline Characteristics and Details of Interventions Performed

Characteristic	No. (%)^a^	
	Early intervention (n = 224)	Deferred intervention (n = 226)
Age, mean (SD), y	67.0 (15.5)	68.9 (14.0)
Sex		
Women	97 (43.3)	106 (46.9)
Men	127 (56.7)	120 (53.1)
Body mass index, mean (SD)^b^	30.1 (7.8) [n = 218]	30.4 (7.4) [n = 219]
Race/ethnicity		
White	206 (92.0)	208 (92.0)
Other^c^	18 (8.0)	18 (8.0)
History of DVT^d^	15 (6.7)	15 (6.6)
Diabetes	34 (15.2)	28 (12.4)
Previous leg ulceration^d^	118 (52.7)	117 (52.0) [n = 225]
Ulcer chronicity, median (IQR), mo^e^	3.2 (2.3-4.2)	3.0 (1.7-4.2)
Trial leg		
Right	107 (47.8)	115 (50.9)
Left	117 (52.2)	111 (49.1)
Ulcer size,^f^ median (IQR), cm^2^	2.4 (1.0-7.1)	2.9 (1.1-8.2)
Presence of deep reflux^d^^,^^g^	74 (33.0)	69 (30.5)
Pattern of superficial reflux at baseline^d^		
GSV reflux alone	123 (54.9)	125 (55.4)
SSV reflux alone	25 (11.2)	30 (13.3)
GSV and SSV reflux	65 (29.0)	56 (24.8)
Other pattern of reflux	11 (4.9)	15 (6.6)
Timing of first endovenous treatment, from randomization		
Within 2 wk	203 (90.6)	1 (0.4)
Between 2 wk and 12 mo^h^	15 (6.7)	170 (75.2)
After 12 mo	0 (0.0)	8 (3.5)
No treatment^i^	6 (2.7)	47 (20.8)
Total No. of procedures	283	227
No. of procedures per participant		
1	164	144
2	43	23
3	11	11
4	0	1

Abbreviations: DVT, deep vein thrombosis; GSV, great saphenous vein; IQR, interquartile range; SSV, small saphenous vein.

^a^ Values are presented as No. (%) for categorical variables and mean (SD) for continuous variables unless otherwise specified.

^b^ Calculated as weight in kilograms divided by height in meters squared.

^c^ Early-intervention group: Asian, 11; Black, 3; and other, 4; deferred-intervention group: Asian, 12; Black, 5; and other, 1.

^d^ In randomized leg.

^e^ As reported by participant.

^f^ Ulcer size evaluated using digital planimetry from standardized digital photographs by assessor blinded to intervention group.

^g^ Defined as presence of retrograde flow in common femoral, femoral, or popliteal veins of >1-second duration after augmentation.

^h^ Further details of timings of interventions have been published previously.^3,4^

^i^ Reasons for no treatment in the deferred-intervention group were patient choice (16 of 47), patient died (7 of 47), withdrawal from study (7 of 47), lost to follow-up (5 of 47), clinician decision (3 of 47), and reason not recorded (9 of 47).

**Figure 1.**
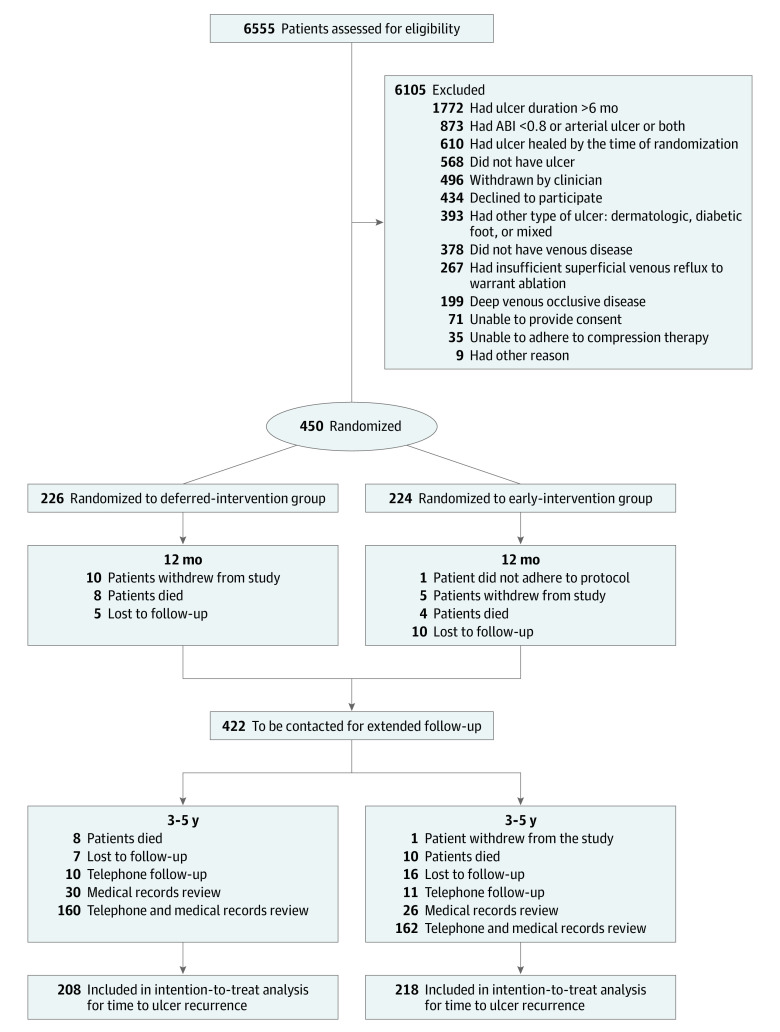
Consort Diagram Showing Enrollment, Allocation, 1-Year, and Extended Follow-Up ABI indicates ankle-brachial index.

Data were collected over the telephone and from medical notes or from medical notes alone for 399 of 422 participants (94.5%) still participating at 1 year (Figure 1). Median follow-up period from randomization was 1286 days (interquartile range [IQR], 1038-1531 days) in the early-intervention group and 1287 days (IQR, 1063-1519 days) in the deferred-intervention group. Mortality was similar between the 2 groups, and no participants died as a result of intervention (eFigure 1 in Supplement 2).

### Ulcer Recurrence

Of the 426 participants whose leg ulcer had healed, 121 (28.4%) experienced at least 1 recurrence. There were 175 episodes of recurrent ulceration during follow-up (72 in the early-intervention group [56 participants]; 103 in the deferred-intervention group [65 participants]).

**Figure 2.**
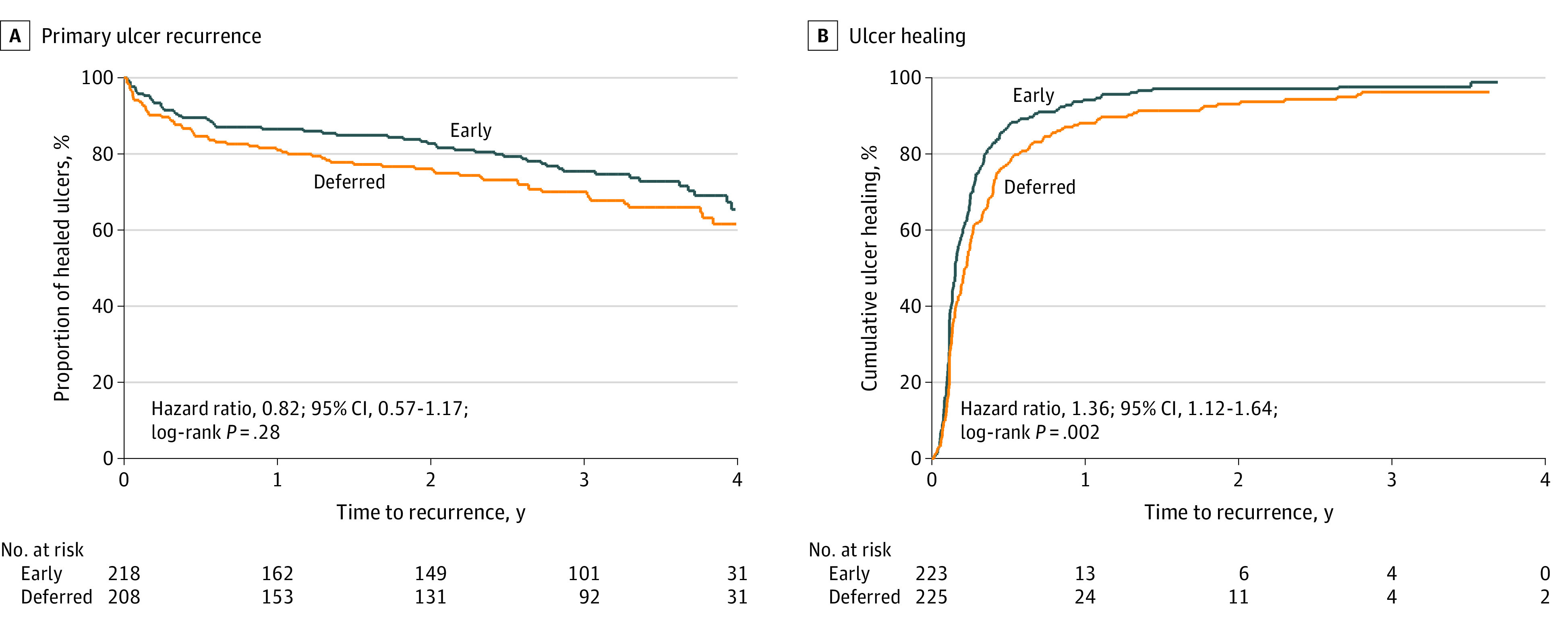
Kaplan-Meier Curves for Time to Primary Ulcer Recurrence and Ulcer Healing in Early-Intervention and Deferred-Intervention Groups

Time to first recurrence from ulcer healing (adjusted for participant age, ulcer size, and ulcer chronicity) was similar in the early-intervention group and the deferred-intervention group (hazard ratio [HR] for ulcer recurrence, 0.82; 95% CI, 0.57-1.17; *P* = .28) (Figure 2A). Calculating time to ulcer recurrence from randomization rather than date of healing did not affect these findings (HR, 0.86; 95% CI, 0.60-1.24; *P* = .43) (eFigure 2 in Supplement 2). Ulcer recurrence rates (from ulcer healing) at 4 years were 34.6% (95% CI, 26.7%-44.0%) for the early-intervention group and 38.4% (95% CI, 30.8%-47.2%) for the deferred-intervention group (Table 2). In the early-intervention group, 72 recurrent ulcers occurred in 675.5 years of follow-up after healing of the primary ulcer compared with 103 ulcers in the deferred-intervention group during 636.0 years of follow-up. Therefore, ulcers recurred at a rate of 0.11 per person-year in the early-intervention group and 0.16 per person-year in the deferred-intervention group (incidence rate ratio, 0.658; 95% CI, 0.480-0.898, *P* = .003).

**Table 2.** Ulcer Recurrence Rates in Early-Intervention and Deferred-Intervention Groups

Study group	Follow-up, y	No.^a^	Recurrences	Cumulative recurrence rate, %	95% CI
Early-intervention group	1	162	28	13.48	9.51-18.94
	2	150	7	17.28	12.71-23.26
	3	102	12	24.56	18.99-31.41
	4	32	8	34.6	26.7-44.04
	5	1	1	NA	NA
Deferred-intervention group	1	154	38	18.98	14.18-25.13
	2	132	9	23.9	18.52-30.53
	3	93	10	29.95	23.92-37.1
	4	32	8	38.42	30.81-47.18
	5	1	0	NA	NA

Abbreviation: NA, not available.

^a^ Number of participants successfully followed up for ulcer recurrence at each time period postrandomization.

### Secondary Outcomes

Time to ulcer healing of the primary ulcer was shorter in the early-intervention group compared with the deferred-intervention group (HR, 1.36; 95% CI, 1.12-1.64, *P* = .002) (Figure 2B). Unlike the 1-year healing outcomes published previously,^3,4^ this analysis also included primary ulcers that healed after 12 months. There was no clear difference in time to healing of recurrent ulcers between the early-intervention group and the deferred-intervention group (HR for healing including all ulcer recurrences, 1.10; 95% CI, 0.79-1.54; *P* = .58; eFigure 3 in Supplement 2) (HR for healing of first recurrence, 0.91; 95% CI, 0.62-1.35; *P* = .64).

The median ulcer-free time was 1137 days (IQR, 860-1411 days) in the early-intervention group and 1090 days (IQR, 625-1364 days) in the deferred-intervention group. Adjusting for follow-up period, participant age, ulcer size, and ulcer chronicity, there was no difference between the groups (HR for greater ulcer-free time, 0.84; 95% CI, 0.69-1.02; *P* = .07). Prespecified per-protocol analyses are presented in eFigures 4 and 5 in Supplement 2. During extended follow-up, the Aberdeen Varicose Vein Questionnaire, EQ-5D-5L, and the 36-Item Short-Form Health Survey domains were similar between the 2 groups (eTable 4 in Supplement 2).

### Health Economic Analysis

**Figure 3.**
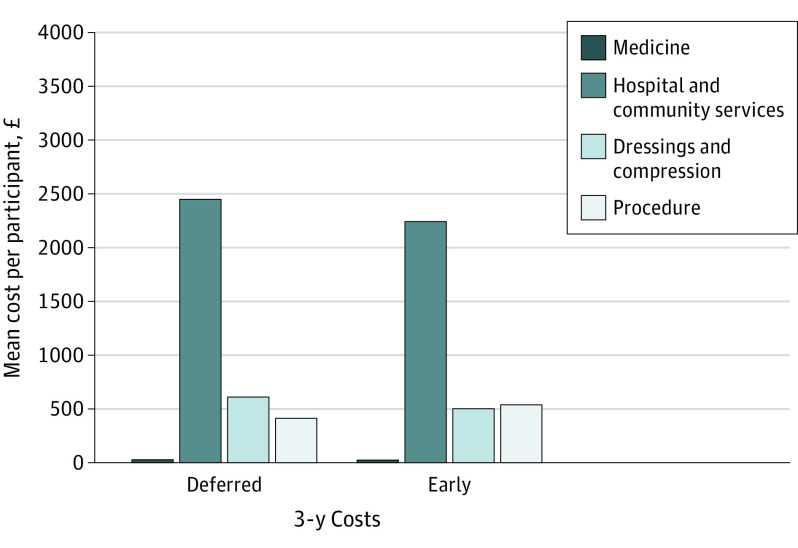
Mean Cost Per Participant at 3 Years

Full details of resource use and costs for the 2 groups are presented in eTables 5 and 6 in Supplement 2. Discounted total mean cost of early intervention was –£155 (95% CI, –£1262 to £953) ($–213 [95% CI, –$1654 to $1249])compared with deferred intervention per participant over 3 years (Figure 3; and eTable 7 in Supplement 2), indicating that early intervention was, on average, the less costly strategy. Participants randomized to early intervention experienced, on average, greater QALYs after 3 years (mean difference in QALY, 0.073; 95% CI, –0.06 to 0.20) using the EQ-5D-5L tariff recommended by National Institute for Health and Care Excellence. Early intervention was therefore a dominant strategy, with lower mean cost and greater mean QALY benefit. Findings were similar for 4-year and 5-year horizons (eTable 7 in Supplement 2) and with a per-protocol analysis (eTable 8 in Supplement 2), although the difference in QALY was smaller at 3 years using an alternative tariff for EQ-5D-5L (eTable 7 in Supplement 2). Analysis using bootstrap simulations demonstrated that early intervention was 91.6% likely to be cost-effective at a willingness-to-pay threshold of £20 000 ($26 283) per QALY and 90.8% at a threshold of £35 000 ($45 995) (eFigures 6 and 7 in Supplement 2).

## Discussion

One-year results from the EVRA trial showed that early ablation of superficial venous reflux accelerated healing of venous leg ulcers.^3,4^ Longer-term follow-up in this study demonstrated that fewer recurrent ulcers per year of follow-up occurred in the early-intervention group, even though the time to first ulcer recurrence did not differ between the groups. The total mean costs were lower in the early-intervention group, and participants reported higher QALYs, indicating that early intervention is highly likely to be cost-effective irrespective of the willingness-to-pay threshold used by the health care system.

Ultrasound-guided foam sclerotherapy was the most common endovenous treatment in this trial, and some studies have reported high rates of technical failure compared with other endovenous modalities or open varicose vein surgery.^11,16^ The 4-year ulcer recurrence rates in this trial are comparable to those of previous studies evaluating ulcer recurrence after open varicose vein surgery, and outcomes in both groups of the EVRA trial are favorable compared with outcomes with compression alone.^6,17^ These findings support the strategy adopted in this study, where the choice of endovenous modality was left to the discretion of the treating clinician. Ablating superficial venous reflux is likely to be more important than the choice of modality.

### Strengths and Limitations

The health economic benefits of early intervention demonstrated in this trial are particularly compelling because the premise of a less costly treatment strategy that offers more QALYs is an important driver for change in behavior irrespective of the country or health care system. The method of follow-up is a limitation of this study, as only telephone follow-up at a single time point after 1 year was possible owing to funding limitations; photographic assessment was not deemed feasible. However, most participants in this trial were kept under regular surveillance by recruiting centers as part of normal clinical care, resulting in accurately recorded outcome data. One-fifth of the participants in the deferred-intervention group did not undergo endovenous intervention at all. It is difficult to predict whether clinical outcomes would have been better if all participants had been treated, but delaying intervention was associated with fewer participants undergoing endovenous ablation. The results of this study reinforce the conclusions from the 1-year EVRA results, that early endovenous ablation of superficial venous reflux is highly beneficial for both patients and health care professionals. These observations indicate that a policy of deferred or delayed endovenous intervention is illogical for patients with venous ulceration.

Long-term outcomes beyond 4 years remain unknown. Because chronic venous hypertension is multifactorial, ulcer recurrence is likely to be a common event in this population, despite endovenous ablation. Thirty percent of participants recruited to the EVRA trial suffered recurrent ulcers during follow-up. Aggressive investigation and treatment of venous outflow obstruction have been advocated, and the use of venous stents to correct nonthrombotic and post-thrombotic deep venous occlusive disease is increasing in popularity but requires robust evaluation.^18,19^ Although there may be a role for deep vein stenting in some patients with venous ulceration, excellent healing outcomes were achieved in the EVRA trial cohort with combined good compression therapy and superficial venous ablation. It should also be noted that patients with concomitant arterial disease, foot ulceration, or those not compliant with compression were not included.

## Conclusion

In this randomized clinical trial, early endovenous ablation of superficial venous reflux in addition to compression therapy reduced time to ulcer healing for primary ulcers. We found no statistical evidence that early endovenous ablation reduces time to first ulcer recurrence, but it was associated with a reduced incidence rate of recurrent ulcers and is highly likely to be cost-effective in the management of venous leg ulceration.

## Notes

Supplement 1. **Trial Protocol**

Supplement 2. **eMethods.** Health Economic Analysis **eFigure 1.** Mortality Rate for Participants in the EVRA Trial **eFigure 2.** Time to Ulcer Recurrence From Randomization **eFigure 3.** Kaplan-Meier Curve for Time to Ulcer Healing for Recurrent Venous Leg Ulcers in the Early-Intervention Group and Deferred-Intervention Group **eFigure 4.** Time to Ulcer Healing for Primary Ulcer **eFigure 5.** Time to First Ulcer Recurrence **eFigure 6.** Cost-Effectiveness Acceptability Curve at 3 Years **eFigure 7.** Sensitivity Analysis Using Monte Carlo Simulation **eTable 1.** EVRA Study: Recruiting Centers **eTable 2.** Summary of Secondary Outcome Measures and Quality-of-Life Tools Used in EVRA Study **eTable 3.** Resource and Unit Costs Utilized for Health Economic Analysis **eTable 4.** Summary of Quality-of-Life Outcomes for Extended Follow-Up **eTable 5.** Use of Hospital and Community Health Services and Non-NHS Expenses During Extended Follow-Up Period **eTable 6.** Undiscounted Costs Per Patient at 3 Years in Each Treatment Group **eTable 7.** Difference in Discounted Total Mean Costs and Discounted QALY at Different Time Horizons Estimated Using Mixed Model **eTable 8.** Difference in Discounted Total Mean Costs and Discounted QALY at Different Time Horizons Estimated Using Mixed Model Per Protocol (a) **eReferences**

Supplement 3. **Data Sharing Statement**
