# Supplementary material for: Long-term Clinical and Cost-effectiveness of Early Endovenous Ablation in Venous Ulceration: A Randomized Clinical Trial
Source: JAMA Surg. 2020 Sep 23;155(12):1113–21. doi: 10.1001/jamasurg.2020.3845 (PMC7512122; doi:10.1001/jamasurg.2020.3845)
Supplement: Supplement 1. — Trial Protocol [file jamasurg-e203845-s001.pdf]

This supplement contains the following items:

1. Original protocol (v1.0), final protocol (v5.0), summary of changes.

2. Original statistical analysis plan (EVRA extension phase, v0.5), final statistical analysis plan (EVRA extension phase, v3.0), summary of changes

Supplement Table of Contents

Protocols **2**

Original protocol (v1.0) 2

Final protocol (v5.0) 33

Summary of changes 70

Statistical analysis plan **72**

Original statistical analysis plan (EVRA extension phase, v0.5) 72

Final statistical analysis plan (EVRA extension phase, v3.0) 89

Summary of changes 105

EVRA (Early Venous Reflux Ablation) ulcer trial

A randomized clinical trial to compare early versus delayed endovenous treatment of superficial venous reflux in patients with chronic venous ulceration.

Version 1.0 19/06/2013

MAIN SPONSOR: Imperial College London

FUNDERS: NIHR HTA

ISRCTN02335796

STUDY COORDINATION CENTRE: Imperial College Trials Unit

NRES reference: 13/SW/0199

	Date	Signature
**Protocol authorised by:**<br>**Name: Professor Alun H Davies**	**27th June 2013**	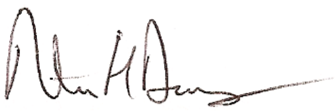

Study Management Group

Chief Investigator: Professor Alun H Davies

Co-investigators: Mr Manjit S Gohel, Mr Richard Bulbulia, Mr Keith R Poskitt, Professor Andrew Bradbury, Professor Nicky Cullum, Miss Sophie R Renton, Mr I Nyamekye

Statistician: Dr Jane Warwick

Health economist: Dr David Epstein

Study Management: Miss Francine M Heatley

Study Coordination Centre

For general queries, supply of study documentation, and collection of data, please contact:

Study Coordinator: Miss Francine M Heatley

Address: Vascular Surgery Research Group , Room 4N12, 4th Floor North Wing

Charing Cross Hospital, Fulham Palace Road, London W6 8RF

Tel: 020 3311 7371

E-mail: f.heatley@imperial.ac.uk

Fax: 0203 3117362

Web address: www.evrastudy.org

Clinical Queries

Clinical queries should be directed to either the Local PI or the Study Coordinator who will direct the query to the appropriate person

Sponsor

Imperial College London is the main research Sponsor for this study. For further information regarding the sponsorship conditions, please contact the Head of Regulatory Compliance at:

Joint Research Compliance Office

Imperial College London and Imperial College Healthcare NHS Trust

Room 5L10C, 5th Floor Lab Block

Charing Cross Hospital

Fulham Palace Road

London, W6 8RF

Tel: 0203 311 0204

Fax: 020 311 0203

Funder NIHR – HTA Rapid Trials grant

This protocol describes the EVRA study and provides information about procedures for entering participants. Every care was taken in its drafting, but corrections or amendments may be necessary. These will be circulated to investigators in the study. Problems relating to this study should be referred, in the first instance, to the Chief Investigator.

This study will adhere to the principles outlined in the NHS Research Governance Framework for Health and Social Care (2nd edition). It will be conducted in compliance with the protocol, UK Clinical Trials Regulations, the Data Protection Act and other regulatory requirements as appropriate.

1. INTRODUCTION

1.1 BACKGROUND

Chronic leg ulcers are open "sores" on the lower limbs situated between the ankles and knees, which fail to heal within 6 weeks. These ulcers represent a source of great discomfort and social isolation to patients who often complain of associated pain, odour and wound discharge. The time taken for the ulcers to heal means that the condition is also particularly frustrating to health carers involved in their management in hospital and community settings. The underlying cause of leg ulceration in over 70% of cases is lower limb venous dysfunction, sometimes evident as varicose veins but often undetectable by visual examination alone[1]. The estimated overall prevalence of active venous ulceration is as high as 1.5 to 1.8 per 1000 population, increasing to 3.8 per 1000 population in those over 40 years of age[2 3]. As patients with venous ulceration usually suffer episodes of recurrence between periods when the ulcer remains healed, the number of patients with a high risk of ulceration may actually be 4-5 fold higher[4]. It should also be noted that with an aging and increasingly obese population[5], the incidence and prevalence of venous ulceration are both likely to increase. Treatment of the condition in the UK produces a substantial cost burden estimated at £400-600 million per annum[6].

Venous ulcers are characterised by protracted healing times. Despite some recent advances in the management of patients with venous ulcers, 24 week healing rates in published randomized trials are around 60-65%[7 8], and the true population healing rates are likely to be significantly lower. Some patients may never heal and those that do heal are at high risk of recurrent ulceration. These poor outcomes are likely to be a reflection of the severe underlying venous dysfunction in this patient group, although inadequate assessment and suboptimal treatment are also likely to be important contributing factors.

1.1.1 Pathophysiology of venous ulceration

The venous circulation of the lower limb has two components, the deep and superficial systems. Blood normally flows from the superficial to the deep veins and is prevented from flowing back down the leg under the influence of gravity by 'one-way' valves along the veins. When these valves become incompetent (leaky), the superficial veins usually become dilated and tortuous (varicose) and the resulting sustained high venous and capillary pressures lead to skin inflammation and ulceration (breakdown of skin). The deep veins also have valves, which may also become incompetent, but are not visible on the skin. Duplex ultrasound studies[9 10 11] on patients in leg ulcer clinics suggest that:

- Around 50% of patients with venous leg ulcers have diseased superficial veins alone, with a further 30-40% having a mixture of superficial and deep venous disease. Both of these groups of patients benefit from correction of their

superficial venous reflux, which has been shown to reduce the risk of ulcer recurrence[12].

- A minority (5-10%) of patients with venous ulcers have diseased deep venous systems only, and are not amenable to surgical correction. These patients are usually treated with compression bandaging alone

Ulcer healing strategies are based on efforts to reduce this leakage (reflux) of blood back down the leg and into the skin, as this is considered the most significant cause of high venous pressure in most patients. Longstanding venous hypertension has been shown to cause a number of changes to the microcirculation in the lower leg, which can contribute to the chronic skin changes or eventual ulceration associated with chronic venous disease[13]. Compression bandaging to the leg (which may need to be re-applied 1-4 times per week) counteracts the gravitational force on the blood, in effect temporarily replacing the incompetent valves[14]. Diseased superficial veins can be surgically removed (open varicose vein surgery) or ablated using endovenous interventions (see below) without harming the overall venous function of the leg, theoretically removing a causative factor for recurrence of the ulcer after the compression bandaging has ceased. The deep vein defects are not generally amenable to surgery.

1.1.2 Treatment options for superficial venous reflux

For over a century, the treatment of superficial venous reflux has involved operative ligation and surgical stripping of the vein and avulsion of bulging varicose veins[15]. Until recent years, open surgery has been considered the definitive treatment option for superficial venous reflux. However, the operation usually requires general anaesthesia and patients often suffer discomfort, bruising and significant time off work in the post-operative period. Long-term studies have also identified significant complications of open surgery including nerve damage and recurrence of varicose veins, seen in over 60% of patients at 11 years in one randomized study[16].

In response to this high complication rate and a growing patient desire for less invasive treatments, a range of novel, minimally invasive endovenous treatment options have been developed and have gained in popularity over the last decade. Interventions such as ultrasound guided foam sclerotherapy (UGFS)[17], endovenous laser (EVLA)[18] or radiofrequency ablation (RFA)[19] can be performed using local anaesthesia in an outpatient setting. These treatments involve cannulation of the vein to be treated (usually under ultrasound guidance) and obliteration of the venous channel by either chemical ablation (using foam sclerosant), or thermal ablation (using a laser or radiofrequency fibre). Numerous randomized studies have demonstrated that endovenous modalities are, at worst, comparable to open surgery in terms of recurrence (and likely to be better), but clearly superior in terms of pain, bruising and other early complications[20-22]. Each of the different endovenous modalities has advantages and potential disadvantages, although all are less invasive than traditional open surgery. This is of particular relevance to patients with

chronic venous ulceration, who are often elderly, have extensive co-morbidities and may be reluctant to undergo surgical procedures involving general anaesthesia. Endovenous techniques can also be performed without discontinuing anti-coagulation therapy, which is increasingly prescribed in this patient population.

1.1.3 Summary of current research

The most significant study of superficial venous intervention in patients with venous ulceration is the ESCHAR study (Barwell, Poskitt; Lancet 2004 & Gohel, Poskitt; BMJ 2007)[7 12]. The study aimed to evaluate the role of traditional superficial venous surgery in reducing ulcer recurrence in patients with open or recently healed venous ulcers. Following prospective observational studies to inform power calculations, a total of 500 patients were randomized to compression therapy alone or compression with open surgery for superficial venous reflux. The group randomized to surgical treatment had significantly lower venous ulcer recurrence rates at 4 years (Figure 1).

Analysis stratified by pattern of venous reflux demonstrated that this clinical benefit was present for patients with isolated superficial venous reflux and patients with superficial and segmental deep reflux. This clearly indicated that the majority of patients with chronic venous ulceration could benefit from superficial venous intervention. As a result, the current optimal management of patients with venous ulceration includes the treatment of refluxing superficial veins to reduce the risk of ulcer recurrence[23].

Analysis of ulcer healing within the ESCHAR trial demonstrated that there was no significant improvement in ulcer healing rates for the group randomized to compression plus surgery (Figure 2). This finding has led many to conclude that treatment of venous reflux does not have a role in patients with open ulcers.

Figure 1. ESCHAR trial – ulcer recurrence

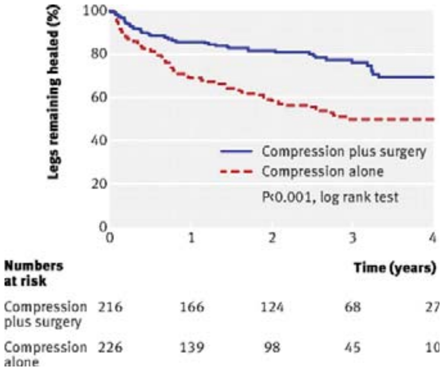

Figure 2. ESCHAR trial – ulcer healing

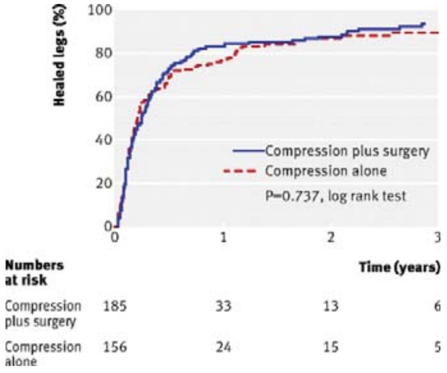

However, the ESCHAR study was designed and powered to assess ulcer recurrence rather than healing, and the statistical power of this trial was further weakened by a high cross-over rate, as around a quarter of patients randomized to surgery subsequently refused to have an operation. This highlights the need for a minimally invasive superficial venous treatment modality in this patient group. In addition, the

median time to treatment within the study was around 2 months, by which time smaller ulcers may have already healed with compression bandaging, and, in many cases, the surgical procedures used were suboptimal when judged by current standards. Consequently, it is plausible that the benefits of treating superficial venous reflux were underestimated in this study, particularly for the assessment of ulcer healing.

In a smaller Dutch randomized trial, 170 patients (200 legs) were randomized to compression alone or compression with surgical treatment of superficial reflux (including subfascial endoscopic perforator surgery – SEPS)[8]. Although results did not reach statistical significance, there was a clear trend towards improved ulcer healing rates and greater ulcer free time in the group randomized to surgery.

Despite the widespread acceptance of endovenous modalities, few prospective studies have been published reporting outcomes after endovenous treatment in patients with leg ulcers. In a prospective study of 186 patients with leg ulceration treated with UGFS, the ulcer healing rate was over 70% and the patient acceptability of treatment was excellent (Poskitt et al)[24]. In a further study of foam sclerotherapy in 130 patients, a healing rate of 82% was achieved (Bradbury et al)[25]. Whilst these small non-randomized studies lend support to our hypothesis that early intervention to correct superficial venous reflux will promote ulcer healing, a large randomized trial is required to provide reliable evidence and thus change practice.

1.2 RATIONALE FOR CURRENT STUDY

Whilst the management of patients with venous ulcers has evolved in recent years and ulcer healing and recurrence rates have shown some improvement, we believe that there is a strong argument in favour of this study at this time for the following reasons:

- The prevalence of venous ulceration is likely to increase, particularly with an aging and increasingly obese population. In view of the significant financial and psychosocial costs of venous ulceration, it is imperative that the optimal treatment strategies are identified.
- Despite numerous studies of topical ulcer treatments, the only treatment shown to improve venous ulcer healing is compression bandaging. Compression supports the venous circulation, but is poorly tolerated by some patients and does not address the underlying problem of venous reflux. The intervention in this proposal involves treating the underlying anatomical venous disorder using effective, minimally invasive endovenous interventions and offers a logical, deliverable and long-term approach to reducing venous hypertension.
- The treatment of superficial venous reflux has been transformed in recent years through the widespread use of minimally invasive, endovenous interventions, which patients find more acceptable than traditional open surgery.

- Ablation of superficial reflux should be considered in all patients with leg ulcers and superficial venous reflux, but if early intervention is associated with moderate improvements in ulcer healing compared to deferred intervention (i.e. post-healing), significant cost savings could be realised.
- Patients find venous leg ulcers painful, distressing and a significant inhibition to normal, independent life. Interventions to reduce the time to healing could reduce patient distress and significantly improve quality of life.

Therefore, we believe that there is a cogent argument for conducting this trial at this time. Non-randomized studies suggest that outcomes may be improved by treating underlying superficial reflux using the latest technologies, but there is no robust evidence to support early intervention. The research team has a strong track record in relevant research areas and includes clinicians and researchers who successfully completed the landmark clinical trial on which this proposal is based (ESCHAR trial), and numerous other high impact clinical trials evaluating treatments in venous ulceration.

2. OBJECTIVES

2.1 PRIMARY OBJECTIVE

What is the clinical and cost effectiveness of early endovenous treatment of superficial venous reflux in addition to standard care compared to standard care alone in patients with chronic venous ulceration?

2.2 SECONDARY OBJECTIVES

To investigate:

- The ulcer free time to 1 year
- The technical success of endovenous interventions

3. PARTICIPANT ENTRY

3.1 PRE-REGISTRATION EVALUATIONS

Prior to commencing, information will be disseminated to GP practices in each recruiting region and meetings will be arranged with key community nursing staff and at leg ulcer clinics to promote the trial. Patients would be referred to secondary care as part of the standard care pathway.

At the referral visit patients will be given an appropriate time period to consider participation (at least 24 hours). Written consent will be obtained from those patients who agree to participate and randomization will be performed using the online service. For patients randomized to endovenous ablation of superficial venous reflux, a date for intervention will be booked as soon as possible (i.e. within 2 weeks). At each recruiting centre, an online log of all screened patients will be kept using the InForm system. Basic demographic data and reasons for non-eligibility will be recorded. Whilst participant baseline characteristics may vary slightly across recruiting sites, randomized treatment allocation will allow reliable assessment of the effects of early versus delayed endovenous ablation in ulcer healing.

3.2 INCLUSION CRITERIA

- Current leg ulceration of greater than 6 weeks, but less than 6 months duration
- Able to give informed consent to participate in the study after reading the patient information documentation
- Patient age > 18 years
- Ankle Brachial Pressure Index (ABPI) $\geq$ 0.8
- Superficial venous disease on colour duplex assessment deemed to be significant enough to warrant ablation by the treating clinician (either primary or recurrent venous reflux)

Patients who cannot speak / understand English will be eligible for inclusion and informed consent will be obtained with assistance from translation services as per standard clinical practice. In view of the lack of cross-cultural validation for quality of life tools, only healing outcome data will be collected.

3.3 EXCLUSION CRITERIA

- Presence of deep venous occlusive disease or other conditions precluding superficial venous intervention (at the discretion of local research team)
- Patients who are unable to tolerate any multilayer compression bandaging will be excluded. However, concordance with compression therapy can be variable for patients at different times. Patients who are generally compliant with compression, but unable to tolerate the bandages for short periods will still be eligible to inclusion. A period of non-compliance with compression bandages will not be considered a protocol violation, but a normal variation within the spectrum of ‘standard therapy’.

- Inability of the patient to receive prompt endovenous intervention by recruiting centre
- Pregnancy (female participants of reproductive age will be eligible for inclusion in the study, subject to a negative pregnancy test prior to randomisation)
- Leg ulcer of non-venous aetiology (as assessed by responsible clinician)
- If patient is deemed to require skin grafting they cannot be included

4. STUDY DESIGN

The EVRA ulcer trial is a pragmatic; multicentre randomized clinical trial with participants randomized 1:1 to either:

1. ‘Standard’ therapy consisting of multilayer elastic compression bandaging with deferred treatment of superficial reflux (usually once the ulcer has healed)
2. Early endovenous treatment of superficial venous reflux (within 2 weeks) in addition to standard therapy

The study design is summarised in Figure 3 below.

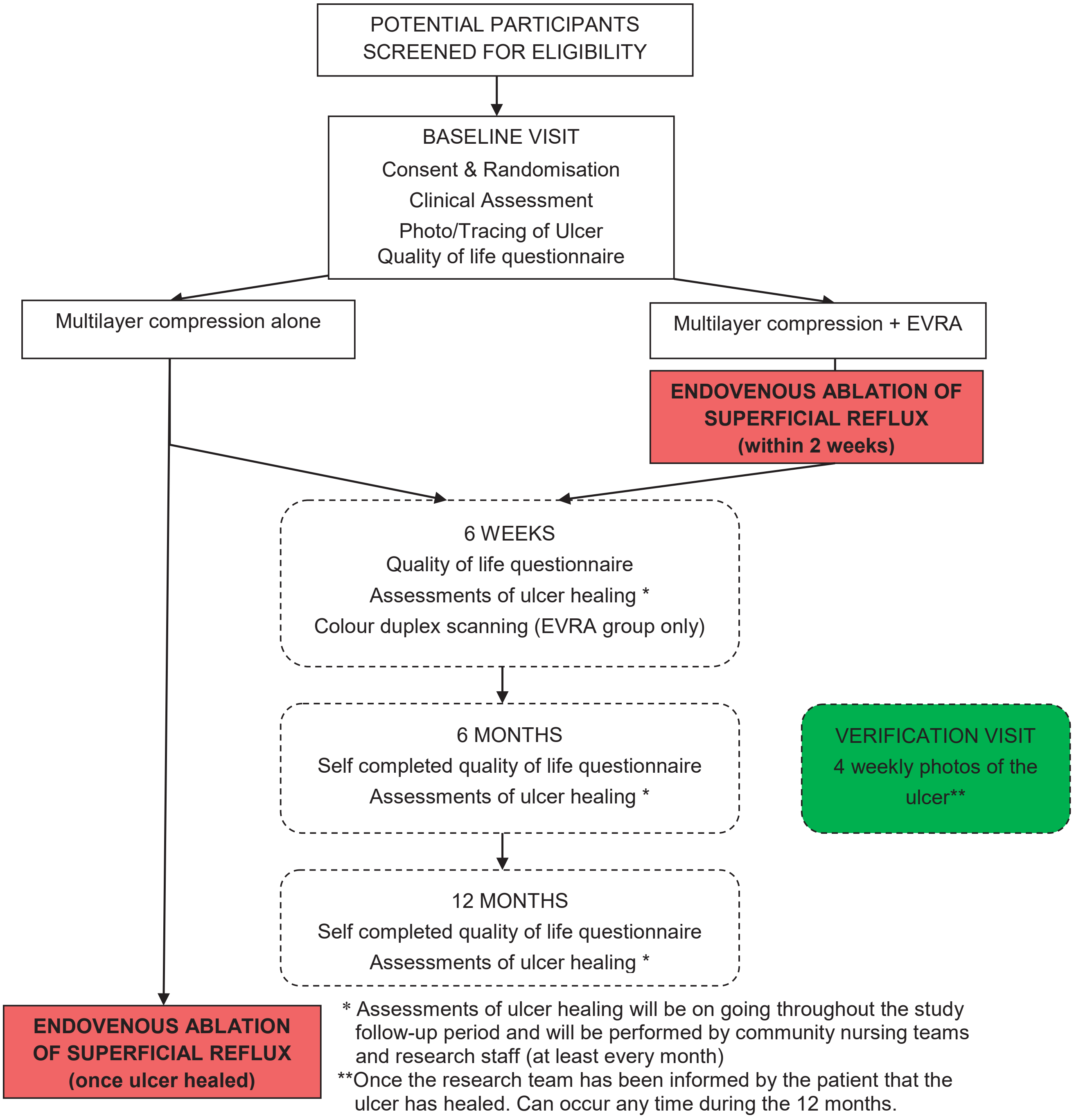

4.1 PATIENT RANDOMIZATION

The normal clinical team will make initial contact with potentially eligible patients at the referral visit.

Those who consent will be registered on the InForm ITM (Integrated Trial Management) System, a web-based data entry system, which is maintained by ICTU, and their eligibility for the study confirmed. A randomization list will be loaded onto the InForm system for each centre (as stratification will be by centre) before recruitment commences, having been prepared in advance by a statistician who is independent of the study. Each potential participant, if confirmed to be eligible, will be assigned the next available entry in the appropriate randomization list (i.e. without foreknowledge). Thereafter, treatment allocation will not be blinded (with the exception of assessment of ulcer healing – see 4.3.1). For patients with bilateral venous ulceration, the worst leg (according to the patient) will be designated the ‘reference leg’. Interventions may be performed on both legs, if deemed appropriate by the responsible clinician.

4.2 STUDY SETTING

Eligible patients with chronic venous ulcers will be recruited from the following centres:

1. Imperial College Healthcare NHS Trust (PI: Professor AH Davies)
2. Cambridge University Hospitals NHS Foundation Trust (PI: Mr MS Gohel)
3. Gloucestershire Hospitals NHS Foundation Trust (PI: Mr KR Poskitt)
4. West Midlands Vascular Research Collaborative (Heart of England NHS Trust; University Hospital Birmingham NHS Trust; City and Sandwell NHS Trust; Russell’s Hall Hospital NHS Trust, Dudley; and New Cross Hospital NHS Trust, Wolverhampton) (PI: Professor A Bradbury)
5. North West London Hospitals NHS Trust (PI: Miss SR Renton)
6. Worcestershire Acute Hospitals NHS Trust (PI: Mr I Nyamekye)

4.3 STUDY OUTCOME MEASURES

4.3.1 Primary outcome measure

The primary outcome measure will be time to ulcer healing (from date of randomization to date of healing). For the purposes of this study, ulcer healing is defined as complete re-epithelialisation of all ulceration on the randomized leg. Community or hospital healthcare staff, depending on the local model of care, will perform assessment of ulcer healing.

Data on the status of the reference leg will be collected throughout the study by research staff scrutinising community medical / nursing records and contacting the patient / community nursing teams by telephone (on a monthly basis at least).

If either the community nursing / medical staff or the patient believe that ulcer healing (defined as complete re-epithelialisation of the ulcerated leg) has been achieved, they will be asked to contact the local research centre immediately. This notification of possible ulcer healing will constitute a ‘trigger’ for the research staff at the recruiting centre to arrange an urgent verification assessment by a member of the healthcare team (within 1 week).

Verification will be by clinical assessment and digital photography, to be repeated weekly for 4 weeks. The digital images will be evaluated by two blinded expert assessors in order to ascertain the date of healing, which will be considered the primary healing end-point. Disagreements will be resolved through discussion with involvement of a third blinded expert reviewer if necessary. This approach will be applied to patients in both treatment arms and is consistent with the methods utilized in other large HTA funded leg ulcer trials (e.g. VenUS IV). Legs deemed to have an open ulcer on clinical assessment would continue within the study. If healing is confirmed by clinical and blinded photograph assessments at the first verification visit, the date of healing notification (by patient or community nurse) will be taken as the date of ulcer healing.

4.3.2 Secondary outcome measures

A number of secondary outcome measures will be evaluated in the EVRA study:

1. Ulcer Healing Rate: Healing rate will be evaluated in addition to time to ulcer healing to allow comparison with other published studies.
2. Ulcer Free Time: Will be calculated up to 1 year for each study arm. This will allow a very practical and easily understood assessment of the clinical difference between the 2 arms of the study. This will also allow comparison with other studies that have reported this outcome. In order to facilitate accurate calculation of ulcer free time, clinical follow up will be continued after ulcer healing up to 1 year after randomisation.
3. Quality Of Life (QoL): Disease specific (AVVQ) and generic (EQ5D & SF36) quality of life assessments will be compared at 6 weeks post randomisation, 6 months and 12 months. The 6-week questionnaire will be given to the patient at the follow-up appointment, whereas other QoL questionnaires will be sent to the patient. AVVQ is the most widely utilised disease specific QoL tool in venous disease and has been extensively validated. A score out of 100 points is calculated, with a higher score indicating more severe QoL impairment. Changes in QoL scores will offer a comparison with other studies and, in the standard treatment arm, will allow an assessment of the natural history of venous ulceration treated with compression.
4. Health Economic Assessment: Cost items in hospital and community care will be recorded for each patient. Standard HRG published tariffs will be used to calculate overall costs. A standard tariff will be applied for each bandage change, although additional treatments administered for the treatment of symptoms or

complications directly related to venous ulceration will be included. Utilities (QALYs) will be calculated from generic QoL questionnaire and cost-effectiveness will be analysed.

5. Other Markers Of Clinical Success: The Venous Clinical Severity Score (VCSS) will be assessed at 6 weeks. In addition, the incidence of complications related to the endovenous intervention as well as the presence of residual / recurrent varicose veins will also be assessed at 6 weeks.

4.4 DURATION OF FOLLOW-UP

In the present study, participants will be followed-up until either:

1. 1 year post-randomization
2. Patient choice to withdraw from the study. Patients who no longer wish to complete quality of life questionnaires will be asked if they would object to the use of healing status data (to contribute to the primary outcome)
3. Death

In order to allow assessment of ulcer free time to 1 year, patients with healed ulcers will be evaluated using telephone follow-up (performed by staff at the recruiting centre) on a monthly basis until 1 year. The aim of the telephone follow-up will be to confirm that the ulcer remains healed, or in cases of ulcer recurrence, to ascertain the date of recurrence and of subsequent healing. More prolonged post-intervention follow-up for several years is required to obtain reliable long-term recurrence rates in both treatment groups. Accordingly, participants will be asked to consent to long-term follow-up at the outset, and funding for an extension to EVRA will be sought in due course.

4.5 STUDY DURATION

The EVRA study will take four years to complete. The overall study timetable is summarised in Figure 4.

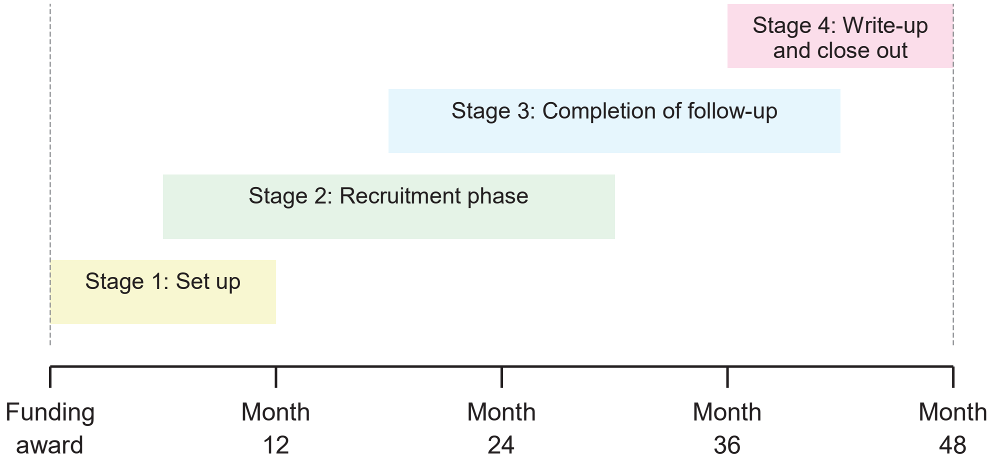

Figure 4. EVRA study Gantt chart

5. DETAILS OF INTERVENTIONS

5.1 VARIATIONS IN ENDOVENOUS INTERVENTIONS

A wide range of endovenous treatment modalities are now available and in widespread use for the ablation of superficial venous reflux. These include:

- Endovenous thermal ablation using laser or radiofrequency
- Ultrasound guided foam sclerotherapy (UGFS)
- Other endovenous interventions such as mechanochemical ablation, steam ablation and glue
- Any combination of the above treatments

In addition to the different modalities in use, the treatment strategy may also vary between institutions and between individual clinicians within the same department. Variations may occur in:

- Site of vein cannulation (and therefore the length of vein ablated)
- Location of treatment ('office' or clinic based versus operating theatre)
- Treatment strategy for sub-ulcer venous plexus (to ablate or not)
- The treatment of visible varicose veins (no treatment, UGFS or surgical avulsion) and the timing of any intervention

5.2 STANDARDISATION OF INTERVENTIONS IN EVRA STUDY

With the lack of consensus on a single, optimal endovenous treatment strategy for superficial reflux in patients with leg ulceration, perfect standardisation of interventions will be impossible. All endovenous interventions should be performed as deemed to be 'optimal' by the treating clinician for each individual patient, with the following stipulations:

1. The endovenous strategy must include ablation of the main truncal venous reflux
2. Truncal venous reflux should be treated to the lowest point of incompetence, where possible
3. Significant (as deemed by the treating clinician) residual / recurrent superficial reflux on the 6 week duplex scan, should be ablated
4. Patients should continue with multilayer compression immediately after treatment

5.3 STANDARDISATION OF COMPRESSION

Patients will receive the standard compression used in the individual centres prior to ulcer healing following randomisation (this will include four layer bandaging, three layer bandaging, European short stretch, stockings). Post healing the patients will be given compression hosiery in line with local policy.

5.4 FURTHER TREATMENT FOR COMPRESSION ALONE ARM

Patients randomised to multilayer compression alone can be offered endovenous treatment of superficial reflux once healing has been confirmed (see 4.3.1). Endovenous ablation should be performed as per standard practice in the treating centre and details of this will be recorded. Endovenous intervention may also be offered if there is clinical deterioration in the active leg ulcer and it is clinically felt that the patient may benefit from early intervention. This will be recorded on the electronic case report form.

6. ASSESSMENT AND FOLLOW-UP

6.1 PATIENT IDENTIFICATION

Patients will be referred to secondary care for evaluation of the management of their leg ulcer as part of the standard pathway of care.

6.2 REFERRAL VISIT

At the initial visit the patient will be evaluated by clinical assessment and colour duplex examination, which is part of the normal investigation of a patient with leg ulceration. Dependant on the results of these tests, the patient will be asked if they would consider taking part in the trial and approached for consent. The patient will be given a minimum of 24 hours to consider the trial and if willing to participate will return to the leg ulcer clinic to give consent and undergo a baseline visit.

6.3 BASELINE VISIT

Patients will undergo detailed clinical assessment by the research nurse as part of the baseline evaluation (see *Appendix 1*). Recorded assessments will include:

- Demographic details (age, sex, ethnicity)
- Pregnancy test for woman of child bearing potential
- General clinical details (body mass index, ankle brachial pressure index – performed within previous 4 weeks, comorbidities, medication history)
- Ulcer details (duration, progression, previous ulcer history, size of current ulcer – using photography and planimetry)
- Assessment of range of ankle movement
- Details of venous disease (previous deep vein thrombosis, previous venous interventions, pattern of venous reflux on duplex)

Additional assessments will include:

- Assessment of Clinical, Etiologic, Anatomic, Pathophysiological (CEAP) score
- Assessment of venous clinical severity score (VCSS)
- Disease specific (Aberdeen varicose vein questionnaire – AVVQ) and generic (EuroQuol 5D – EQ5D & short form (SF) 36) quality of life assessments

At this visit, eligible and consenting patients will be randomised into the trial.

6.4 FOLLOW-UP ASSESSMENTS

Randomized patients will undergo routine leg ulcer care in community or hospital (or both) settings, in accordance with the local standard. This will equate to wound reviews and dressing changes ranging between once and 4 times per week (depending on the ulcer). The exact nature of dressings and date of dressing change will be documented by community or hospital healthcare professionals. This will

allow an accurate record of the dressing types used and will be collected and verified by the research nurse.

In addition, the following assessments will be conducted:

6.4.1 6-week clinic visit

- Clinical assessment
- In the compression plus early venous reflux ablation group, venous duplex scanning will be performed at 6 weeks post-randomization to verify anatomical treatment success. Depending on the results of the scan, the decision to perform further superficial venous interventions will be left to the discretion of the responsible clinical staff. Irrespective of the number and timing of venous interventions, all analyses will be performed on intention to treat.
- Wound tracing and photo
- Assessments of disease specific and generic quality of life (AVVQ, EQ5D & SF36) by means of self completed questionnaire

6.4.2 Further follow-up

- Assessments of disease specific and generic quality of life (AVVQ, EQ5D & SF36) by means of self completed questionnaire at 6 months and 12 months post-randomization (sent to the patient).
- The research team will perform monthly telephone evaluation of the patient and access the community notes or telephone the community nurses in order to collect and verify the data collected.
- Once the research team has been informed that the ulcer has healed the patient will undergo an urgent verification visit

6.5 URGENT VERIFICATION VISIT

- A member of the local research team will perform the four verification visits to confirm healing. Photographs will be taken and send to the Trials Unit for independent verification.

7. STATISTICS AND DATA ANALYSIS

Data and all appropriate documentation will be stored for a minimum of 10 years after the completion of the study, including the follow-up period.

7.1 SAMPLE SIZE CALCULATION

The sample size calculation for this study was based on the primary outcome of ulcer healing. The ESCHAR trial was a similar randomized study, which published the final results in 2007 (see 1.1.3). A total of 500 patients with open or recently healed venous ulcers were randomized to standard therapy alone or standard therapy plus open surgery for superficial venous reflux. The study was powered and designed to evaluate differences in ulcer recurrence (rather than healing). Consequently, the median time from randomization to treatment delivery was over 7 weeks. Nevertheless, the 24-week healing rate in patients randomized to standard treatment (compression alone) was approximately 60%. Two recent prospective studies evaluating the early treatment of superficial venous reflux suggested that the 24-week healing rate may be as high as 82%[24 25].

In order to calculate a sample size for this study, we estimate a benefit associated with early treatment of around 15%. To identify a difference in 24-week healing rates of 15% between the two groups with 90% power will therefore require 208 subjects (68 healed leg ulcers) per group (log-rank test). With 10% dropout the study will therefore require 462 subjects (231 in each arm). To incorporate further allowances for protocol violations and unexpected dropouts, the target sample size will be 500 patients.

7.2 PLANNED ANALYSES

Basic descriptive methods will be used to present the data on study participants, trial conduct, clinical outcomes and safety (in total and for each study group separately). The primary outcome will be time to complete healing and we will test the hypothesis that there is no difference in this between the control and intervention groups using a log-rank test (two-tailed, 5% significance level). Kaplan-Meier survival curves will also be presented and as a subsidiary analysis we will investigate the effect of study centre, participant age, ulcer size and chronicity on time to complete healing using Cox regression. To adjust for potential surgeon and centre effects, surgeon and centre will be included in the Cox regression analysis as random effects. All analyses will be on an intention-to-treat basis. Non-compliance with allocated interventions and other protocol violations will be kept to a minimum. Accordingly, per-protocol analyses are not envisaged, and the chief emphasis will be on the overall result on time to ulcer healing.

7.3 HEALTH ECONOMIC ANALYSIS

The economic evaluation will be based on both a modelling exercise and a patient level in-trial analysis. The analysis will be performed from the perspective of the NHS and society. The economic model will be developed from the model used for another HTA funded project (REACTIV trial)[26]. The model will assess the relative cost-effectiveness (assessed in terms of incremental cost per QALY), of the treatment strategies. The trial data will inform the model and further data (including that for other relevant comparators) will come from the literature and other data sources. Use of secondary and primary care patient resource use and EQ-5D responses will come from the trial. They will be collected by case note review and questionnaires completed at baseline, 6 and 12 months. Unit costs will be based on nationally available data and study-specific estimates. QALYs will be estimated using responses to the EQ-5D. The results of the economic model will be supplemented by an in-trial analysis. The trial analysis will use the estimates of costs and QALYs estimated for each trial participant to calculate the incremental cost-effectiveness ratios for the 12-month follow-up. The results of the analyses will be presented as estimates of mean incremental costs, effects, and, incremental cost per QALY. Sensitivity analysis will be conducted for both model and trial based evaluations. The results of the base case and sensitivity analyses will be presented as mean estimates and as cost-effectiveness acceptability curves (CEACs).

7.4 INTERIM ANALYSES: ROLE OF THE DATA MONITORING COMMITTEE

During the study, interim analyses of all related SAEs and other study outcomes will be supplied in strict confidence to the independent Data Monitoring Committee (DMC). The DMC will request such analyses at a frequency relevant to the stage of the study (typically at 12 monthly intervals with a Chairman's review every 6 months) or in response to emerging data from other trials. Unless advised by the DMC in response to clear evidence of benefit or hazard, the Steering Committee, collaborators, participants and all study staff (except those who provide the confidential analyses to the DMC) will remain blind to the interim results until the end of the study.

In the light of these interim analyses and any other information considered relevant, the DMC will advise the Steering Committee if, in their view, the randomized comparisons in the study have provided both (i) "proof beyond reasonable doubt" * that early correction of superficial venous reflux improves ulcer healing; and (ii) evidence that might reasonably be expected to influence materially patient management.

* Appropriate criteria of proof beyond reasonable doubt cannot be specified precisely, but a difference of at least 3 standard deviations in an interim analysis for healing may be needed before stopping the trial prematurely. Furthermore, this criterion has the practical advantage that the exact number of interim analysis would be of little importance, so no fixed schedule is proposed.

The DMC would also be expected to advise the Steering Committee if clear evidence emerged of an adverse effect on intervention-related SAEs, and if this hazard seemed likely to outweigh any potential benefit.

7.5 LOSSES TO FOLLOW-UP AND PROTOCOL VIOLATIONS

The primary assessment involves intention-to-treat analysis. Therefore, strenuous efforts will be made to ensure that only patients willing to undergo either immediate or delayed superficial venous ablation and compression bandaging are randomized. Monthly reports of protocol violations will be provided by local sites to the trial coordinators, who reserve the right to suspend or exclude sites in the event of wilful protocol violations. Similarly, efforts will be made to obtain complete follow-up for all randomized participants (irrespective of whether or not they underwent allocated treatment). For those participants unable or unwilling to attend follow-up appointments, home-visits or follow-up by community nurses may be considered.

We appreciate that a high rate of protocol violations was seen in previous trials of venous ulceration (including the ESCHAR trial). This is likely to reflect the reluctance and apprehension of elderly patients to undergo surgical interventions involving general anaesthesia. The modern management of superficial venous disease involves a range of minimally invasive, endovenous modalities that can be performed using local or no anaesthesia. Procedures are performed on an outpatient basis and can be completed in around 30 minutes. Published studies of endovenous interventions have demonstrated excellent patient satisfaction and few treatment refusals. Due to the published evidence and extensive personal experience among the research team, we believe that the rate of participation will be higher and rate of protocol violations will be lower than previous studies.

The following will be recorded as protocol deviations:

1) Patients randomised to multilayer compression plus early venous reflux ablation, who receive endovenous intervention more than two weeks from randomization.
2) Patients who are non-compliant with compression bandaging, defined as use <75% of the prescribed duration.
3) Patients randomised to compression bandaging alone who undergo endovenous ablation prior to verified healing.

8. ADVERSE EVENTS

8.1 REPORTING PROCEDURES

All serious adverse events and all intervention-related adverse events should be reported. Depending on the nature of the event the reporting procedures below should be followed. Any questions concerning adverse event reporting should be directed to the Chief Investigator in the first instance.

8.2 RELATED ADVERSE EVENTS

Patients randomised to early venous intervention have the potential risks of treatment. Competent, experienced medical staff will perform all procedures and every effort will be made to prevent adverse effects.

Radiofrequency or laser ablation may cause:

- some short-term side effects such as numbness or pins and needles (paraesthesia).
- some tightness in your legs and the affected areas may be bruised and painful.
- nerve injury is also possible, but usually only temporary.

Sclerotherapy can have side effects, including:

- blood clots in other leg veins (DVT)
- headaches
- changes to skin colour, such as, brown patches over the treated veins
- fainting
- temporary vision problems

After any of these procedures, it is possible the patient may develop a painful lump over the varicose veins, known as phlebitis, which may require treatment with antibiotics and/or drainage.

8.3 NON SERIOUS ADVERSE EVENTS

All such events, which are judged by the local PI to be related to the interventions, whether expected or not, should be recorded.

8.4 SERIOUS ADVERSE EVENTS

In addition to clinical assessments, patients will be contacted on a monthly basis by telephone for the duration of the study to identify any additional treatments, admissions or other complications related to their leg ulceration. Unrelated serious adverse events will also be recorded and reported in accordance with the Good Clinical Practice guidance. Serious adverse events (SAE) are defined as those adverse events that: result in death; are life-threatening; require in-patient hospitalisation or prolongation of existing hospitalisation; result in persistent or significant disability or incapacity; result in congenital anomaly or birth defect; are

cancer; or are other important medical events in the opinion of the responsible investigator (i.e. not life threatening or resulting in hospitalisation, but may jeopardise the participant or require intervention to prevent one or more of the outcomes described previously).

All SAEs reported by participants at (or between) each follow-up visit will be recorded by local researchers in the clinical research form. Any SAE that is considered, with a reasonable probability, to be due to study intervention (i.e. superficial venous ablation) should be reported to the local PI (or their designated deputy) and to the trial coordinator. Such intervention-related SAEs will be reported by the trial coordinators to the Sponsor, Chair of the Data Monitoring Committee and to the relevant Ethics Committee.

Contact details for reporting Intervention-related SAEs

Fax: 0203 311 7362, attention Francine Heatley

Please send SAE forms to: Francine Heatley

Tel: 0203 311 7371 (Mon to Fri 09.00 – 17.00)

9. REGULATORY ISSUES

9.1 ETHICS APPROVAL

After approval from the Research Ethics Committee, the study must be submitted for Site Specific Assessment (SSA) at each participating NHS Trust. The Chief Investigator will require a copy of the Trust R&D approval letter before accepting participants into the study. The study will be conducted in accordance with the recommendations for physicians involved in research on human subjects adopted by the 18th World Medical Assembly, Helsinki 1964 and later revisions.

9.2 CONSENT

Consent to enter the study must be sought from each participant only after a full explanation has been given, an information leaflet offered and time allowed for consideration. Signed participant consent should be obtained. The right of the participant to refuse to participate without giving reasons must be respected. After the participant has entered the study the clinician remains free to give alternative treatment to that specified in the protocol at any stage if he/she feels it is in the participant's best interest, but the reasons for doing so should be recorded. In these cases the participants remain within the study for the purposes of follow-up and data analysis. All participants are free to withdraw at any time from the protocol treatment without giving reasons and without prejudicing further treatment.

9.3 CONFIDENTIALITY

The Chief Investigator will preserve the confidentiality of participants taking part in the study and is registered under the Data Protection Act.

9.4 INDEMNITY

Imperial College London holds negligent harm and non-negligent harm insurance policies, which apply to this study.

9.5 SPONSOR

Imperial College London will act as the main Sponsor for this study. Delegated responsibilities will be assigned to the NHS trusts taking part in this study.

9.6 FUNDING

The study is funded by the NIHR as part of the HTA programme.

9.7 QUALITY ASSURANCE AND CONTROL

The study may be subject to inspection and audit by Imperial College London under their remit as sponsor and other regulatory bodies to ensure adherence to GCP and the NHS Research Governance Framework for Health and Social Care (2nd edition). Quality Control will be performed according to the requirements of the Risk Assessment performed by ICTU. The study may be audited by a Quality Assurance representative of the Sponsor. All necessary data and documents will be made available for inspection.

10. STUDY MANAGEMENT

The study will be coordinated by a trial manager based at ICTU reporting to the Clinical Coordinators (MG and RB) and the Chief Investigator (AD). The Clinical Coordinators will liaise with local principal investigators (L-PI) to ensure that the trial is conducted locally according to protocol and in an expeditious manner. The organisational structure and responsibilities are outlines below.

10.1 PRINCIPAL INVESTIGATORS

The chief investigator and clinical coordinators have overall responsibility for:

- Design and conduct of the study
- Preparation of the Protocol and subsequent revisions
- Managing the Trial Coordinating Centre
- Development of SOPs

10.2 TRIAL STEERING COMMITTEE

A Trial Steering Committee (TSC) will be established in line with HTA guidance, consisting of the chief investigator, clinical coordinators, trial manager, trial statistician, patient representative, an independent chair and at least 1 other independent member will be formed and will meet on a 6-monthly basis to discuss trial progress. The TSC is responsible for:

- Agreement of the final Protocol
- Agreeing the Data Analysis Plan
- Reviewing progress of the study and, if necessary, agreeing changes to the Protocol
- Reviewing new studies that may be of relevance
- Review and approval of study reports

10.3 DATA MONITORING COMMITTEE

The independent Data Monitoring Committee (DMC) will be established in line with HTA guidance will focus on the rights, safety and well being of study participants. DMC responsibilities are:

- Reviewing unblinded interim data according to the schedule outlined in the Protocol
- Advising the Steering Committee if, in their view, the randomized data provide evidence that may warrant early termination for either safety or efficacy.

10.4 TRIAL COORDINATING CENTRE

The Trial Coordinating Centre (TCC) is responsible for the overall coordination of the Study, including:

- Study planning and organisation of Steering Committee meetings
- Agreement of each local recruitment plan
- Contractual issues with local study sites
- Ethics Committee applications
- Design, implementation and maintenance of IT systems for the study
- Auditing and monitoring of overall progress of the study
- Clinical safety monitoring (including the reporting of all "related" SAEs to the Chair of the DMC and Ethics Committee)
- Liaison with the Data Monitoring Committee and (where appropriate) with regulatory authorities and other outside agencies
- Responding to technical and administrative queries from local study sites

10.5 LOCAL STUDY SITES

The local principal investigators (L-PI) and clinical staff at the local study sites are responsible for:

- Obtaining local R&D and management approval (aided by the Trial Coordinating Centre)
- Provision of adequate clinic space and the identification of potentially eligible participants
- Conducting study procedures and follow-up according to study protocol
- Dealing with routine enquiries from participants and their families
- Obtaining appropriate information to confirm potential primary and secondary study endpoints
- Attend annual EVRA Study Collaborator Meetings to discuss study progress

11. DOCUMENT RETENTION

Data will be stored for a minimum of 10 years following completion of this trial. Data generated by this work will be processed in accordance with the Data Protection Act 1998.

12. PUBLICATION POLICY

The findings will be disseminated to General Practitioners, nursing staff, surgeons and other health care professionals at regular research and educational meetings organised at local, regional, national and international levels. All analyses will be performed in compliance with a predefined analysis plan. The chief investigator, clinical coordinators and trial coordinator will be responsible for drafting the main reports from the study. Draft copies of any manuscripts will be provided to local principal investigators at each local study site, TSC members and all other collaborators for review prior to publication. The results will be put forward for critical peer review with a view to publication in relevant medical and nursing journals.

13. REFERENCES

1. Obermayer A, Garzon K. Identifying the source of superficial reflux in venous leg ulcers using duplex ultrasound. *Journal of vascular surgery* 2010;52(5):1255-61.
2. Graham ID, Harrison MB, Nelson EA, Lorimer K, Fisher A. Prevalence of lower-limb ulceration: a systematic review of prevalence studies. *Adv Skin Wound Care* 2003;16(6):305-16.
3. Callam MJ, Ruckley CV, Harper DR, Dale JJ. Chronic ulceration of the leg: extent of the problem and provision of care. *Br Med J (Clin Res Ed)* 1985;290(6485):1855-6.
4. Callam MJ, Harper DR, Dale JJ, Ruckley CV. Chronic ulcer of the leg: clinical history. *Br Med J (Clin Res Ed)* 1987;294(6584):1389-91.
5. Centre THaSCI. Statistics on obesity, physical activity and diet: England, 2012. 2012.
6. Laing W. Chronic Venous Diseases of the Leg. In: Economics OoH, editor. London, 1992.
7. Barwell JR, Davies CE, Deacon J, Harvey K, Minor J, Sassano A, et al. Comparison of surgery and compression with compression alone in chronic venous ulceration (ESCHAR study): randomised controlled trial. *Lancet* 2004;363(9424):1854-9.
8. van Gent WB, Hop WC, van Praag MC, Mackaay AJ, de Boer EM, Wittens CH. Conservative versus surgical treatment of venous leg ulcers: a prospective, randomized, multicenter trial. *Journal of vascular surgery : official publication, the Society for Vascular Surgery [and] International Society for Cardiovascular Surgery, North American Chapter* 2006;44(3):563-71.
9. Grabs AJ, Wakely MC, Nyamekye I, Ghauri AS, Poskitt KR. Colour duplex ultrasonography in the rational management of chronic venous leg ulcers. *The British journal of surgery* 1996;83(10):1380-2.
10. Adam DJ, Naik J, Hartshorne T, Bello M, London NJ. The diagnosis and management of 689 chronic leg ulcers in a single-visit assessment clinic. *Eur J Vasc Endovasc Surg* 2003;25(5):462-8.
11. Tassiopoulos AK, Golts E, Oh DS, Labropoulos N. Current concepts in chronic venous ulceration. *Eur J Vasc Endovasc Surg* 2000;20(3):227-32.
12. Gohel MS, Barwell JR, Taylor M, Chant T, Foy C, Earnshaw JJ, et al. Long term results of compression therapy alone versus compression plus surgery in chronic venous ulceration (ESCHAR): randomised controlled trial. *Bmj* 2007;335(7610):83.
13. Browse NL, Burnand KG. The cause of venous ulceration. *Lancet* 1982;2(8292):243-5.
14. Ibegbuna V, Delis KT, Nicolaides AN, Aina O. Effect of elastic compression stockings on venous hemodynamics during walking. *Journal of vascular surgery : official publication, the Society for Vascular Surgery [and] International Society for Cardiovascular Surgery, North American Chapter* 2003;37(2):420-5.
15. van den Bremer J, Moll FL. Historical overview of varicose vein surgery. *Annals of vascular surgery* 2010;24(3):426-32.
16. Winterborn RJ, Foy C, Earnshaw JJ. Causes of varicose vein recurrence: late results of a randomized controlled trial of stripping the long saphenous vein. *Journal of vascular surgery : official publication, the Society for Vascular*

Surgery [and] International Society for Cardiovascular Surgery, North American Chapter 2004;40(4):634-9.
17. O'Hare JL, Earnshaw JJ. Randomised clinical trial of foam sclerotherapy for patients with a venous leg ulcer. *Eur J Vasc Endovasc Surg* 2010;39(4):495-9.
18. Darwood RJ, Gough MJ. Endovenous laser treatment for uncomplicated varicose veins. *Phlebology* 2009;24 Suppl 1:50-61.
19. Gohel MS, Davies AH. Radiofrequency ablation for uncomplicated varicose veins. *Phlebology* 2009;24 Suppl 1:42-9.
20. Carradice D, Mekako AI, Mazari FA, Samuel N, Hatfield J, Chetter IC. Randomized clinical trial of endovenous laser ablation compared with conventional surgery for great saphenous varicose veins. *The British journal of surgery* 2011;98(4):501-10.
21. Subramonia S, Lees T. Randomized clinical trial of radiofrequency ablation or conventional high ligation and stripping for great saphenous varicose veins. *The British journal of surgery* 2010;97(3):328-36.
22. van den Bos R, Arends L, Kockaert M, Neumann M, Nijsten T. Endovenous therapies of lower extremity varicosities: a meta-analysis. *Journal of vascular surgery : official publication, the Society for Vascular Surgery [and] International Society for Cardiovascular Surgery, North American Chapter* 2009;49(1):230-9.
23. (SIGN) SIGN. *Management of chronic venous leg ulcers*: NHS, 2010.
24. Kulkarni SR, Slim FJ, Emerson LG, Davies C, Bulbulia RA, Whyman MR, et al. Effect of foam sclerotherapy on healing and long-term recurrence in chronic venous leg ulcers. *Phlebology* 2012.
25. Pang KH, Bate GR, Darvall KA, Adam DJ, Bradbury AW. Healing and recurrence rates following ultrasound-guided foam sclerotherapy of superficial venous reflux in patients with chronic venous ulceration. *Eur J Vasc Endovasc Surg* 2010;40(6):790-5.
26. Michaels JA, Campbell WB, Brazier JE, Macintyre JB, Palfreyman SJ, Ratcliffe J, et al. Randomised clinical trial, observational study and assessment of cost-effectiveness of the treatment of varicose veins (REACTIV trial). *Health Technol Assess* 2006;10(13):1-196, iii-iv.

Appendix 1: Summary of assessments and follow-up visits

Time point	Estimated duration (mins)	Clinical evaluation[a]	Telephone follow-up[b]	Wound review / tracing	Wound photo	Venous duplex	Randomisation	Consent	Health Questionnaires (EQ-5D, SF-36, AVVQ)
Screening Visit	45	X				X		X*	
Baseline Visit	60-90	X		X	X		X	X**	X
1 month	30		X						
6 weeks	60-90	X		X?	X	X[c]			X
2 months	30		X	X?	X?				
3 months	30		X	X?	X?				
4 months	30		X	X?	X?				
5 months	30		X	X?	X?				
6 months	30		X	X?	X?				X
7 months	30		X	X?	X?				
8 months	30		X	X?	X?				
9 months	30		X	X?	X?				
10 months	30		X	X?	X?				
11 months	30		X	X?	X?				
12 months	30		X	X?	X?				X

a. Demographic details (age, sex, ethnicity), Pregnancy test for woman of child bearing potential. General clinical details (body mass index, ankle brachial pressure index – performed within previous 4 weeks, comorbidities, medication history). Ulcer details (duration, progression, previous ulcer history, size of current ulcer – using photography and planimetry). Details of venous disease (previous deep vein thrombosis, previous venous interventions, pattern of venous reflux on duplex)

b. . Ulcer healing assessment, compression type, AE assessment, Concomitant medications, health resource use

c. Only for those who have early endovenous treatment

*Approached **Taken

?dependant on whether the ulcer has healed tracing and photo will be taken at verification visit and taken weekly for 1 month. Once the ulcer has healed the patient will still be followed up with monthly phone calls.

EVRA

EVRA (Early Venous Reflux Ablation) ulcer trial

A randomised clinical trial to compare early versus delayed endovenous treatment of superficial venous reflux in patients with chronic venous ulceration.

Version 5.0, 06/04/2017

MAIN SPONSOR: Imperial College London (Sponsor Number 13HH0722)

ISRCTN02335796

STUDY COORDINATION CENTRE: Imperial College Trials Unit

NRES reference: 13/SW/0199

IRAS Ref: 131153. UK CRN Study ID: 15078

		Date	Signature
**Authorised by:**	**Name:** **Professor Alun H Davies**	**11/05/2017**	

Revision History

Protocol Version	Date	Amendments
V5.0	06/04/2017	To incorporate the HTA funding extension to the trial to allow for the collection of longer term follow-up during October 2018 and March 2019<br>Amendments to the health economics section to clarify some items which were unclear in the previous version, and update the protocol to reflect new NIHR guidelines.
V4.0	16/03/2016	To correct sample size from 500 participants to 450 participants which was originally calculated erroneously<br>To allow for a reduction in the number of photo verification visits performed if the core lab confirms the ulcer is healed.
V3.0	10/03/2014	Amended in order to display posters, leaflets and disseminate patient information sheets in primary care sites
V2.0	06/01/2014	A clearer definition of ulcer healing is required to clarify that healing cannot be assume if a scab is present.<br>Statistics and Data Analysis' section amended for clarity of per-protocol analyses.<br>Serious adverse event (section 8.2) amended for clarity.<br>Section 5.4 amended to clarify that patients can be offered intervention in the standard care (compression arm) if their ulcer has not healed at 6 months.
V1.0	19/06/2013	N/A – Original Protocol

Study Management Group

Chief Investigator: Professor Alun H Davies

Co-investigators: Mr Manjit S Gohel, Mr Richard Bulbulia, Mr Keith R Poskitt, Professor Andrew Bradbury, Professor Nicky Cullum, Miss Sophie R Renton, Mr I Nyamekye

Statistician: Dr Jane Warwick

Health economist: Dr David Epstein

Study Management: Miss Francine M Heatley

Study Coordination Centre

For general queries, supply of study documentation, and collection of data, please contact:

Study Coordinator: Miss Francine M Heatley

Address: Vascular Surgery Research Group, Room 4E3, 4th Floor East Wing

Charing Cross Hospital, Fulham Palace Road, London W6 8RF

Tel: 020 3311 7371

E-mail: f.heatley@imperial.ac.uk

Web address: www.evrastudy.org

Clinical Queries

Clinical queries should be directed to either the Local PI or the Study Coordinator who will direct the query to the appropriate person

Sponsor

Imperial College London is the main research Sponsor for this study. For further information regarding the sponsorship conditions, please contact the Head of Regulatory Compliance at:

Joint Research Compliance Office

Imperial College London and Imperial College Healthcare NHS Trust

Room 215, Level 2, Medical School Building

Norfolk Place

London, W2 1PG

Tel: 0207 594 1872

This protocol describes the EVRA study and provides information about procedures for entering participants. Every care was taken in its drafting, but corrections or amendments may be necessary. These will be circulated to investigators in the study. Problems relating to this study should be referred, in the first instance, to the Chief Investigator.

This study will adhere to the principles outlined in the NHS Research Governance Framework for Health and Social Care (2nd edition). It will be conducted in compliance with the protocol, UK Clinical Trials Regulations, the Data Protection Act and other regulatory requirements as appropriate.

This project is funded by the National Institute for Health Research HTA (project number 11/129/197). The views and opinions expressed therein are those of the authors and do not necessarily reflect those of the HTA, NIHR, NHS or the Department of Health.

ABBREVIATIONS

AE	Adverse Event
CI	Chief Investigator
CRF	Case Report Form
DMC	Data Monitoring Committee
ICTU	Imperial Clinical Trials Unit
REC	Research Ethics Committee
QA	Quality Assurance
SAE	Serious Adverse Event
SAP	Statistical Analysis Plan
SOP	Standard Operating Procedure
SUSAR	Suspected Unexpected Serious Adverse Reaction
TSC	Trial Steering Committee

1. INTRODUCTION

1.1 BACKGROUND

Chronic leg ulcers are open “sores” on the lower limbs situated between the ankles and knees, which fail to heal within 6 weeks. These ulcers represent a source of great discomfort and social isolation to patients who often complain of associated pain, odour and wound discharge. The time taken for the ulcers to heal means that the condition is also particularly frustrating to health carers involved in their management in hospital and community settings. The underlying cause of leg ulceration in over 70% of cases is lower limb venous dysfunction, sometimes evident as varicose veins but often undetectable by visual examination alone[1]. The estimated overall prevalence of active venous ulceration is as high as 1.5 to 1.8 per 1000 population, increasing to 3.8 per 1000 population in those over 40 years of age[23]. As patients with venous ulceration usually suffer episodes of recurrence between periods when the ulcer remains healed, the number of patients with a high risk of ulceration may actually be 4-5 fold higher[4]. It should also be noted that with an aging and increasingly obese population[5], the incidence and prevalence of venous ulceration are both likely to increase. Treatment of the condition in the UK produces a substantial cost burden estimated at £400-600 million per annum[6].

Venous ulcers are characterised by protracted healing times. Despite some recent advances in the management of patients with venous ulcers, 24 week healing rates in published randomised trials are around 60-65%[78], and the true population healing rates are likely to be significantly lower. Some patients may never heal and those that do heal are at high risk of recurrent ulceration. These poor outcomes are likely to be a reflection of the severe underlying venous dysfunction in this patient group, although inadequate assessment and suboptimal treatment are also likely to be important contributing factors.

1.1.1 Pathophysiology of venous ulceration

The venous circulation of the lower limb has two components, the deep and superficial systems. Blood normally flows from the superficial to the deep veins and is prevented from flowing back down the leg under the influence of gravity by ‘one-way’ valves along the veins. When these valves become incompetent (leaky), the superficial veins usually become dilated and tortuous (varicose) and the resulting sustained high venous and capillary pressures lead to skin inflammation and ulceration (breakdown of skin). The deep veins also have valves, which may also become incompetent, but are not visible on the skin. Duplex ultrasound studies[91011] on patients in leg ulcer clinics suggest that:

- Around 50% of patients with venous leg ulcers have diseased superficial veins alone, with a further 30-40% having a mixture of superficial and deep venous disease. Both of these groups of patients benefit from correction of their

superficial venous reflux, which has been shown to reduce the risk of ulcer recurrence[12].

- A minority (5-10%) of patients with venous ulcers have diseased deep venous systems only, and are not amenable to surgical correction. These patients are usually treated with compression bandaging alone

Ulcer healing strategies are based on efforts to reduce this leakage (reflux) of blood back down the leg and into the skin, as this is considered the most significant cause of high venous pressure in most patients. Longstanding venous hypertension has been shown to cause a number of changes to the microcirculation in the lower leg, which can contribute to the chronic skin changes or eventual ulceration associated with chronic venous disease[13]. Compression bandaging to the leg (which may need to be re-applied 1-4 times per week) counteracts the gravitational force on the blood, in effect temporarily replacing the incompetent valves[14]. Diseased superficial veins can be surgically removed (open varicose vein surgery) or ablated using endovenous interventions (see below) without harming the overall venous function of the leg, theoretically removing a causative factor for recurrence of the ulcer after the compression bandaging has ceased. The deep vein defects are not generally amenable to surgery.

1.1.2 Treatment options for superficial venous reflux

For over a century, the treatment of superficial venous reflux has involved operative ligation and surgical stripping of the vein and avulsion of bulging varicose veins[15]. Until recent years, open surgery has been considered the definitive treatment option for superficial venous reflux. However, the operation usually requires general anaesthesia and patients often suffer discomfort, bruising and significant time off work in the post-operative period. Long-term studies have also identified significant complications of open surgery including nerve damage and recurrence of varicose veins, seen in over 60% of patients at 11 years in one randomised study[16].

In response to this high complication rate and a growing patient desire for less invasive treatments, a range of novel, minimally invasive endovenous treatment options have been developed and have gained in popularity over the last decade. Interventions such as ultrasound guided foam sclerotherapy (UGFS)[17], endovenous laser (EVLA)[18] or radiofrequency ablation (RFA)[19] can be performed using local anaesthesia in an outpatient setting. These treatments involve cannulation of the vein to be treated (usually under ultrasound guidance) and obliteration of the venous channel by either chemical ablation (using foam sclerosant), or thermal ablation (using a laser or radiofrequency fibre). Numerous randomised studies have demonstrated that endovenous modalities are, at worst, comparable to open surgery in terms of recurrence (and likely to be better), but clearly superior in terms of pain, bruising and other early complications[20-22]. Each of the different endovenous modalities has advantages and potential disadvantages, although all are less invasive than traditional open surgery. This is of particular relevance to patients with

chronic venous ulceration, who are often elderly, have extensive co-morbidities and may be reluctant to undergo surgical procedures involving general anaesthesia. Endovenous techniques can also be performed without discontinuing anti-coagulation therapy, which is increasingly prescribed in this patient population.

1.1.3 Summary of current research

The most significant study of superficial venous intervention in patients with venous ulceration is the ESCHAR study (Barwell, Poskitt; Lancet 2004 & Gohel, Poskitt; BMJ 2007)[7 12]. The study aimed to evaluate the role of traditional superficial venous surgery in reducing ulcer recurrence in patients with open or recently healed venous ulcers. Following prospective observational studies to inform power calculations, a total of 500 patients were randomised to compression therapy alone or compression with open surgery for superficial venous reflux. The group randomised to surgical treatment had significantly lower venous ulcer recurrence rates at 4 years (Figure 1).

Analysis stratified by pattern of venous reflux demonstrated that this clinical benefit was present for patients with isolated superficial venous reflux and patients with superficial and segmental deep reflux. This clearly indicated that the majority of patients with chronic venous ulceration could benefit from superficial venous intervention. As a result, the current optimal management of patients with venous ulceration includes the treatment of refluxing superficial veins to reduce the risk of ulcer recurrence[23].

Analysis of ulcer healing within the ESCHAR trial demonstrated that there was no significant improvement in ulcer healing rates for the group randomised to compression plus surgery (Figure 2). This finding has led many to conclude that treatment of venous reflux does not have a role in patients with open ulcers.

Figure 1. ESCHAR trial – ulcer recurrence

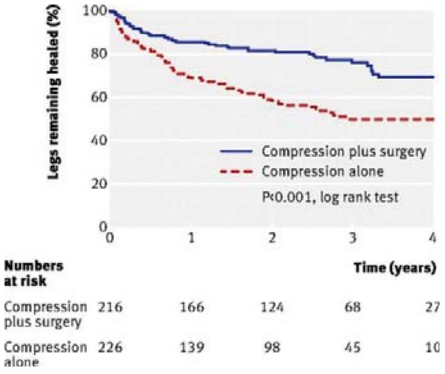

Figure 2. ESCHAR trial – ulcer healing

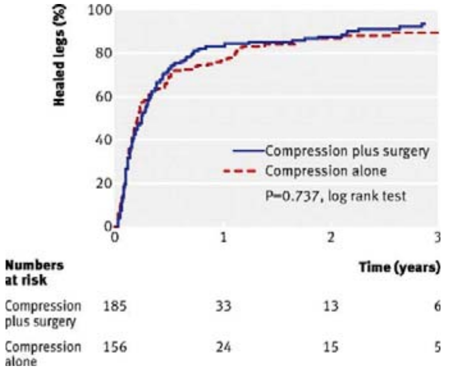

However, the ESCHAR study was designed and powered to assess ulcer recurrence rather than healing, and the statistical power of this trial was further weakened by a high cross-over rate, as around a quarter of patients randomised to surgery subsequently refused to have an operation. This highlights the need for a minimally invasive superficial venous treatment modality in this patient group. In addition, the

median time to treatment within the study was around 2 months, by which time smaller ulcers may have already healed with compression bandaging, and, in many cases, the surgical procedures used were suboptimal when judged by current standards. Consequently, it is plausible that the benefits of treating superficial venous reflux were underestimated in this study, particularly for the assessment of ulcer healing.

In a smaller Dutch randomised trial, 170 patients (200 legs) were randomised to compression alone or compression with surgical treatment of superficial reflux (including subfascial endoscopic perforator surgery – SEPS)[8]. Although results did not reach statistical significance, there was a clear trend towards improved ulcer healing rates and greater ulcer free time in the group randomised to surgery.

Despite the widespread acceptance of endovenous modalities, few prospective studies have been published reporting outcomes after endovenous treatment in patients with leg ulcers. In a prospective study of 186 patients with leg ulceration treated with UGFS, the ulcer healing rate was over 70% and the patient acceptability of treatment was excellent (Poskitt et al)[24]. In a further study of foam sclerotherapy in 130 patients, a healing rate of 82% was achieved (Bradbury et al)[25]. Whilst these small non-randomised studies lend support to our hypothesis that early intervention to correct superficial venous reflux will promote ulcer healing, a large randomised trial is required to provide reliable evidence and thus change practice.

1.2 RATIONALE FOR CURRENT STUDY

Whilst the management of patients with venous ulcers has evolved in recent years and ulcer healing and recurrence rates have shown some improvement, we believe that there is a strong argument in favour of this study at this time for the following reasons:

- The prevalence of venous ulceration is likely to increase, particularly with an aging and increasingly obese population. In view of the significant financial and psychosocial costs of venous ulceration, it is imperative that the optimal treatment strategies are identified.
- Despite numerous studies of topical ulcer treatments, the only treatment shown to improve venous ulcer healing is compression bandaging. Compression supports the venous circulation, but is poorly tolerated by some patients and does not address the underlying problem of venous reflux. The intervention in this proposal involves treating the underlying anatomical venous disorder using effective, minimally invasive endovenous interventions and offers a logical, deliverable and long-term approach to reducing venous hypertension.
- The treatment of superficial venous reflux has been transformed in recent years through the widespread use of minimally invasive, endovenous interventions, which patients find more acceptable than traditional open surgery.

- Ablation of superficial reflux should be considered in all patients with leg ulcers and superficial venous reflux, but if early intervention is associated with moderate improvements in ulcer healing compared to deferred intervention (i.e. post-healing), significant cost savings could be realised.
- Patients find venous leg ulcers painful, distressing and a significant inhibition to normal, independent life. Interventions to reduce the time to healing could reduce patient distress and significantly improve quality of life.

Therefore, we believe that there is a cogent argument for conducting this trial at this time. Non-randomised studies suggest that outcomes may be improved by treating underlying superficial reflux using the latest technologies, but there is no robust evidence to support early intervention. The research team has a strong track record in relevant research areas and includes clinicians and researchers who successfully completed the landmark clinical trial on which this proposal is based (ESCHAR trial), and numerous other high impact clinical trials evaluating treatments in venous ulceration.

2. OBJECTIVES

2.1 PRIMARY OBJECTIVE

To determine the clinical and cost effectiveness of early endovenous treatment of superficial venous reflux in addition to standard care compared to standard care alone in patients with chronic venous ulceration.

2.2 SECONDARY OBJECTIVES

To investigate:

- The ulcer free time to 1 year and with the extension, up to 5 years (median of approximately 3.7 years)
- The technical success of endovenous interventions

3. PARTICIPANT ENTRY

3.1 PRE-REGISTRATION EVALUATIONS

Prior to commencing, information will be disseminated to GP practices in each recruiting region and meetings will be arranged with key community nursing staff and at leg ulcer clinics to promote the trial. Patients will be referred to secondary care as part of the standard care pathway as per the July 2013 NICE Guidelines. To aid recruitment, selected Primary Care trusts not currently involved in the trial will be set-up as Patient Identification Centres (PIC sites) displaying posters, leaflets and disseminating patient information sheets to patients. Selected Primary Care trusts involved in follow-up of the trial (research sites) will also aid recruitment by displaying posters, leaflets and disseminating patient information sheets to patients. Patients will still need to be referred to the secondary care recruiting sites to be consented and randomised into the trial.

At the referral visit patients will be given an appropriate time period to consider participation (at least 24 hours). Written consent will be obtained from those patients who agree to participate and randomization will be performed using the online service. For patients randomised to endovenous ablation of superficial venous reflux, a date for intervention will be booked as soon as possible (i.e. within 2 weeks). At each recruiting centre, an online log of all screened patients will be kept using the InForm system. Basic demographic data and reasons for non-eligibility will be recorded. Whilst participant baseline characteristics may vary slightly across recruiting sites, randomised treatment allocation will allow reliable assessment of the effects of early versus delayed endovenous ablation in ulcer healing.

3.2 INCLUSION CRITERIA

- Current leg ulceration of greater than 6 weeks, but less than 6 months duration
- Able to give informed consent to participate in the study after reading the patient information documentation
- Patient age > 18 years
- Ankle Brachial Pressure Index (ABPI) $\geq$ 0.8
- Superficial venous disease on colour duplex assessment deemed to be significant enough to warrant ablation by the treating clinician (either primary or recurrent venous reflux)

Patients who cannot speak / understand English will be eligible for inclusion and informed consent will be obtained with assistance from translation services as per standard clinical practice. In view of the lack of cross-cultural validation for quality of life tools, only healing outcome data will be collected.

3.3 EXCLUSION CRITERIA

- Presence of deep venous occlusive disease or other conditions precluding superficial venous intervention (at the discretion of local research team)

- Patients who are unable to tolerate any multilayer compression bandaging / stockings will be excluded. However, concordance with compression therapy can be variable for patients at different times. Patients who are generally compliant with compression, but unable to tolerate the bandages for short periods will still be eligible to inclusion. A period of non-compliance with compression bandages will not be considered a protocol violation, but a normal variation within the spectrum of ‘standard therapy’.
- Inability of the patient to receive prompt endovenous intervention by recruiting centre
- Pregnancy (female participants of reproductive age will be eligible for inclusion in the study, subject to a negative pregnancy test prior to randomisation)
- Leg ulcer of non-venous aetiology (as assessed by responsible clinician)
- If patient is deemed to require skin grafting they cannot be included

4. STUDY DESIGN

The EVRA ulcer trial is a pragmatic; multicentre randomised clinical trial with participants randomised1:1 to either:

1. ‘Standard’ therapy consisting of multilayer elastic compression bandaging/ stockings with deferred treatment of superficial reflux (usually once the ulcer has healed)
2. Early endovenous treatment of superficial venous reflux(within 2 weeks) in addition to standard therapy

The study design is summarised in Figure 3 below.

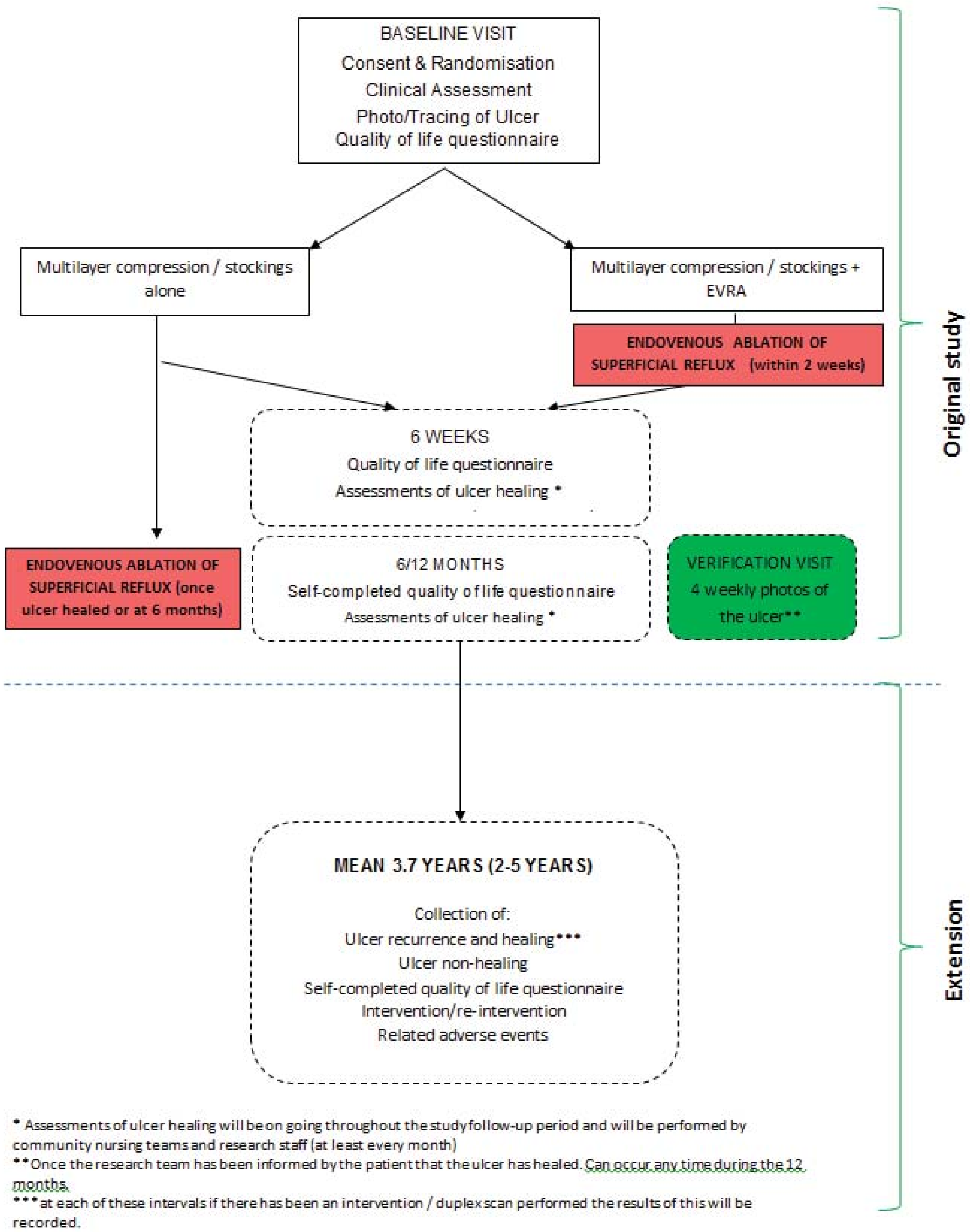

* Assessments of ulcer healing will be on going throughout the study follow-up period and will be performed by community nursing teams and research staff (at least every month)
**Once the research team has been informed by the patient that the ulcer has healed. Can occur any time during the 12 months.
***at each of these intervals if there has been an intervention / duplex scan performed the results of this will be recorded.

4.1 PATIENT RANDOMIZATION

The normal clinical team will make initial contact with potentially eligible patients at the referral visit.

Those who consent will be registered on the InForm ITM (Integrated Trial Management) System, a web-based data entry system, which is maintained by ICTU, and their eligibility for the study confirmed. A randomization list will be loaded onto the InForm system for each centre (as stratification will be by centre) before recruitment commences, having been prepared in advance by a statistician who is independent of the study. Each potential participant, if confirmed to be eligible, will be assigned the next available entry in the appropriate randomization list (i.e. without foreknowledge). Thereafter, treatment allocation will not be blinded (with the exception of assessment of ulcer healing – see 4.3.1). For patients with bilateral venous ulceration, the worst leg (according to the patient) will be designated the 'reference leg'. Interventions may be performed on both legs, if deemed appropriate by the responsible clinician.

4.2 STUDY SETTING

Eligible patients with chronic venous ulcers will initially be recruited from the following centres:

1. Imperial College Healthcare NHS Trust (PI: Professor AH Davies)
2. Cambridge University Hospitals NHS Foundation Trust (PI: Mr MS Gohel)
3. Worcestershire Acute Hospitals NHS Trust (PI: Mr I Nyamekye)
4. North West London Hospitals NHS Trust (PI: Miss SR Renton)
5. Gloucestershire Hospitals NHS Foundation Trust (PI: Mr KR Poskitt)
6. Heart of England NHS Trust (PI: Professor A Bradbury)
7. University Hospital Birmingham NHS Trust (PI: Mr Rajiv Vohra)
8. City and Sandwell NHS Trust (PI: Miss Rachel Sam)
9. The Dudley Group NHS Trust (PI: Mr Andrew Garnham)
10. The Royal Wolverhampton Hospitals NHS Trust (PI: Mr Andrew Garnham)
11. York Hospitals NHS Foundation Trust
12. Hull & East Yorkshire Hospitals NHS Trust
13. The Royal Bournemouth and Christchurch Hospitals NHS Foundation Trust
14. Frimley Park Hospital NHS Foundation Trust
15. Plymouth Hospitals NHS Trust

16. Bradford Teaching Hospitals NHS Foundation Trust
17. Salisbury NHS Foundation Trust
18. Leeds Teaching Hospitals NHS Trust
19. Sheffield Teaching Hospitals NHS Foundation Trust
20. Taunton and Somerset NHS Foundation Trust

As per section 3.1 Primary Care Trusts will be set-up as either PIC sites or research sites aiding recruitment by displaying posters, leaflets and disseminating patient information sheets. Patients will still need to be referred to the secondary care recruiting sites to be randomised into the trial.

4.3STUDY OUTCOME MEASURES

4.3.1 Primary outcome measure

The primary outcome measure will be time to ulcer healing (from date of randomization to date of healing). For the purposes of this study, ulcer healing is defined as complete re-epithelialisation of all ulceration on the randomised (reference) leg in the absence of a scab (eschar) with no dressing required. Community or hospital healthcare staff, depending on the local model of care, will perform assessment of ulcer healing.

Data on the status of the reference leg will be collected throughout the study by research staff scrutinising community medical / nursing records and contacting the patient / community nursing teams by telephone(on a monthly basis at least).

If either the community nursing / medical staff or the patient believe that ulcer healing has been achieved, they will be asked to contact the local research centre immediately. This notification of possible ulcer healing will constitute a ‘trigger’ forth research staff at the recruiting centre to arrange an urgent verification assessment by a member of the healthcare team (within 1 week).

Verification will be by clinical assessment and digital photography, to be repeated weekly for 4 weeks, unless otherwise agreed by the trial manager. The digital images will be evaluated by two blinded expert assessors in order to ascertain the date of healing, which will be considered the primary healing end-point. For the purposes of the trial healing will be defined as the complete re-epithelialisation of the ulcerated (reference) leg in the absence of a scab (eschar) with no dressing required. Healing cannot be assumed if a scab present.

Disagreements will be resolved through discussion with involvement of a third blinded expert reviewer if necessary. This approach will be applied to patients in both treatment arms and is consistent with the methods utilized in other large HTA funded leg ulcer trials (e.g. VenUS IV). Legs deemed to have an open ulcer on clinical assessment would continue within the study. If healing is confirmed by clinical and

blinded photograph assessments at the first verification visit, the date of healing notification (by patient or community nurse) will be taken as the date of ulcer healing.

4.3.2 Secondary outcome measures

A number of secondary outcome measures will be evaluated in the EVRA study:

1. Ulcer Healing Rate: Healing rate will be reported at 24 weeks in addition to time to ulcer healing to allow comparison with other published studies.
2. Ulcer recurrence / Ulcer Free Time: Will be calculated up to 1 year for each study arm and with the extension, up to 5 years (median approximately 3.7 years). This will allow a very practical and easily understood assessment of the clinical difference between the 2 arms of the study. This will also allow comparison with other studies that have reported this outcome. In order to facilitate accurate calculation of reoccurrence / ulcer free time, clinical follow up will be continued after ulcer healing up to 1 year after randomisation.
3. Quality Of Life (QoL): Disease specific (AVVQ) and generic (EQ5D & SF36) quality of life assessments will be compared at 6 weeks post randomisation, 6 months, 12 months and at one time point between October 2018 and March 2019. The 6-week questionnaire will be given to the patient at the follow-up appointment, whereas other QoL questionnaires will be sent to the patient or completed by the patient via telephone. AVVQ is the most widely utilised disease specific QoL tool in venous disease and has been extensively validated. A score out of 100 points is calculated, with a higher score indicating more severe QoL impairment. Changes in QoL scores will offer a comparison with other studies and, in the standard treatment arm, will allow an assessment of the natural history of venous ulceration treated with compression.
4. Health Economic Assessment: A within-RCT cost effectiveness analysis will be carried out based on the data collected in the trial, Resource use items in hospital and community care related to the treatment of venous ulceration or complications will be recorded for each patient at each follow-up. Resource use will be multiplied by UK unit costs obtained from published literature, HRG costs, and manufacturers' list prices to calculate overall costs. A standard tariff will be applied for each bandage change. Utilities (QALYs) will be calculated from the EQ-5D questionnaire administered to patients at baseline, 6 weeks, 6 months, 12 months and at one time point between October 2018 and March 2019. The extent of missing data will be assessed and appropriate methods to handle missing data will be applied if necessary. The incremental cost-effectiveness ratio will be calculated and compared to current UK decision making thresholds. Discounting will be applied at the standard rate. Sensitivity analysis will be carried out to test the robustness of results to alternative assumptions (for example, about missing data, or using per-protocol estimates of treatment effect) or alternative data (for example, about unit costs). Probabilistic sensitivity analysis will be carried out using bootstrapping. A decision model will also be constructed to take account of

outcomes (such as recurrence or healing) that might occur beyond time horizon of the RCT, or to take account of other relevant comparators in this patient group.

5. Other Markers of Clinical Success: The Venous Clinical Severity Score (VCSS) will be assessed at 6 weeks. In addition, the incidence of complications related to the endovenous intervention as well as the presence of residual / recurrent varicose veins will also be assessed at 6 weeks in the early arm.

4.4 DURATION OF FOLLOW-UP

In the original study, participants were to be followed-up until either:

1. 1 year post-randomization
2. Patient choice to withdraw from the study. Patients who no longer wish to complete quality of life questionnaires will be asked if they would object to the use of healing status data (to contribute to the primary outcome)
3. Death

In order to allow assessment of ulcer free time to 1 year, patients with healed ulcers were to be evaluated using telephone follow-up (performed by staff at the recruiting centre) on a monthly basis until 1 year. The aim of the telephone follow-up was to confirm that the ulcer remains healed, or in cases of ulcer recurrence, to ascertain the date of recurrence and of subsequent healing.

In December 2016 The HTA approved an extension to the trial follow-up allowing the collection of follow-up data for all patients who have not withdrawn consent to the trial. Data collection will commence in October 2018, allowing a median follow-up period of up to 5 years (median approximately 3.7 years) to be obtained (further details given in section 6.6).

4.5 STUDY DURATION

The EVRA study will take 70 months to complete. The revised study timetable is summarised in Figure 4.

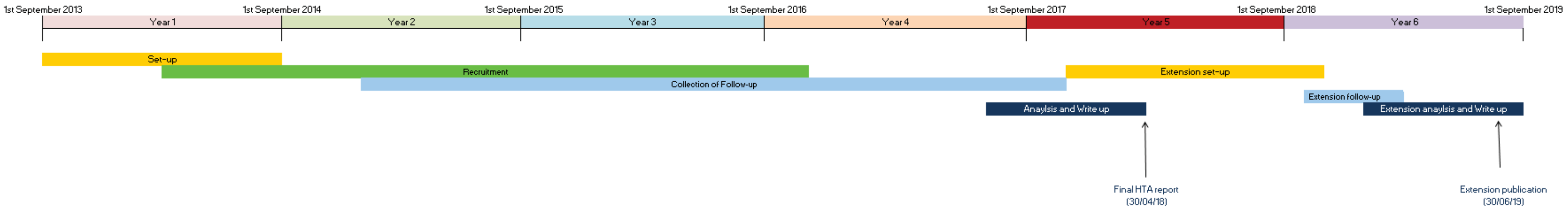

Figure 4. EVRA Study timeline

5. DETAILS OF INTERVENTIONS

5.1 VARIATIONS IN ENDOVENOUS INTERVENTIONS

A wide range of endovenous treatment modalities are now available and in widespread use for the ablation of superficial venous reflux. These include:

- Endovenous thermal ablation using laser or radiofrequency
- Ultrasound guided foam sclerotherapy (UGFS)
- Other endovenous interventions such as mechanochemical ablation, steam ablation and glue
- Any combination of the above treatments

In addition to the different modalities in use, the treatment strategy may also vary between institutions and between individual clinicians within the same department. Variations may occur in:

- Site of vein cannulation (and therefore the length of vein ablated)
- Location of treatment ('office' or clinic based versus operating theatre)
- Treatment strategy for sub-ulcer venous plexus (to ablate or not)
- The treatment of visible varicose veins (no treatment, UGFS or surgical avulsion) and the timing of any intervention

5.2 STANDARDISATION OF INTERVENTIONS IN EVRA STUDY

With the lack of consensus on a single, optimal endovenous treatment strategy for superficial reflux in patients with leg ulceration, perfect standardisation of interventions will be impossible. All endovenous interventions should be performed as deemed to be 'optimal' by the treating clinician for each individual patient, with the following stipulations:

1. The endovenous strategy must include ablation of the main truncal venous reflux
2. Truncal venous reflux should be treated to the lowest point of incompetence, where possible
3. Significant (as deemed by the treating clinician) residual / recurrent superficial reflux on the 6 week duplex scan, should be ablated
4. Patients should continue with multilayer compression / stockings immediately after treatment

5.3 STANDARDISATION OF COMPRESSION

Patients will receive the standard compression used in the individual centres prior to ulcer healing following randomisation (this will include four layer bandaging, three layer bandaging, European short stretch, stockings). Post healing the patients will be given compression hosiery in line with local policy.

5.4 FURTHER TREATMENT FOR STANDARD CARE (COMPRESSION ALONE) ARM

Patients randomised to multilayer compression / stockings alone can be offered endovenous treatment of superficial reflux once healing has been confirmed or at 6 month post randomisation (see 4.3.1). Endovenous ablation should be performed as per standard practice in the treating centre and details of this will be recorded. Endovenous intervention may also be offered if there is clinical deterioration in the active leg ulcer and it is clinically felt that the patient may benefit from early intervention. This will be recorded on the electronic case report form.

6. ASSESSMENT AND FOLLOW-UP

6.1 PATIENT IDENTIFICATION

Patients will be referred to secondary care for evaluation of the management of their leg ulcer as part of the standard pathway of care.

6.2 REFERRAL VISIT

At the initial visit the patient will be evaluated by clinical assessment and colour duplex examination, which is part of the normal investigation of a patient with leg ulceration. Dependant on the results of these tests, the patient will be asked if they would consider taking part in the trial and approached for consent. The patient will be given a minimum of 24 hours to consider the trial and if willing to participate will return to the leg ulcer clinic to give consent and undergo a baseline visit.

6.3 BASELINE VISIT

Patients will undergo detailed clinical assessment by the research nurse as part of the baseline evaluation (see *Appendix 1*). Recorded assessments will include:

- Demographic details (age, sex, ethnicity)
- Pregnancy test for woman of child bearing potential
- General clinical details (body mass index, ankle brachial pressure index – performed within previous 4 weeks, comorbidities, medication history)
- Ulcer details (duration, progression, previous ulcer history, size of current ulcer – using photography and planimetry)
- Details of venous disease (previous deep vein thrombosis, previous venous interventions, pattern of venous reflux on duplex)

Additional assessments will include:

- Assessment of Clinical, Etiologic, Anatomic, Pathophysiological (CEAP) score
- Assessment of venous clinical severity score (VCSS)
- Disease specific (Aberdeen varicose vein questionnaire – AVVQ) and generic (EuroQoL 5D – EQ5D & short form (SF) 36) quality of life assessments

At this visit, eligible and consenting patients will be randomised into the trial.

6.4 FOLLOW-UP ASSESSMENTS

Randomised patients will undergo routine leg ulcer care in community or hospital (or both) settings, in accordance with the local standard. This will equate to wound reviews and dressing changes ranging between once and 4 times per week (depending on the ulcer). The exact nature of dressings and date of dressing change will be documented by the completion of patient diaries. This will allow an accurate record of the dressing types used and will be collected and verified by the research nurse.

In addition, the following assessments will be conducted:

6.4.1 6-week clinic visit

- Clinical assessment
- In the compression plus early venous reflux ablation group, venous duplex scanning will be performed at 6 weeks post-randomization to verify anatomical treatment success. Depending on the results of the scan, the decision to perform further superficial venous interventions will be left to the discretion of the responsible clinical staff. Irrespective of the number and timing of venous interventions, all analyses will be performed on intention to treat.
- Wound tracing and photo
- Assessments of disease specific and generic quality of life (AVVQ, EQ5D & SF36) by means of self-completed questionnaire

6.4.2 Further follow-up

- Assessments of disease specific and generic quality of life (AVVQ, EQ5D & SF36) by means of self-completed questionnaire at 6 months and 12 months post-randomization (sent to the patient).
- The research team will perform monthly telephone evaluation of the patient and access the community notes or telephone the community nurses in order to collect and verify the data collected.
- Once the research team has been informed that the ulcer has healed the patient will undergo an urgent verification visit

6.5 URGENT VERIFICATION VISIT

- A member of the local research team will perform the four verification visits to confirm healing. Photographs will be taken and send to the Trials Unit for independent verification. In order to minimise inconvenience to the participants, once core labs confirms healing it is not necessary for the research team to perform further verification visits. Please note all four photos should be taken unless the trial manager confirms otherwise.

6.6 LONGER TERM FOLLOW-UP

For each randomised patient a single telephone assessment will be performed between October 2018 and March 2019 to collect:

- Details of any further ulcer recurrence and healing events
- Assessment of ulcer related healthcare attendances and costs
- Details of all further venous interventions performed and any associated adverse events
- Assessments of disease specific and generic quality of life (AVVQ, EQ5D & SF36) by means of self-completed questionnaire completed over the telephone (or via post)

The research teams will also evaluate healthcare records to:

- verify ulcer recurrence and healing events
- obtain specific details about venous investigations and interventions performed including delays to intervention.

No anatomical assessments of long-term treatment success are planned; however, additional treatments will be recorded and included in the health-economic evaluations.

7. STATISTICS AND DATA ANALYSIS

Data and all appropriate documentation will be stored for a minimum of 10 years after the completion of the study, including the follow-up period in accordance with the Imperial College JCRO Archiving Study Documents SOP.

7.1 SAMPLE SIZE CALCULATION

The sample size calculation for this study was based on the primary outcome of ulcer healing. The ESCHAR trial was a similar randomised study, which published the final results in 2007 (see 1.1.3). A total of 500 patients with open or recently healed venous ulcers were randomised to standard therapy alone or standard therapy plus open surgery for superficial venous reflux. The study was powered and designed to evaluate differences in ulcer recurrence (rather than healing). Consequently, the median time from randomization to treatment delivery was over 7 weeks. Nevertheless, the 24-week healing rate in patients randomised to standard treatment (compression alone) was approximately 60%. Two recent prospective studies evaluating the early treatment of superficial venous reflux suggested that the 24-week healing rate may be as high as 82%[24 25].

In order to calculate a sample size for this study, a benefit associated with early treatment is estimated at around 15%. Assuming the 24-week healing rate in the standard arm is 60%, to identify a difference in 24-week healing rates of 15% between the two groups (60% vs 75%) with 90% power and allowing for 10% dropout the study will therefore require 416 subjects (208 in each arm, 254 healed leg ulcers in total). To incorporate further allowances for protocol violations and unexpected dropouts, the target sample size will be 450 patients.

Assuming a 15% drop out rate for the study and that 90% of primary ulcers eventually heal, it is estimated that >340 of the recruited 450 patients will be eligible for inclusion in the ulcer recurrence analysis. With this number of participants, and allowing for the healing rates (of the index ulcer prior to entry into this analysis of ulcer recurrence) to differ by up to 20% between the two study arms, the study extension will have at least 80% power to detect a difference of 15% or more in ulcer recurrence rates between the two arms at the 5% significance level.

7.2 PLANNED ANALYSES

No formal interim analyses are planned. Informal interim analyses will be performed if requested by the Data Monitoring Committee (DMC) but findings will be made available to member of the DMC only. Basic descriptive methods will be used to present the data on study participants, trial conduct, clinical outcomes and safety (in total and for each study group separately). The primary outcome will be time to complete healing. We will test the hypothesis that there is no difference in time to complete healing between the control and intervention groups using a log-rank test

(two-tailed, 5% significance level). Kaplan-Meier survival curves will also be presented and we will perform a subsidiary analysis investigating the effect of study centre, participant age, ulcer size and chronicity on time to complete healing using Cox regression. To adjust for potential surgeon and centre effects, surgeon and centre will be included in the Cox regression analysis as random effects. All analyses will be on an intention-to-treat basis. If there is substantial cross-over, per-protocol analyses may be explored for sensitivity analyses. Safety and tolerability data will be presented by the two arms on an intention-to-treat basis. The statistical analysis plan (SAP) for the original trial (follow up to 1 year) will be finalised prior to the final analysis. An additional SAP for the extension follow-up (up to 5 years) will be finalised before the analysis of extension data.

7.3 MISSING, UNUSED AND SPURIOUS DATA

There will be no data imputation for missing data in the primary endpoint (time to healing) and the secondary endpoint of ulcer free time. Any imputation methods used may be proposed for purposes of sensitivity analysis for other secondary outcomes, including ulcer healing rate, QoL and markers of clinical success. Imputation methods will be fully documented in the SAP.

7.4 DEVIATIONS FROM THE STATISTICAL ANALYSIS PLAN

Any deviation(s) from the final statistical analysis plan in the final analysis will be described and justification given in the final report.

7.5 HEALTH ECONOMIC ANALYSIS

The economic evaluation will be based on both a modelling exercise and a patient level in-trial analysis. The main analyses will be performed from the perspective of the NHS and Personal Social Services. Secondary analyses will be performed from a societal perspective. The price year will be 2017-18. Discounting will be applied according to UK Government guidelines. The study will be reported according to current guidelines for economic evaluation (CHEERS).

The within-trial analysis will compare early versus delayed endovenous treatment of superficial venous reflux in patients with chronic venous ulceration, within the time-horizon of the extended trial. Data will be collected by case note review and questionnaires completed at baseline, 6 weeks, 6 months,12 months and at a single time point during October 2018 and March 2019. Resource use items in hospital and community care related to the treatment of venous ulceration, adverse events or complications will be recorded for each patient at each follow-up. Resource use will be multiplied by UK unit costs obtained from published literature, Healthcare Resource Groups (HRG) costs, and manufacturers' list prices to calculate overall

costs. A standard tariff will be applied for each bandage change. Utilities (QALYs) will be calculated from the EQ-5D questionnaire administered to patients at each follow-up. The extent of missing data will be assessed and appropriate methods to handle missing data will be applied. The incremental cost-effectiveness ratio will be calculated and compared to current UK decision making thresholds. Sensitivity analysis will be carried out to test the robustness of results to alternative assumptions (for example, about missing data, or using per-protocol estimates of treatment effect) or alternative data (for example, about unit costs). Probabilistic sensitivity analysis will be carried out using bootstrapping.

A decision model will also be constructed to take into account outcomes that might be expected to occur beyond the timeframe of the RCT (e.g. recurrence, healing), the results of other RCTs that have assessed early or delayed endovascular therapy for treating venous ulcers, or any relevant comparators that are not considered in the RCT (e.g. surgery, bandaging only). The health states used in the model will be based on the natural history of chronic venous ulcers, to be obtained from the trial, from the literature and from expert opinion. . The inputs for the model will be the transition rates for moving from one state to another, the relative risks for each treatment compared with usual care, and the costs and HRQOL associated with each health state. Use of secondary and primary care patient resource use and EQ-5D responses associated with health states will be estimated mainly from the trial. Sensitivity analyses will be carried out to test the robustness of the model results to alternative assumptions and alternative data. Probabilistic sensitivity analysis will be carried out using Monte-Carlo simulation.

7.6 LOSSES TO FOLLOW-UP AND PROTOCOL VIOLATIONS

The primary assessment involves intention-to-treat analysis. Therefore, strenuous efforts will be made to ensure that only patients willing to undergo either immediate or delayed superficial venous ablation and compression bandaging are randomised. Monthly reports of protocol violations will be provided by local sites to the trial coordinators, who reserve the right to suspend or exclude sites in the event of wilful protocol violations. Similarly, efforts will be made to obtain complete follow-up for all randomised participants (irrespective of whether or not they underwent allocated treatment). For those participants unable or unwilling to attend follow-up appointments, home-visits or follow-up by community nurses may be considered.

A high rate of protocol violation was seen in previous trials of venous ulceration (including the ESCHAR trial). This is likely to reflect the reluctance and apprehension of elderly patients to undergo surgical interventions involving general anaesthesia. The modern management of superficial venous disease involves a range of minimally invasive, endovenous modalities that can be performed using local or no anaesthesia. Procedures are performed on an outpatient basis and can be

completed in around 30 minutes. Published studies of endovenous interventions have demonstrated excellent patient satisfaction and few treatment refusals. Due to the published evidence and extensive personal experience among the research team, the rate of participation should be higher and rate of protocol violations lower than previous studies.

The following will be recorded as protocol deviations:

1) Patients randomised to multilayer compression / stockings plus early venous reflux ablation, who receive endovenous intervention more than two weeks from randomization.
2) Patients who are non-compliant with compression bandaging, defined as use <75% of the prescribed duration.
3) Patients randomised to compression bandaging alone who undergo endovenous ablation prior to verified healing.

8. ADVERSE EVENTS

8.1 REPORTING PROCEDURES

During the first 12 months all serious adverse events and all intervention-related adverse events should be reported. Any serious adverse events reported at the October 2018 to March 2019 follow-up time point should be reviewed the Principal Investigator to assess whether they are related to the treatment pathway and only related events should be reported to the sponsor via INFORM. Depending on the nature of the event the reporting procedures below should be followed. Any questions concerning adverse event reporting should be directed to the Chief Investigator in the first instance.

8.2 RELATED ADVERSE EVENTS

Patients randomised to early venous intervention have the potential risks of treatment. Competent, experienced medical staff will perform all procedures and every effort will be made to prevent adverse effects. The adverse events listed below are expected to be related to the endovenous interventions used in the trial and should be reported. **Please note this is not an exhaustive list, if you suspect an event is related to treatment please contact the Trials Unit.**

Systemic

- **allergic reaction req. local / no treatment**
- **migraine**
- **visual disturbance**
- **fainting**
- **Cough / chest tightness**
- **Systemic infection**
- **PE**
- **TIA**
- **Stroke**

Local

- **Bleeding requiring intervention**
- **Blistering of skin**
- **Pressure damage**
- **Nerve damage**
- **DVT**
- **Hematoma**
- **Patient reported parathesia**
- **Pigmentation of skin**
- **Superficial thrombophlebitis**
- **New ulcer**
- **Deterioration of ulcer**
- **Wound infection**

8.3 NON SERIOUS ADVERSE EVENTS

All such events, which are judged by the local PI to be related to the interventions, whether expected or not, should be recorded in InForm

8.4 SERIOUS ADVERSE EVENTS

In addition to clinical assessments, patients will be contacted on a monthly basis by telephone for 12 months to identify any additional treatments, admissions or other complications related to their leg ulceration. Unrelated serious adverse events will also be recorded and reported in accordance with the Good Clinical Practice guidance up to 12 months. Serious adverse events (SAE) are defined as those adverse events that: result in death; are life-threatening; require in-patient hospitalisation or prolongation of existing hospitalisation; result in persistent or significant disability or incapacity; result in congenital anomaly or birth defect; are cancer; or are other important medical events in the opinion of the responsible investigator (i.e. not life threatening or resulting in hospitalisation, but may jeopardise the participant or require intervention to prevent one or more of the outcomes described previously).

All SAEs reported by participants at (or between) each follow-up visit will be recorded by local researchers and entered into InForm within 24 hours of the researcher becoming aware of the event.

All SAEs will be reported by the trial manager to the Sponsor and Chair of the Data Monitoring Committee. Related and unexpected SAEs will also be reported to the relevant Ethics Committee.

In the event that InForm is not accessible notify the Trial Manager, Francine Heatley:

Tel: 0203 311 7371 (Mon to Fri 09.00 – 17.00)

Email: EVRAtrial@imperial.ac.uk

9. REGULATORY ISSUES

9.1 ETHICS APPROVAL

After approval from the Research Ethics Committee, the study must be submitted for Site Specific Assessment (SSA) at each participating NHS Trust. The Chief Investigator will require a copy of the Trust R&D approval letter before accepting participants into the study. The study will be conducted in accordance with the recommendations for physicians involved in research on human subjects adopted by the 18th World Medical Assembly, Helsinki 1964 and later revisions.

9.2 CONSENT

Consent to enter the study must be sought from each participant only after a full explanation has been given, an information leaflet offered and time allowed for consideration. Signed participant consent should be obtained. The right of the participant to refuse to participate without giving reasons must be respected. After the participant has entered the study the clinician remains free to give alternative treatment to that specified in the protocol at any stage if he/she feels it is in the participant's best interest, but the reasons for doing so should be recorded. In these cases the participants remain within the study for the purposes of follow-up and data analysis. All participants are free to withdraw at any time from the protocol treatment without giving reasons and without prejudicing further treatment. Patients will not be specifically reconsented for the collection data in October 2018 as they already consented for the collection of longer term data at the outset. Patients will be asked, however at the telephone contact if they wish to continue in the study.

9.3 CONFIDENTIALITY

The Chief Investigator will preserve the confidentiality of participants taking part in the study and is registered under the Data Protection Act.

9.4 INDEMNITY

Imperial College London holds negligent harm and non-negligent harm insurance policies, which apply to this study.

9.5 SPONSOR

Imperial College London will act as the main Sponsor for this study. Delegated responsibilities will be assigned to the NHS trusts taking part in this study.

9.6 FUNDING

This project is funded by the National Institute for Health Research HTA (project number 11/129/197).

9.7 QUALITY ASSURANCE AND CONTROL

The study may be subject to inspection and audit by Imperial College London under their remit as sponsor and other regulatory bodies to ensure adherence to GCP and the NHS Research Governance Framework for Health and Social Care (2nd edition). Quality Control will be performed according to the requirements of the Risk Assessment performed by ICTU. The study may be audited by a Quality Assurance representative of the Sponsor. All necessary data and documents will be made available for inspection.

10. STUDY MANAGEMENT

The study will be coordinated by a trial manager based at ICTU reporting to the Clinical Coordinators (MG and RB) and the Chief Investigator (AD). The Clinical Coordinators will liaise with local principal investigators (L-PI) to ensure that the trial is conducted locally according to protocol and in an expeditious manner. The organisational structure and responsibilities are outlines below.

10.1 PRINCIPAL INVESTIGATORS

The chief investigator and clinical coordinators have overall responsibility for:

- Design and conduct of the study
- Preparation of the Protocol and subsequent revisions
- Managing the Trial Coordinating Centre
- Development of SOPs

10.2 TRIAL STEERING COMMITTEE

A Trial Steering Committee (TSC) will be established in line with HTA guidance, consisting of the chief Investigator, clinical coordinators, trial manager, trial statistician, patient representative, an independent chair and at least 1 other independent member will be formed and will meet on a 6-monthly basis to discuss trial progress. The TSC is responsible for:

- Agreement of the final Protocol
- Agreeing the Data Analysis Plan
- Reviewing progress of the study and, if necessary, agreeing changes to the Protocol
- Reviewing new studies that may be of relevance
- Review and approval of study reports

10.3 DATA MONITORING COMMITTEE

The independent Data Monitoring Committee (DMC) will be established in line with HTA guidance will focus on the rights, safety and wellbeing of study participants. DMC responsibilities are:

- Reviewing unblinded interim data according to the schedule agreed by all DMC members.
- Advising the Steering Committee if, in their view, the randomised data provide evidence that may warrant early termination for either safety or efficacy.

10.4 TRIAL COORDINATING CENTRE

The Trial Coordinating Centre (TCC) is responsible for the overall coordination of the Study, including:

- Study planning and organisation of Steering Committee meetings
- Agreement of each local recruitment plan
- Contractual issues with local study sites
- Ethics Committee applications
- Design, implementation and maintenance of IT systems for the study
- Auditing and monitoring of overall progress of the study
- Clinical safety monitoring (including the reporting of all “related” SAEs to the Chair of the DMC and Ethics Committee)
- Liaison with the Data Monitoring Committee and (where appropriate) with regulatory authorities and other outside agencies
- Responding to technical and administrative queries from local study sites

10.5 LOCAL STUDY SITES

The local principal investigators (L-PI) and clinical staff at the local study sites are responsible for:

- Obtaining local R&D and management approval (aided by the Trial Coordinating Centre)
- Provision of adequate clinic space and the identification of potentially eligible participants
- Conducting study procedures and follow-up according to study protocol
- Dealing with routine enquiries from participants and their families
- Obtaining appropriate information to confirm potential primary and secondary study endpoints
- Attend annual EVRA Study Collaborator Meetings to discuss study progress

11. DOCUMENT RETENTION

Data will be stored for a minimum of 10 years following completion of this trialin accordance with the Imperial College JCRO Archiving Study Documents SOP. Data generated by this work will be processed in accordance with the Data Protection Act 1998.

12. PUBLICATION POLICY

The findings will be disseminated to General Practitioners, nursing staff, surgeons and other health care professionals at regular research and educational meetings organised at local, regional, national and international levels. All analyses will be performed in compliance with a predefined analysis plan. The chief investigator, clinical coordinators and trial coordinator will be responsible for drafting the main reports from the study. Draft copies of any manuscripts will be provided to local principal investigators at each local study site, TSC members and all other collaborators for review prior to publication. The results will be put forward for critical peer review with a view to publication in relevant medical and nursing journals.

13. REFERENCES

1. Obermayer A, Garzon K. Identifying the source of superficial reflux in venous leg ulcers using duplex ultrasound. *Journal of vascular surgery* 2010;52(5):1255-61.
2. Graham ID, Harrison MB, Nelson EA, Lorimer K, Fisher A. Prevalence of lower-limb ulceration: a systematic review of prevalence studies. *Adv Skin Wound Care* 2003;16(6):305-16.
3. Callam MJ, Ruckley CV, Harper DR, Dale JJ. Chronic ulceration of the leg: extent of the problem and provision of care. *Br Med J (Clin Res Ed)* 1985;290(6485):1855-6.
4. Callam MJ, Harper DR, Dale JJ, Ruckley CV. Chronic ulcer of the leg: clinical history. *Br Med J (Clin Res Ed)* 1987;294(6584):1389-91.
5. Centre THaSCI. Statistics on obesity, physical activity and diet: England, 2012. 2012.
6. Laing W. Chronic Venous Diseases of the Leg. In: Economics OoH, editor. London, 1992.
7. Barwell JR, Davies CE, Deacon J, Harvey K, Minor J, Sassano A, et al. Comparison of surgery and compression with compression alone in chronic venous ulceration (ESCHAR study): randomised controlled trial. *Lancet* 2004;363(9424):1854-9.
8. van Gent WB, Hop WC, van Praag MC, Mackaay AJ, de Boer EM, Wittens CH. Conservative versus surgical treatment of venous leg ulcers: a prospective, randomized, multicenter trial. *Journal of vascular surgery : official publication, the Society for Vascular Surgery [and] International Society for Cardiovascular Surgery, North American Chapter* 2006;44(3):563-71.
9. Grabs AJ, Wakely MC, Nyamekye I, Ghauri AS, Poskitt KR. Colour duplex ultrasonography in the rational management of chronic venous leg ulcers. *The British journal of surgery* 1996;83(10):1380-2.
10. Adam DJ, Naik J, Hartshorne T, Bello M, London NJ. The diagnosis and management of 689 chronic leg ulcers in a single-visit assessment clinic. *Eur J Vasc Endovasc Surg* 2003;25(5):462-8.
11. Tassiopoulos AK, Golts E, Oh DS, Labropoulos N. Current concepts in chronic venous ulceration. *Eur J Vasc Endovasc Surg* 2000;20(3):227-32.
12. Gohel MS, Barwell JR, Taylor M, Chant T, Foy C, Earnshaw JJ, et al. Long term results of compression therapy alone versus compression plus surgery in chronic venous ulceration (ESCHAR): randomised controlled trial. *Bmj* 2007;335(7610):83.
13. Browse NL, Burnand KG. The cause of venous ulceration. *Lancet* 1982;2(8292):243-5.
14. Ibegbuna V, Delis KT, Nicolaides AN, Aina O. Effect of elastic compression stockings on venous hemodynamics during walking. *Journal of vascular surgery : official publication, the Society for Vascular Surgery [and] International Society for Cardiovascular Surgery, North American Chapter* 2003;37(2):420-5.
15. van den Bremer J, Moll FL. Historical overview of varicose vein surgery. *Annals of vascular surgery* 2010;24(3):426-32.
16. Winterborn RJ, Foy C, Earnshaw JJ. Causes of varicose vein recurrence: late results of a randomized controlled trial of stripping the long saphenous vein. *Journal of vascular surgery : official publication, the Society for Vascular*

Surgery [and] International Society for Cardiovascular Surgery, North American Chapter 2004;40(4):634-9.
17. O'Hare JL, Earnshaw JJ. Randomised clinical trial of foam sclerotherapy for patients with a venous leg ulcer. *Eur J Vasc Endovasc Surg* 2010;39(4):495-9.
18. Darwood RJ, Gough MJ. Endovenous laser treatment for uncomplicated varicose veins. *Phlebology* 2009;24 Suppl 1:50-61.
19. Gohel MS, Davies AH. Radiofrequency ablation for uncomplicated varicose veins. *Phlebology* 2009;24 Suppl 1:42-9.
20. Carradice D, Mekako AI, Mazari FA, Samuel N, Hatfield J, Chetter IC. Randomized clinical trial of endovenous laser ablation compared with conventional surgery for great saphenous varicose veins. *The British journal of surgery* 2011;98(4):501-10.
21. Subramonia S, Lees T. Randomized clinical trial of radiofrequency ablation or conventional high ligation and stripping for great saphenous varicose veins. *The British journal of surgery* 2010;97(3):328-36.
22. van den Bos R, Arends L, Kockaert M, Neumann M, Nijsten T. Endovenous therapies of lower extremity varicosities: a meta-analysis. *Journal of vascular surgery : official publication, the Society for Vascular Surgery [and] International Society for Cardiovascular Surgery, North American Chapter* 2009;49(1):230-9.
23. (SIGN) SIGN. *Management of chronic venous leg ulcers*: NHS, 2010.
24. Kulkarni SR, Slim FJ, Emerson LG, Davies C, Bulbulia RA, Whyman MR, et al. Effect of foam sclerotherapy on healing and long-term recurrence in chronic venous leg ulcers. *Phlebology* 2012.
25. Pang KH, Bate GR, Darvall KA, Adam DJ, Bradbury AW. Healing and recurrence rates following ultrasound-guided foam sclerotherapy of superficial venous reflux in patients with chronic venous ulceration. *Eur J Vasc Endovasc Surg* 2010;40(6):790-5.
26. Michaels JA, Campbell WB, Brazier JE, Macintyre JB, Palfreyman SJ, Ratcliffe J, et al. Randomised clinical trial, observational study and assessment of cost-effectiveness of the treatment of varicose veins (REACTIV trial). *Health Technol Assess* 2006;10(13):1-196, iii-iv.

Appendix 1: Summary of assessments and follow-up visits

Time point	Estimated duration (mins)	Clinical evaluation*	Telephone follow-up**	Wound review/photo /tracing***	Collection of further endovenous intervention / duplex report	Venous duplex	Randomisation	Consent	Health Questionnaires (EQ-5D, SF-36, AVVQ)
Screening Visit	45	X				X		X[a]	
Baseline Visit	60-90	X		X***			X	X[b]	X
1 month	30		X						
6 weeks	60-90	X		X[?]***		X[c]			X
2 months	30		X	X[?]					
3 months	30		X	X[?]					
4 months	30		X	X[?]					
5 months	30		X	X[?]					
6 months	30		X	X[?]					X
7 months	30		X	X[?]					
8 months	30		X	X[?]					
9 months	30		X	X[?]					
10 months	30		X	X[?]					
11 months	30		X	X[?]					
12 months	30		X	X[?]					X
Extension follow-up Oct18 to Mar 19	240	X[d]	X		X			X[e]	X

*. Demographic details (age, sex, ethnicity), Pregnancy test for woman of child bearing potential. General clinical details (body mass index, ankle brachial pressure index – performed within previous 4 weeks, comorbidities, medication history). Ulcer details (duration, progression, previous ulcer history, size of current ulcer – using photography and planimetry). Details of venous disease (previous deep vein thrombosis, previous venous interventions, pattern of venous reflux on duplex)

**. Ulcer healing assessment, compression type, AE assessment, Concomitant medications, health resource use. ***tracing only performed at baseline & 6 weeks

a. Approached b Taken c. Only for those who have early endovenous treatment d. review of clinical notes only e. patients will not be reconsented as already consented for longer term follow-up at outset but will be asked if they wish to continue

?dependant on whether the ulcer has healed photo will be taken at verification visit and taken weekly for 4 weeks, unless otherwise confirmed by the trial manager. Once the ulcer has healed the patient will still be followed up with monthly phone calls.

Summary of Protocol Amendments:

Protocol Version	Protocol Date	Ethics Approval Date	Changes from previous version
2	06.01.2014	05.02.2014	- Addition of abbreviation table<br>- Addition of the sponsor, IRAS and UKCRN numbers<br>- 'randomized' replaced with English spelling 'randomised' throughout the document<br>- Study setting (section 4.2, page 12) amended to clarify that additional centres may join the trial at a later date<br>- Definition of ulcer healing clarified (Page 13), 'For the purposes of the trial healing will be defined as the complete re-epithelialisation of the ulcerated leg in the absence of a scab with no dressing required. Healing cannot be assumed if a scab present.'<br>- 4.3.2 Secondary outcome measures section amended to include 'ulcer reccurrence'<br>- 'Assessment of range of ankle movement' removed from the baseline assessment section (Page 17)<br>- Section 5.4 amended to clarify that patients can be offered intervention in the standard care (compression arm) if their ulcer has not healed at 6 months<br>- Statistics and Data Analysis (section 7, Page 20) amended to reference Imperial College JRCO Archiving Policy<br>- Statistics and Data Analysis', sample size paragraph (section 7.1, Page 20) amended for clarity<br>- Statistics and Data Analysis', planned analysis paragraph (section 7.2, Page 20) amended for clarity of per-protocol analyses<br>- Statistics and Data Analysis' section (Page 21). Addition of sections 7.3 Missing, Unused and Spurious Data & 7.4 Deviations from the statistical analysis plan added<br>- Statistics and Data Analysis' section. Interim analyses: role of the Data Monitoring Committee (Page 22) removed / simplified into Section 10.3 to refer to the DMC charter. Interim analyses are now fully described in section 7.2 and the role of the DMC in section 10.3<br>- Amend all multilayer bandage compression to multilayer bandage compression / stockings<br>- Serious adverse event (section 8.2) amended to clarify that SAEs should be reported to the Trial Manager by entering the data into InForm within 24 hours of becoming aware of the event. Section also amended to confirm that all SAEs will be reported to the sponsor and DMC but only related, unexpected SAEs will be reported to the ethics committee<br>- Serious adverse event (section 8.2) amended to list all the expected adverse reactions
3	10.03.2014	25.03.2014	- Section 3.1 (Page 10) amended to add 'Patients will be referred to secondary care as part of the standard care pathway as per the July 2013 NICE Guidelines. To aid recruitment, selected Primary Care

			trusts not currently involved in the trial will be set-up as Patient Identification Centres (PIC sites) displaying posters, leaflets and disseminating patient information sheets to patients. Selected Primary Care trusts involved in follow-up of the trial (research sites) will also aid recruitment by displaying posters, leaflets and disseminating patient information sheets to patients. Patients will still need to be referred to the secondary care recruiting sites to be consented and randomised into the trial.'<br>- Section 4.2 Study Setting (Page 14) amended to add 'As per section 3.1 Primary Care Trusts will be set-up as either PIC sites or research sites aiding recruitment by displaying posters, leaflets and disseminating patient information sheets. Patients will still need to be referred to the secondary care recruiting sites to be consented and randomised into the trial. '<br>- Section 4.2 Study Setting (Page 14) amended to add the additional secondary care sites who will recruit into the study.
4	16.03.2016	12.04.2016	- 7.1 SAMPLE SIZE CALCULATION amended to state 'to identify a difference in 24-week healing rates of 15% between the two groups (60% vs 75%) with 90% power and allowing for 10% dropout the study will therefore require 416 subjects (208 in each arm, 254 healed leg ulcers in total).' 'To incorporate further allowances for protocol violations and unexpected dropouts, the target sample size will be 450 patients.'<br>- 4.3.1 Primary outcome measure amended to add 'Verification will be by clinical assessment and digital photography, to be repeated weekly for 4 weeks, **unless otherwise agreed by the trial manager'**.<br>- 6.5 URGENT VERIFICATION VISIT amended to state 'A member of the local research team will perform the four verification visits to confirm healing. Photographs will be taken and send to the Trials Unit for independent verification. **In order to minimise inconvenience to the participants, once core labs confirms healing it is not necessary for the research team to perform further verification visits. Please note all four photos should be taken unless the trial manager confirms otherwise..'**<br>- Appendix 1: Summary of assessments and follow-up visits amended to state 'dependant on whether the ulcer has healed, tracing and photo will be taken at verification visit and taken weekly for 1 month, **unless otherwise confirmed by the trial manager'**<br>- Sponsor address updated
5	06.04.2017	24.05.2017	- To incorporate the HTA funding extension to the trial to allow for the collection of longer term follow-up during October 2018 and March 2019.<br>- Amendments to the health economics section to clarify some items which were unclear in the previous version, and updates the protocol to reflect new NIHR guidelines.

Statistical Analysis Plan (SAP)

For the extension of

"A randomized clinical trial to compare early versus delayed endovenous treatment of superficial venous reflux in patients with chronic venous ulceration – EVRA"

Chief Investigator: Professor Alun H Davies

**ISRCTN**:	ISRCTN02335796
**NRES Ref:**	13/SW/0199
**SAP Version:**	0.5
**Date:**	20th May 2019

Prepared by Matyas Szigeti (Trial Statistician)

based on the main phase SAP by by Xinxue Liu & Jane Warwick

This statistical analysis plan is based on protocol version 5.0 [06/04/2017]

Table of Contents

1 Approval Signatures....4
2 Introduction....5
2.1 Study Objectives....6
2.1.1 Primary Objective....6
2.2 Study Population....6
2.3 Study Design....6
2.4 Data collection during the extension period....6
2.5 Long term outcomes....7
2.5.1 Primary outcome....7
2.5.2 Secondary outcomes....7
2.6 Study Sample Size....7
2.7 Randomisation....8
2.8 Schedule of Time....8
3 General Considerations....9
3.1 Analysis Strategy....9
3.2 Definition of Population for Analysis....10
3.3 Data Management....11
3.4 Missing Data....11
3.5 Level of Significance....11
3.6 Losses to Follow-up and Withdrawals....11
3.7 Deviations from the SAP....12
4 Analysis Plan....13

4.1 Recruitment Details ... 13
4.2 Baseline Characteristics ... 13
4.3 Primary Endpoint ... 13
4.4 Time to ulcer healing ... 13
4.5 Ulcer reoccurrence rate ... 14
4.6 Ulcer free time ... 14
4.7 Healing of recurrent ulcer ... 14
4.8 Compliance of compression bandaging ... 15
4.9 Quality of life ... 15
4.10 Safety data analysis ... 15

1 Approval Signatures

Version 0.5 approved by:

Name	Signature	Role	Date
Prof Alun H Davies	[signature]	Chief Investigator	02/07/2019
Prof Julie Brittenden	[signature]	TSC Chair	06/06/2019
Dr Jane Warwick	J. Warwick	Senior Statistician	02/07/2019
Matyas Szigeti		Trial Statistician	

1 Approval Signatures

Version 0.5 approved by:

Name	Signature	Role	Date
Prof Alun H Davies		Chief Investigator	
Prof Julie Brittenden		TSC Chair	
Dr Jane Warwick		Senior Statistician	
Matyas Szigeti	Szigeti Matyas	Trial Statistician	20/05/2019

2 Introduction

Chronic leg ulcers are open “sores” on the lower limbs situated between the ankles and knees, which fail to heal within 6 weeks. The underlying cause of leg ulceration in over 70% of cases is lower limb venous dysfunction, sometimes evident as varicose veins but often undetectable by visual examination alone. The estimated overall prevalence of active venous ulceration is as high as 1.5 to 1.8 per 1000 population, increasing to 3.8 per 1000 population in those over 40 years of age. As patients with venous ulceration usually suffer episodes of recurrence between periods when the ulcer remains healed, the number of patients with a high risk of ulceration may actually be 4-5 fold higher.

Venous ulcers are characterised by protracted healing times. Despite some recent advances in the management of patients with venous ulcers, 24 week healing rates in published randomized trials are around 60-65%, and the true population healing rates are likely to be significantly lower.

For over a century, the treatment of superficial venous reflux has involved operative ligation and surgical stripping of the vein and avulsion of bulging varicose veins. Until recent years, open surgery has been considered the definitive treatment option for superficial venous reflux. However, the operation usually requires general anaesthesia and patients often suffer discomfort, bruising and significant time off work in the post-operative period. In addition, long-term studies have also identified significant complications of open surgery. In response to this high complication rate and a growing patient desire for less invasive treatments, a range of novel, minimally invasive endovenous treatment options have been developed and have gained in popularity over the last decade. Non-randomized studies suggest that outcomes may be improved by treating underlying superficial reflux using the latest technologies, but there is no robust evidence to support early intervention. Therefore, we believe that there is a cogent argument for conducting this trial at this time.

2.1 Study Objectives

2.1.1 Primary Objective

To study the long term effectiveness of early endovenous treatment of superficial venous reflux in addition to standard care compared to standard care alone in patients with chronic venous ulceration.

2.2 Study Population

Patients with leg ulceration referred to secondary care as part of the standard care pathway.

2.3 Study Design

The EVRA ulcer trial is a pragmatic, multicentre randomized clinical trial with participants randomized 1:1 to either:

'Standard' therapy consisting of multilayer elastic compression bandaging / stockings with deferred treatment of superficial reflux (usually once the ulcer has healed)

Early endovenous treatment of superficial venous reflux (within 2 weeks) in addition to standard therapy

With the extension, the length of follow up is up to 5 years (median approximately 3.7 years).

2.4 Data collection during the extension period

For each randomised patient a single telephone assessment will be performed between 1st October 2018 and 31st March 2019 to collect:

- Details of any further ulcer recurrence and healing events
- Assessment of ulcer related healthcare attendances and costs

• Details of all further venous interventions performed and any associated adverse events

• Assessments of disease specific and generic quality of life (AVVQ, EQ5D & SF36) by means of self-completed questionnaire completed over the telephone (or via post)

2.5 Long term outcomes

All of the outcomes applied on the whole study data including data collected during the main part and the extension unless it's stated otherwise.

2.5.1 Primary outcome

The primary outcome measure will be time to the first ulcer recurrence on the randomised leg from date of first ulcer healed to date of recurrence.

2.5.2 Secondary outcomes

1. Time to healing of first ulcer
2. Ulcer recurrence rate
3. Ulcer free time
4. Time to healing of recurrent ulcer
5. Compliance with compression bandaging
6. Quality of life (AVVQ, EQ5D & SF36)
7. Cost-effectiveness/health economic analysis

2.6 Study Sample Size

Assuming that 90% of EVRA participants experience ulcer healing and 15% withdraw or are lost to follow up, 344 participants will be available for the analysis of long term outcomes (450 x 0.9 x 0.85). All patients that have not formally withdrawn or died will be followed-up. The recurrence rate is expected to be 38%, based on evidence from the ESCHAR trial and it is known that approximately 95% of participants in the early arm and 85% in the delayed arm experienced ulcer healing by 12 months. With 344 participants (182

in the early arm and 162 in the delayed arm), the study will have 82% power to detect an absolute difference in recurrence rate of 15% (30% early arm vs 45% delayed arm) and 97% power to detect an absolute difference in recurrence rate of 20% (30% early arm vs 50% delayed arm).

2.7 Randomisation

Not applicable

2.8 Schedule of Time

Data collection for the longer term follow-up will be between 1st October 2018 and the 31st March 2019.

3 General Considerations

3.1 Analysis Strategy

All the analyses will be on an intention-to-treat basis. Histograms and boxplots will be used to check the distribution and possible outliers for continuous variables. Mathematical transformations might be applied, where appropriate, in order to render the continuous variables' distribution normally distributed. Continuous variables that follow an approximately normal distribution will be summarised using means and standard deviations. Skewed continuous variables will be summarised using medians and inter-quartile ranges. Categorical variables will be summarised using frequencies and percentages.

Because the drop-out might depend on the treatment effect and might cause systematic difference between the two arms we will adjust for the most influential predictors and take adjusted results as the primary results.

In the late intervention arm it is possible that some patients don't get the intervention at all which is very unlikely in the early intervention group but could be very influential on the treatment effect so we will adjust for that the patient had the endovascular surgery or not. The other adjustment factors are age, ulcer size and ulcer chronicity (ulcer age).

For the primary outcome (time to the first recurrence) we will test the hypothesis that there is no difference in this between the control and intervention groups using a Cox model with study centre as a random effect. The primary result will be adjusted for age, ulcer size and ulcer chronicity and receipt of the intervention as per protocol. Kaplan-Meier survival curves will also be presented. The time to the first recurrence is defined from the date when the first ulcer healed to the date of first recurrence.

Time to ulcer healing will also be assessed using a Cox model with centre as random effect and adjusted for age, ulcer size and ulcer chronicity. Kaplan-Meier curves will also be presented, where numbers allow, by randomisation group (2 curves) and by randomisation group and receipt of intervention per protocol (4 curves).

Ulcer recurrence rate will be obtained from the Kaplan-Meier analysis of the primary outcome and the rates in each arm will be tabulated for appropriate time points (say 2 years, 4 years) with associated 95% confidence intervals.

Ulcer free time will be summarised using appropriate descriptive methods, including mean or median ulcer free time will be presented at appropriate time points for each group.

To account for differences in follow up time, the difference in ulcer free time between the treatment groups will be assessed using Cox regression adjusted for the same factors as the primary outcome.

Time to healing of the recurrent ulcer (from date of recurrence to date of healing) will also be assessed similarly as the primary outcome using Cox model with centre and patient as random effect and will be visualised by Kaplan-Meier curves.

Compliance will be compared between the two arms and possible relationships and patterns between subgroups and other recorded factors that might influence compliance will be explored.

Quality of life measurements will be compared between arms using ANOVA and will be summarised for each timepoint.

Health economic assessment will be carried by the trial health economist and thus will not be included in this statistical analysis plan (See Extension Health Economic Analysis Plan).

3.2 Definition of Population for Analysis

The main analysis will be “intention to treat” (ITT) based and will include all participants who were randomised as they were randomised. For the analysis of ulcer free time, the population for analysis will be patients with complete follow-up data only. This is because ulcer free time to the end of follow-up depends on the time of primary ulcer healing and duration of recurrent ulcer (for example, patients with ulcer free time of 0 day may have an unhealed primary ulcer at the end of follow-up, or may have withdrawn from

the study after healing at month 1, or may have withdrawn from the study after healing at month 11). By adding this constraint some bias may have been introduced (as the analysis will have been based on complete cases only) but ulcer free time will have only one interpretation. As a sensitivity analysis, the analysis of ulcer free time will therefore be repeated using all the patients, irrespective of length of follow up. This should give a very conservative estimate of the treatment effect.

3.3 Data Management

Data is collected and managed using InForm: an electronic data capture system built around an Oracle database. The InForm system includes validation rules for data entry to help ensure data accuracy, and has a full audit trial of data entry and changes. Data queries will be raised for inconsistent, impossible or missing data.

3.4 Missing Data

There will be no data imputation for missing data in any outcome. However, the level and pattern of the missing data in the baseline variables and outcomes will be reported. The potential causes of any missingness will be investigated and documented as far as possible. Any missing data will be dealt with using methods appropriate to the conjectured missingness mechanism and level of missingness.

3.5 Level of Significance

The primary outcome and secondary outcomes will be tested using a two-tailed hypothesis test with a 5% significance level. For secondary outcomes, there will be no adjustment for multiple testing.

3.6 Losses to Follow-up and Withdrawals

All the primary analyses will be performed on an intention-to-treat basis. Only patients willing to undergo either immediate or delayed superficial venous ablation with compression bandaging are randomised. Subjects who die, withdraw from the study, or are

lost to follow-up before ulcer healing will be censored in the Kaplan Meier and Cox regression analyses at last follow-up visit.

3.7 Deviations from the SAP

All deviations from the SAP will be disclosed in the final analysis report. If problems or fundamental issues become apparent in the on-going checking that forms part of the statistical analysis, the trial statistician will raise these with a senior statistician who will consult with the appropriate individuals. Any such action and subsequent decisions will be documented in the final statistical analysis report.

4 Analysis Plan

4.1 Recruitment Details

Details about patient enrolment, follow-up and inclusion in analysis will be provided using a consort diagram. This means that the main phase's consort diagram will be extended with the extensions follow up data.

4.2 Baseline Characteristics

Baseline characteristics would be the same as in the main phase thus won't be presented. Instead of that a CONSORT diagram will summarise the follow up details and the reasons for drop out.

4.3 Primary Endpoint

The primary outcome is time to the first recurrence from the time first ulcer was healed. We will test the hypothesis that there is no difference in this between the control and intervention groups using a Cox model with study centre as a random effect. Kaplan-Meier survival curves and the log-rank test result will also be presented. Both unadjusted and adjusted (age, ulcer chronicity, ulcer size, receival of the intervention as per protocol) Hazard Ratios (HR) and their 95% Confidence Interval (CI) will be presented but the adjusted results will taken as primary. For Cox regression models the proportionality assumption will be assessed graphically (using diagnostic plots).

4.4 Time to ulcer healing

Time to ulcer healing is defined as the time from the date of randomisation to the ulcer healing on the randomised leg. Similarly to the primary outcome we will test the hypothesis that there is no difference in this between the control and intervention groups using a unadjusted and adjusted Cox model with study centre as a random effect. Adjusted results will be taken as primary and the adjustment factors will be age, ulcer chronicity,

ulcer size and receival of the intervention as per protocol. Kaplan-Meier survival curves and the log-rank test result and HR with 95% CI will be also presented.

4.5 Ulcer recurrence rate

Ulcer recurrence rate defined as the proportion of patients who had an ulcer recurrence until a certain timepoint. This will be obtained from the Kaplan-Meier analysis of the primary outcome and the rates in each arm will be tabulated for appropriate time points (say 2 years, 4 years) with associated 95% confidence intervals.

Ulcer free time

Ulcer free time is defined as the time between randomisation and the end of follow-up when the randomised leg was free of ulcer. The preferred method of analysis is to use Cox regression to assess the difference between the treatment arms, with centre as a random effect, unadjusted and with adjustment for age, ulcer size and ulcer chronicity and the length of follow up. In the case that a patient is dead, withdrawn or lost to follow-up, ulcer free time will be calculated as the time from randomisation until last follow-up. Graphical methods will be used to assess whether the assumptions aremet. If the assumptions arenot met, and there is no suitable transformation, ulcer free time will be compared by ordinal regression or negative binomial regression or multiple events survival modelling. Model fit will be assessed using residual plots and/or goodness-of-fit tests, as appropriate.

4.6 Healing of recurrent ulcer

Healing of a recurrent ulcer (any) is defined as the time between the date of the recurrence and the date of the healing of the reoccurred ulcer. This could happen multiple times per patient and all of the recurrent ulcers will taken into the analysis. The healing time of recurrent ulcers will be assessed by three level Cox regression with study centre and patient as a random effect. This will be also adjusted for age, ulcer size and ulcer chronicity

and endovascular therapy. Kaplan-Meier survival curves and the log-rank test result and HR with 95% CI will be also presented.

4.7 Compliance of compression bandaging

Compliance will be compared between the two arms and possible relationships and patterns between subgroups and other recorded factors that might influence compliance will be explored.

4.8 Quality of life

The quality of life questionnaires include disease specific (AVVQ) and generic (EQ5D & SF-36) components. AVVQ will be recoded according to its manual. The SF-36 will be scored using Health Outcome Scoring Software 5.1 for the physical health and mental health dimensions, and all eight scales, including physical functioning, role limitations due to physical health, role limitations due to emotional problems, energy/fatigue, emotional well-being, social functioning, pain, and general health.

The QoL scores will be presented using line plots for each study arm to illustrate trends in AVVQ score, SF-36 and EQ-5D-5L over time. Depending on the distribution of the data, the means and 95% CI of means or medians and inter-quartile ranges at baseline, 6-weeks, 6-months and 12-months after randomisation and at the end of follow up, will be reported. Analysis of variance will be used to explore changes in QoL over time and assess the difference between the two intervention groups.

4.9 Safety data analysis

Adverse events will be summarised by the following tables:

- number of adverse events and the number of subjects who have adverse events by category and treatment group;
- number of adverse events by category and relationship to study treatment;

- number of adverse events reported for individual subjects by treatment group.

In addition, the following listings will be produced:

- All serious adverse events

Statistical Analysis Plan (SAP)

For the extension of

"A randomized clinical trial to compare early versus delayed endovenous treatment of superficial venous reflux in patients with chronic venous ulceration – EVRA"

Chief Investigator: Professor Alun H Davies

**ISRCTN**:	ISRCTN02335796
**NRES Ref:**	13/SW/0199
**SAP Version:**	3.0
**Date:**	16th August 2019

Prepared by Matyas Szigeti (Trial Statistician)

based on the main phase SAP by by Xinxue Liu & Jane Warwick

This statistical analysis plan is based on protocol version 5.0 [06/04/2017]

Table of Contents

1 Approval Signatures....4

2 Introduction....5

2.1 Study Objectives....6

2.1.1 Primary Objective....6

2.2 Study Population....6

2.3 Study Design....6

2.4 Data collection during the extension period....6

2.5 Long term outcomes....7

2.5.1 Primary outcome....7

2.6 Study Sample Size....8

2.7 Randomisation....8

2.8 Schedule of Time....8

3 General Considerations....9

3.1 Analysis Strategy....9

3.2 Definition of Population for Analysis....10

3.3 Data Management....11

3.4 Missing Data....11

3.5 Level of Significance....11

3.6 Losses to Follow-up and Withdrawals....12

3.7 Deviations from the SAP....12

4 Analysis Plan....13

4.1 Recruitment Details....13

4.2 Baseline Characteristics 13
4.3 Time to ulcer recurrence 13
4.4 Time to ulcer healing 14
4.5 Ulcer recurrence rate 14
4.6 Ulcer free time 14
4.7 Healing of recurrent ulcer 15
4.8 Compliance of compression bandaging 15
4.9 Quality of life 15
4.10 Safety data analysis 16

1 Approval Signatures

Version 3.0 approved by:

Name	Signature	Role	Date
Prof Alun H Davies		Chief Investigator	22/08/19
Prof Julie Brittenden		TSC Chair	22/08/19
Prof Jane Warwick		Senior Statistician	22/08/19
Matyas Szigeti		Trial Statistician	22/08/19

2 Introduction

Chronic leg ulcers are open “sores” on the lower limbs situated between the ankles and knees, which fail to heal within 6 weeks. The underlying cause of leg ulceration in over 70% of cases is lower limb venous dysfunction, sometimes evident as varicose veins but often undetectable by visual examination alone. The estimated overall prevalence of active venous ulceration is as high as 1.5 to 1.8 per 1000 population, increasing to 3.8 per 1000 population in those over 40 years of age. As patients with venous ulceration usually suffer episodes of recurrence between periods when the ulcer remains healed, the number of patients with a high risk of ulceration may actually be 4-5 fold higher.

Venous ulcers are characterised by protracted healing times and frequent recurrences. Despite some recent advances in the management of patients with venous ulcers, 24 week healing rates in published randomized trials are around 60-65%, and the true population healing rates are likely to be significantly lower.

For over a century, the treatment of superficial venous reflux has involved operative ligation and surgical stripping of the vein and avulsion of bulging varicose veins. Until recent years, open surgery has been considered the definitive treatment option for superficial venous reflux. However, the operation usually requires general anaesthesia and patients often suffer discomfort, bruising and significant time off work in the post-operative period. In addition, long-term studies have also identified significant complications of open surgery. In response to this high complication rate and a growing patient desire for less invasive treatments, a range of novel, minimally invasive endovenous treatment options have been developed and have gained in popularity over the last decade. Non-randomized studies suggest that outcomes may be improved by treating underlying superficial reflux using the latest technologies. The EVRA study was the first randomised trial to demonstrate improved healing outcomes after early intervention. An understanding of medium and long-term outcomes will allow greater appreciation of the impact of early endovenous intervention beyond the first year.

2.1 Study Objectives

2.1.1 Primary Objective

To study the long term effectiveness of early endovenous treatment of superficial venous reflux in addition to standard care compared to standard care alone in patients with chronic venous ulceration.

2.2 Study Population

Patients with leg ulceration referred to secondary care as part of the standard care pathway.

2.3 Study Design

The EVRA ulcer trial is a pragmatic, multicentre randomized clinical trial with participants randomized 1:1 to either:

'Standard' therapy consisting of multilayer elastic compression bandaging / stockings with deferred treatment of superficial reflux (usually once the ulcer has healed)

Early endovenous treatment of superficial venous reflux (within 2 weeks) in addition to standard therapy

With the study extension, the duration of follow-up is up to 5 years (median follow-up approximately 3.7 years).

2.4 Data collection during the extension period

For each randomised patient a single telephone assessment will be performed between 1st October 2018 and 31st March 2019 to collect:

• Details of any further ulcer recurrence and healing events

• Assessment of ulcer related healthcare attendances and costs

• Details of all further venous interventions performed and any associated adverse events

• Assessments of disease specific and generic quality of life (AVVQ, EQ5D & SF36) by means of self-completed questionnaire completed over the telephone (or via post)

2.5 Long term outcomes

All outcomes will be evaluated using data from the entire study (main study and extended follow-up period) unless stated otherwise.

2.5.1 Primary outcome

The primary outcome measure will be time to first ulcer recurrence on the randomised leg from date of healing. For the purposes of this study, ulcer healing is defined as complete re-epithelialisation of all ulceration on the randomised (reference) leg in the absence of a scab (eschar) with no dressing required.

2.5.2 Secondary outcomes

1. Time to the first ulcer recurrence on the randomised leg from the date of randomisation.
2. Ulcer recurrence rate
3. Time to healing of initial (index) ulcer
4. Ulcer free time
5. Time to healing of recurrent ulcers
6. Compliance with compression bandaging
7. Quality of life (AVVQ, EQ5D & SF36)
8. Cost-effectiveness/health economic analysis

2.6 Study Sample Size

Assuming that 90% of EVRA participants experience ulcer healing and 15% withdraw or are lost to follow up, 344 participants will be available for the analysis of long-term outcomes (450 x 0.9 x 0.85). All patients that have not withdrawn from the study or died will be followed-up. The ulcer recurrence rate is expected to be 38%, based on evidence from the ESCHAR trial and it is known that approximately 95% of participants in the early arm and 85% in the delayed arm experienced ulcer healing by 12 months. With 344 participants (182 in the early arm and 162 in the delayed arm), the study will have 82% power to detect an absolute difference in recurrence rate of 15% (30% early arm vs 45% delayed arm) and 97% power to detect an absolute difference in recurrence rate of 20% (30% early arm vs 50% delayed arm).

2.7 Randomisation

Not applicable

2.8 Schedule of Time

Data collection for the long-term follow-up will be between 1st October 2018 and 31st March 2019.

3 General Considerations

3.1 Analysis Strategy

All analyses will be on an intention-to-treat basis unless specified otherwise. Histograms and boxplots will be used to check the distribution and possible outliers for continuous variables. Mathematical transformations might be applied, where appropriate, in order to render the continuous variables' distribution normally distributed. Continuous variables that follow an approximately normal distribution will be summarised using means and standard deviations. Skewed continuous variables will be summarised using medians and inter-quartile ranges. Categorical variables will be summarised using frequencies and percentages.

For the primary outcome (time to the first recurrence), the hypothesis that there is no difference between the control and intervention groups will be tested using a Cox model (with study centre grouped by region as a random effect). The primary result will be adjusted for age, ulcer size and ulcer chronicity. Kaplan-Meier survival curves for each treatment group will also be presented. The time to the first recurrence is defined from the date of ulcer healing to the date of first recurrence.

Time to ulcer healing will also be assessed using a Cox model with centre as random effect and adjusted for age, ulcer size and ulcer chronicity. Kaplan-Meier curves for each treatment group will also be presented.

Ulcer recurrence rate will be obtained from the Kaplan-Meier analysis of the primary outcome and the rates in each arm will be calculated at annual time points with associated 95% confidence intervals.

Ulcer free time will be analysed using survival analysis (to account for censoring) and median ulcer free time, with associated 95% confidence intervals, will be presented for each group.

The difference in ulcer free time between the treatment groups will be assessed using Cox regression adjusted for the same factors as the primary outcome.

Time to healing of the recurrent ulcer (from date of recurrence to date of healing) will be assessed as for the primary outcome using Cox model with centre and patient as random effect. Kaplan-Meier curves will be used to present the data.

Compliance will be compared between the two arms and possible relationships and patterns between subgroups and other recorded factors that might influence compliance will be explored.

Quality of life measurements will be compared between arms using 3-level mixed models and will be summarised for each timepoint.

Health economic assessment will be carried by the trial health economist and thus will not be included in this statistical analysis plan (See Extension Health Economic Analysis Plan).

3.2 Definition of Population for Analysis

Analysis of the primary outcome will be on an “intention to treat” (ITT) basis and will include all participants who were randomised in their allocated groups. Participants whose first ulcer has not healed are not at risk of recurrence and so will effectively be excluded (as they will drop out from the numbers at risk). Analysis of the secondary outcomes will also be on intention to treat with all randomised participants included where possible. For time to recurrence from date of healing, participants whose first ulcer has not healed will be excluded. For time to healing of recurrent ulcer, participants without ulcer recurrence will be excluded. A secondary per-protocol analysis of time to healing and time to first recurrence (from healing) will also be carried out by excluding patients with the following protocol deviations:

1) Patients randomised to multilayer compression / stockings plus early venous reflux ablation, who receive endovenous intervention more than two weeks from randomization.

2) Patients who are non-compliant with compression bandaging, defined as use <75% of the prescribed duration.

3) Patients randomised to compression bandaging alone who undergo endovenous ablation prior to verified healing.

3.3 Data Management

Data is collected and managed using InForm: an electronic data capture system built around an Oracle database. The InForm system includes validation rules for data entry to help ensure data accuracy, and has a full audit trial of data entry and changes. Data queries will be raised for inconsistent, impossible or missing data.

3.4 Missing Data

There will be no data imputation for missing data in any outcome. However, the level and pattern of the missing data in the baseline variables and outcomes will be reported. The potential causes of any missingness will be investigated and documented as far as possible. Any missing data will be dealt with using methods appropriate to the conjectured missingness mechanism and level of missingness.

3.5 Level of Significance

The primary outcome and secondary outcomes will be tested using a two-tailed hypothesis test with a 5% significance level. For secondary outcomes, there will be no adjustment for multiple testing.

3.6 Losses to Follow-up and Withdrawals

All the primary analyses will be performed on an intention-to-treat basis. Only patients willing to undergo either immediate or delayed superficial venous ablation with compression bandaging are randomised. Subjects who die, withdraw from the study, or are lost to follow-up before ulcer healing will be censored in the Kaplan Meier and Cox regression analyses at last follow-up visit.

3.7 Deviations from the SAP

All deviations from the SAP will be disclosed in the final analysis report. If problems or fundamental issues become apparent in the on-going checking that forms part of the statistical analysis, the trial statistician will raise these with a senior statistician who will consult with the appropriate individuals. Any such action and subsequent decisions will be documented in the final statistical analysis report.

4 Analysis Plan

4.1 Recruitment Details

Details about patient enrolment, follow-up and inclusion in analysis will be provided using a consort diagram. This means that the main phase's consort diagram will be extended with the extensions follow up data.

4.2 Baseline Characteristics

Baseline characteristics would be the same as in the main phase and therefore will not be presented. Instead, a CONSORT diagram will summarise the follow up details and the reasons for drop out.

4.3 Time to ulcer recurrence

The primary outcome is time to the first recurrence from the time first ulcer was healed. We will test the hypothesis that there is no difference in this between the control and intervention groups using a Cox model with study centre as a random effect. Kaplan-Meier survival curves and the log-rank test result will also be presented. Both unadjusted and adjusted (age, ulcer chronicity, ulcer size. Hazard Ratios (HR) and their 95% Confidence Interval (CI) will be presented but the adjusted results will be taken as primary. For Cox regression models the proportionality assumption will be assessed graphically (using diagnostic plots). A secondary analysis will be undertaken as above but with time to ulcer recurrence measured from time of randomisation rather than time of healing. As a sensitivity analysis, the above will be repeated in the per-protocol population.

4.4 Time to ulcer healing

Time to ulcer healing is defined as the time from the date of randomisation to the first ulcer healing on the randomised leg. Similarly to the primary outcome we will test the hypothesis that there is no difference in this between the control and intervention groups using a unadjusted and adjusted Cox model with study centre as a random effect. Adjusted results will be taken as primary and the adjustment factors will be age, ulcer chronicity, ulcer size. Kaplan-Meier survival curves and the log-rank test result and HR with 95% CI will be also presented. As a sensitivity analysis, the above will be repeated in the per-protocol populations.

4.5 Ulcer recurrence rate

Ulcer recurrence rate defined as the proportion of patients who had an ulcer recurrence at a defined timepoint. This will be obtained from the Kaplan-Meier analysis of the primary outcome and the rates in each arm will be calculated for annual time points with associated 95% confidence intervals.

4.6 Ulcer free time

Ulcer free time is defined as the time between randomisation and the end of follow-up when the randomised leg was free of ulcer. The preferred method of analysis is to use Cox regression to assess the difference between the treatment arms, with centre as a random effect, unadjusted and with adjustment for follow-up time, age, ulcer size and ulcer chronicity. The adjusted results will be taken as primary. In the case that a patient is dead, withdrawn or lost to follow-up, ulcer free time will be calculated as the time from randomisation until last follow-up. Graphical methods will be used to assess whether the assumptions are met. If the assumptions are not met, and there is no suitable

transformation, ulcer free time will be compared by ordinal regression or negative binomial regression or multiple events survival modelling. Model fit will be assessed using residual plots and/or goodness-of-fit tests, as appropriate.

4.7 Healing of recurrent ulcer

Healing of a recurrent ulcer (any) is defined as the time between the date of the recurrence and the date of the healing of the recurrent ulcer. This could happen multiple times per patient and all of the recurrent ulcers will be included the analysis. To test for a difference between the treatment groups, healing time of recurrent ulcers will be analysed using three level Cox regression, if possible, with study centre and patient as random effects. Should the fitting of a three level model not be possible (through lack of convergence, for example), a two level model (with patient only as a random effect) will be used instead with study centre included as a fixed effect. This will be also adjusted for age, ulcer size and ulcer chronicity. Kaplan-Meier survival curves and the log-rank test result and HR with 95% CI will be also presented.

4.8 Compliance with compression bandaging

Compliance will be compared between the two arms and possible relationships and patterns between subgroups and other recorded factors that might influence compliance will be explored.

4.9 Quality of life

The quality of life questionnaires include disease specific (AVVQ) and generic (EQ5D & SF-36) components. AVVQ will be recoded according to its manual. The SF-36 will be scored

using Health Outcome Scoring Software 5.1 for the physical health and mental health dimensions, and all eight scales, including physical functioning, role limitations due to physical health, role limitations due to emotional problems, energy/fatigue, emotional well-being, social functioning, pain, and general health.

The QoL scores will be presented using line plots for each study arm to illustrate trends in AVVQ score, SF-36 and EQ-5D-5L over time. Depending on the distribution of the data, the means and SD or medians and inter-quartile ranges at baseline, 6-weeks, 6-months and 12-months after randomisation and at the end of follow up, will be reported. 3 level mixed models be used to explore changes in QoL over time and assess the difference between the two intervention groups using grouped centre and patient as random effect.

4.10 Safety data analysis

Adverse events will be summarised by the following tables:

- number of adverse events and the number of subjects who have adverse events by category and treatment group;
- number of adverse events by category and relationship to study treatment;
- number of adverse events reported for individual subjects by treatment group.

In addition, the following listings will be produced:

- All serious adverse events

Summary of SAP (extension) Amendments:

SAP Version	SAP Date	Changes from previous version
0.5	20.05.2019	1st signed version
1.0	N/A	Draft only
2.0	N/A	Draft only
3.0	16.08.2019	- Clarification of the primary and secondary outcomes<br>- Clarification of analysis methods for each outcome<br>- Addition of a per-protocol analysis for time to healing and time to first recurrence (from date of healing)<br>- Definition of the per-protocol population<br>- Grammatical changes
