# Supplementary material for: Long-term Clinical and Cost-effectiveness of Early Endovenous Ablation in Venous Ulceration: A Randomized Clinical Trial
Source: JAMA Surg. 2020 Sep 23;155(12):1113–21. doi: 10.1001/jamasurg.2020.3845 (PMC7512122; doi:10.1001/jamasurg.2020.3845)
Supplement: Supplement 2. — eMethods. Health Economic Analysis eFigure 1. Mortality Rate for Participants in the EVRA Trial eFigure 2. Time to Ulcer Recurrence From Randomization eFigure 3. Kaplan-Meier Curve for Time to Ulcer Healing for Recurrent Venous Leg Ulcers in the Early-Intervention Group and Deferred-Intervention Group eFigure 4. Time to Ulcer Healing for Primary Ulcer eFigure 5. Time to First Ulcer Recurrence eFigure 6. Cost-Effectiveness Acceptability Curve at 3 Years eFigure 7. Sensitivity Analysis Using Monte Carlo Simulation eTable 1. EVRA Study: Recruiting Centers eTable 2. Summary of Secondary Outcome Measures and Quality-of-Life Tools Used in EVRA Study eTable 3. Resource and Unit Costs Utilized for Health Economic Analysis eTable 4. Summary of Quality-of-Life Outcomes for Extended Follow-Up eTable 5. Use of Hospital and Community Health Services and Non-NHS Expenses During Extended Follow-Up Period eTable 6. Undiscounted Costs Per Patient at 3 Years in Each Treatment Group eTable 7. Difference in Discounted Total Mean Costs and Discounted QALY at Different Time Horizons Estimated Using Mixed Model eTable 8. Difference in Discounted Total Mean Costs and Discounted QALY at Different Time Horizons Estimated Using Mixed Model Per Protocol (a) eReferences [file jamasurg-e203845-s002.pdf]

Supplementary Online Content

Gohel MS, Mora J, Szigeti M, et al. Long-term clinical and cost-effectiveness of early endovenous ablation in venous ulceration: a randomized clinical trial. *JAMA Surg*. Published online September 23, 2020. doi:10.1001/jamasurg.2020.3845

eMethods. Health-Economic Analysis

eFigure 1. Mortality Rate for Participants in the EVRA Trial

eFigure 2. Time to Ulcer Recurrence From Randomization

eFigure 3. Kaplan-Meier Curve for Time to Ulcer Healing for Recurrent Venous Leg Ulcers in the Early-Intervention Group and Deferred-Intervention Group

eFigure 4. Time to Ulcer Healing for Primary Ulcer

eFigure 5. Time to First Ulcer Recurrence

eFigure 6. Cost-Effectiveness Acceptability Curve at 3 Years

eFigure 7. Sensitivity Analysis Using Monte Carlo Simulation

eTable 1. EVRA Study: Recruiting Centers

eTable 2. Summary of Secondary Outcome Measures and Quality of Life Tools Used in EVRA Study

eTable 3. Resource and Unit Costs Utilized for Health-Economic Analysis

eTable 4. Summary of Quality of Life Outcomes for Extended Follow-Up

eTable 5. Use of Hospital and Community Health Services and Non-NHS Expenses During Extended Follow-Up Period

eTable 6. Undiscounted Costs Per Patient at 3 Years in Each Treatment Group

eTable 7. Difference in Discounted Total Mean Costs and Discounted QALY at Different Time Horizons Estimated Using Mixed Model

eTable 8. Difference in Discounted Total Mean Costs and Discounted QALY at Different Time Horizons Estimated Using Mixed Model per Protocol (a)

eReferences

This supplementary material has been provided by the authors to give readers additional information about their work.

© 2020 Gohel M et al. *JAMA Surg.*

eMethods Health-Economic Analysis

The primary analysis estimated costs and quality adjusted life years (QALYs) from the perspective of the United Kingdom (UK) National Health Service (NHS) and Personal Social Services over a three-year time horizon. Patient out of pocket expenses and time lost from work and usual activities were reported descriptively (societal perspective). The price year was 2017-2018. Discounting was applied according to UK Government guidelines (3.5% per year for costs and health outcomes).[1] The study was conducted and reported according to current guidelines for economic evaluation.[2]

Resource use items in hospital and community care settings related to the treatment of venous ulceration, adverse events or complications were collected by case note review and questionnaires completed at baseline and monthly thereafter up to one year, plus one further follow up between October 2018 and March 2019. Resources reported as used by each patient were classified in one of 4 categories:

1. Primary vein ablation procedures (early or delayed)

2. Episodes of contact with healthcare professionals (hospital inpatient admissions, outpatient clinics, General Practitioner, Community nurse, Physiotherapy, Occupational therapy)

3. Wound dressings, bandaging and compression consumables

4. Medications - antiplatelets or anticoagulants

Each item of resource use was multiplied by UK unit costs obtained from published literature[3], NHS reference costs[4-8], and manufacturers' list prices to calculate overall costs within each of these categories for each patient (Table S3). It was assumed that the patient required a dressing

© 2020 Gohel M et al. *JAMA Surg.*

change twice a week while the primary ulcer or recurrent ulcer remained unhealed, and that compression bandages or stockings after healing would be replaced every 3 months. During the first year, the trial recorded use of medication each month, but during the extension, it was not known when medication started or stopped. If the patient indicated at the extension follow-up that they were using aspirin, clopidogrel or warfarin, it was assumed that the patient used the same medication continuously from the one-year follow-up to the end of the trial, at recommended daily doses. The trial recorded use of enoxaparin and new oral anticoagulants (apixaban, rivaroxaban, enoxaparin and dabigatran) at the end of every month during the first year and once more at the extension follow-up. The costs of these medicines were included up to one year, but could not be included beyond this time as it could not be assumed that use was continuous over the whole follow-up.

The costs for each individual over their follow-up (from randomization to date of censoring for that individual) were assigned or apportioned into discrete time periods, that corresponded to monthly periods during the first year (as follow-ups were monthly) and then yearly periods thereafter. This allowed discounting to be applied, and facilitated analysis using the mixed model (see below).

The cost data were reshaped into the long format and analyzed using a linear mixed model

c_it~ TREAT##i.WEEK

Where TREAT is the treatment assignment (0 for delayed and 1 for early intervention), WEEK is the period during which the cost was incurred (in monthly periods up to 1 year, then yearly periods beyond one year) and center and subject are random effects. Age, ulcer size and age of ulcer at baseline were also included as control variables. The advantage of a mixed model is that it allows all the available data to contribute to the estimate of total mean cost, even if not all follow-up time points are observed for all individuals, avoiding the need for listwise deletion of

© 2020 Gohel M et al. *JAMA Surg.*

individuals who have missing data for some time points. The coefficient on the interaction term between treatment assignment and period number represents the mean additional cost associated with early intervention during that period, compared with delayed intervention. By adding up the coefficients for these interaction terms the total mean incremental cost of the early intervention can be estimated over any chosen time horizon. Thus, for example, if δ_1 is the coefficient on the treatment group at 4 weeks (~month 1), δ_2 is the coefficient on the interaction term between treatment group and time dummy at 8 weeks (~month 2), and δ_3 is the equivalent interaction coefficient at 13 weeks (month 3), then the difference in mean total cost between the treatment groups over the first 3 months is $\delta_1 + \delta_2 + \delta_3$. Continuing this approach allowed the difference in mean total cost to be estimated over the chosen time horizon (3 years in the base-case, 4 years and 5 years in sensitivity analyses). Confidence intervals for these estimates were calculated using the lincom command in STATA.

EQ-5D-5L was collected at baseline, 6 weeks, 6 months, 12 months, plus one further follow up between October 2018 and March 2019. Utility indices for each individual at each follow up time were calculated from the EQ-5D-5L questionnaire using the so-called "crosswalk" tariff recommended by NICE[9]. As a sensitivity analysis, an alternative published tariff was employed.[10]

The EQ-5D index values were then analyzed using a linear mixed model

q_it~ TREAT##i.WEEK

As before, TREAT is the treatment assignment (0 for delayed and 1 for early intervention), WEEK is a dummy variable indicating the time at which the EQ-5D was collected (0 weeks, 6 weeks, 26 weeks, 52 weeks and one further follow-up at between 3 and 5 years) and center and subject are random effects. Age, ulcer size and age of ulcer at baseline were also included as control variables.

© 2020 Gohel M et al. *JAMA Surg.*

The coefficient on the interaction term between treatment assignment and period number represents the mean additional EQ-5D index associated with early intervention during that period, compared with delayed. Mean QALYs were then estimated from these coefficients using the "area under the curve" principle (approximated using the trapezium rule). Thus, for example, if γ_0 is the coefficient on the treatment group at baseline, γ_1 is the coefficient on the interaction term between treatment group and time dummy at 6 weeks, and γ_2 is the equivalent interaction coefficient at 6 months, then an estimate of the mean difference in QALY between the groups over the first 6 months (26 weeks) is $0.5 (\gamma_0 + \gamma_1) * 6/52 + 0.5 (\gamma_1 + \gamma_2) * 20/52$. Continuing this approach allowed the mean QALY and difference in QALY to be estimated over any chosen time horizon (3 years in the base-case, 4 years and 5 years in sensitivity analyses).

Joint uncertainty in mean costs and QALYs was quantified using bootstrapping. The results of the base case and sensitivity analyses were presented as mean estimates and as cost-effectiveness acceptability curves (CEACs). The incremental cost-effectiveness ratio was calculated (if appropriate) and compared to current decision making thresholds. These have been stated to be £20,000 – 30,000 in the United Kingdom.[11]

© 2020 Gohel M et al. *JAMA Surg.*

eFigure 1. Mortality Rate for Participants in the EVRA Trial

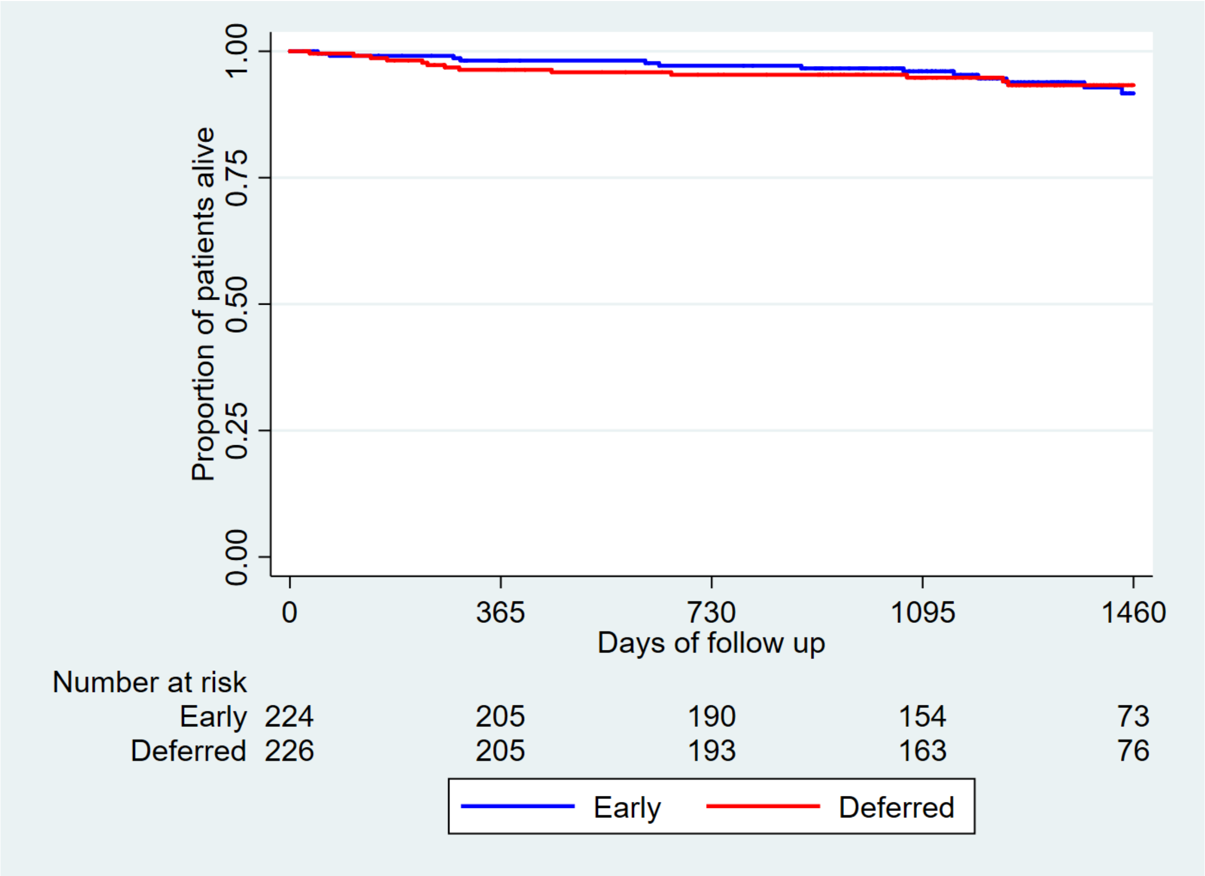

© 2020 Gohel M et al. *JAMA Surg.*

eFigure 2. Time to Ulcer Recurrence From Randomization

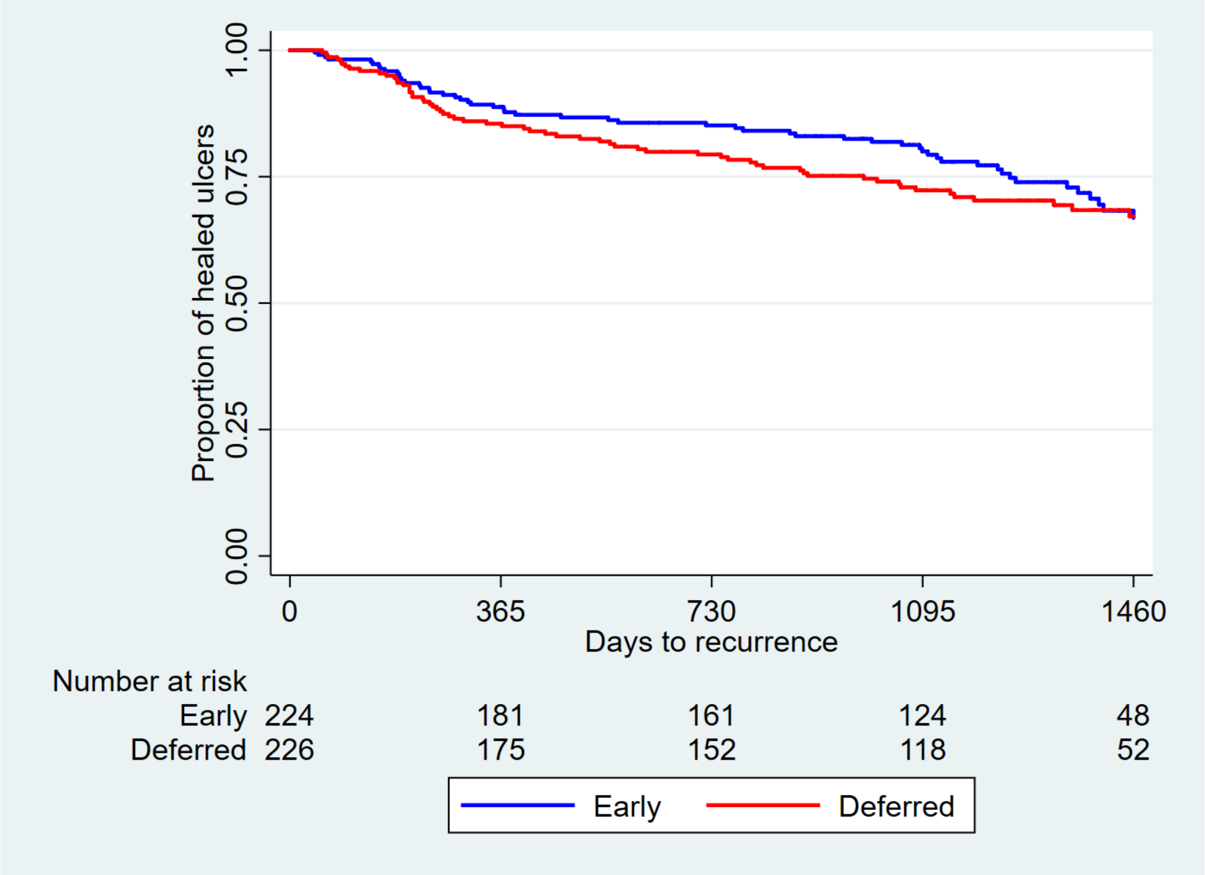

P=.34, Log-rank test

© 2020 Gohel M et al. *JAMA Surg.*

eFigure 3. Kaplan-Meier Curve for Time to Ulcer Healing for Rrecurrent Venous Leg Ulcers in the Early-Intervention Group and Deferred-Intervention Group

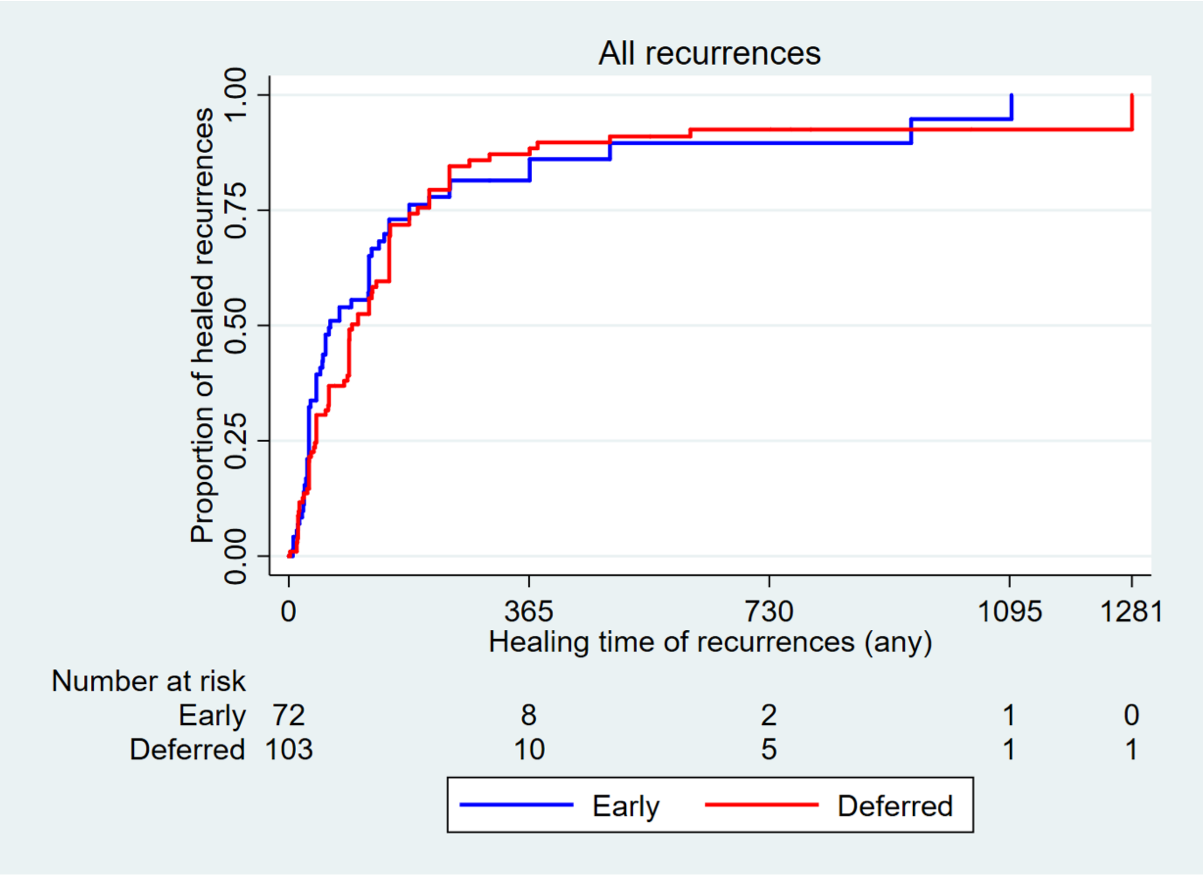

All episodes of recurrent ulceration included as separate events in analysis. Total of 175 recurrent ulcers in 120 participants

© 2020 Gohel M et al. *JAMA Surg*.

eFigure 4. Time to Ulcer Healing for Primary Ulcer

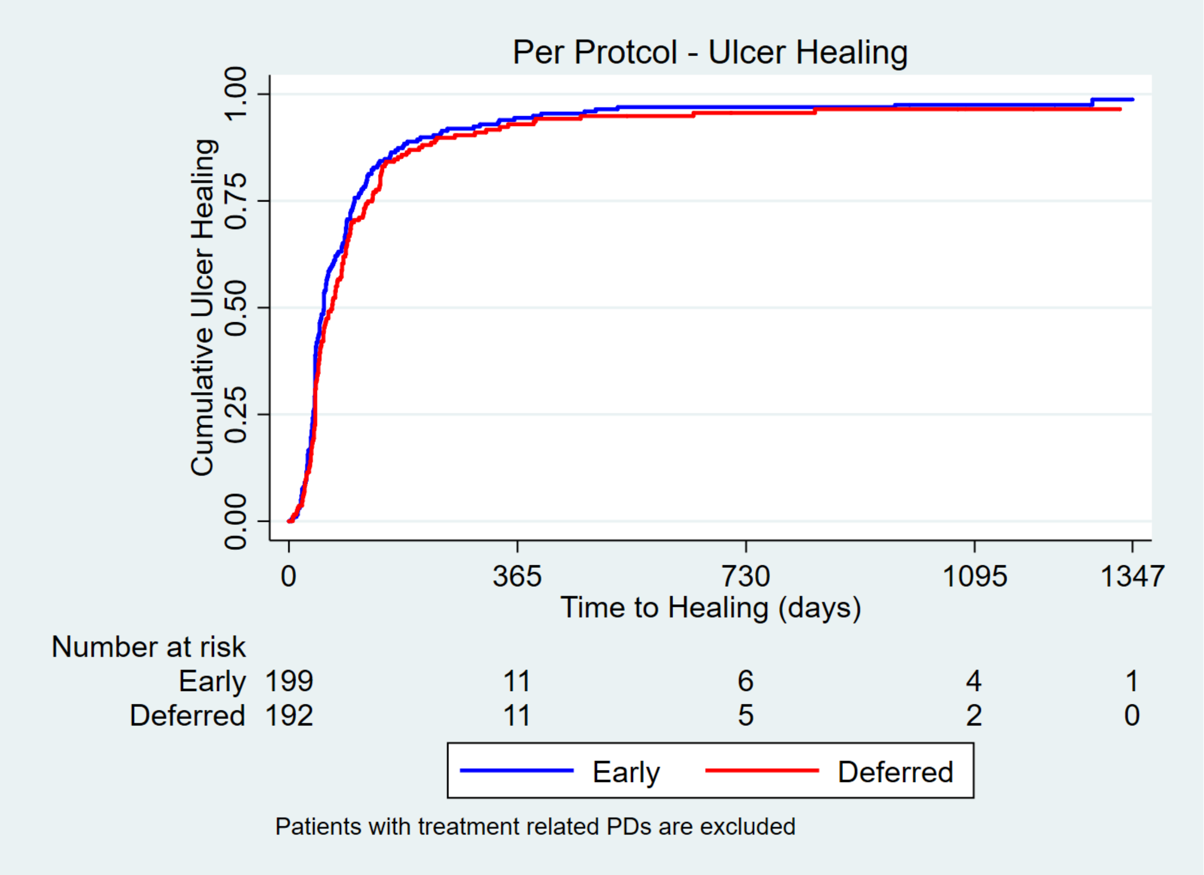

Hazard ratio for ulcer healing 1.15 (95% confidence interval 0.94 to 1.41)

© 2020 Gohel M et al. *JAMA Surg.*

eFigure 5. Time to First Ulcer Recurrence

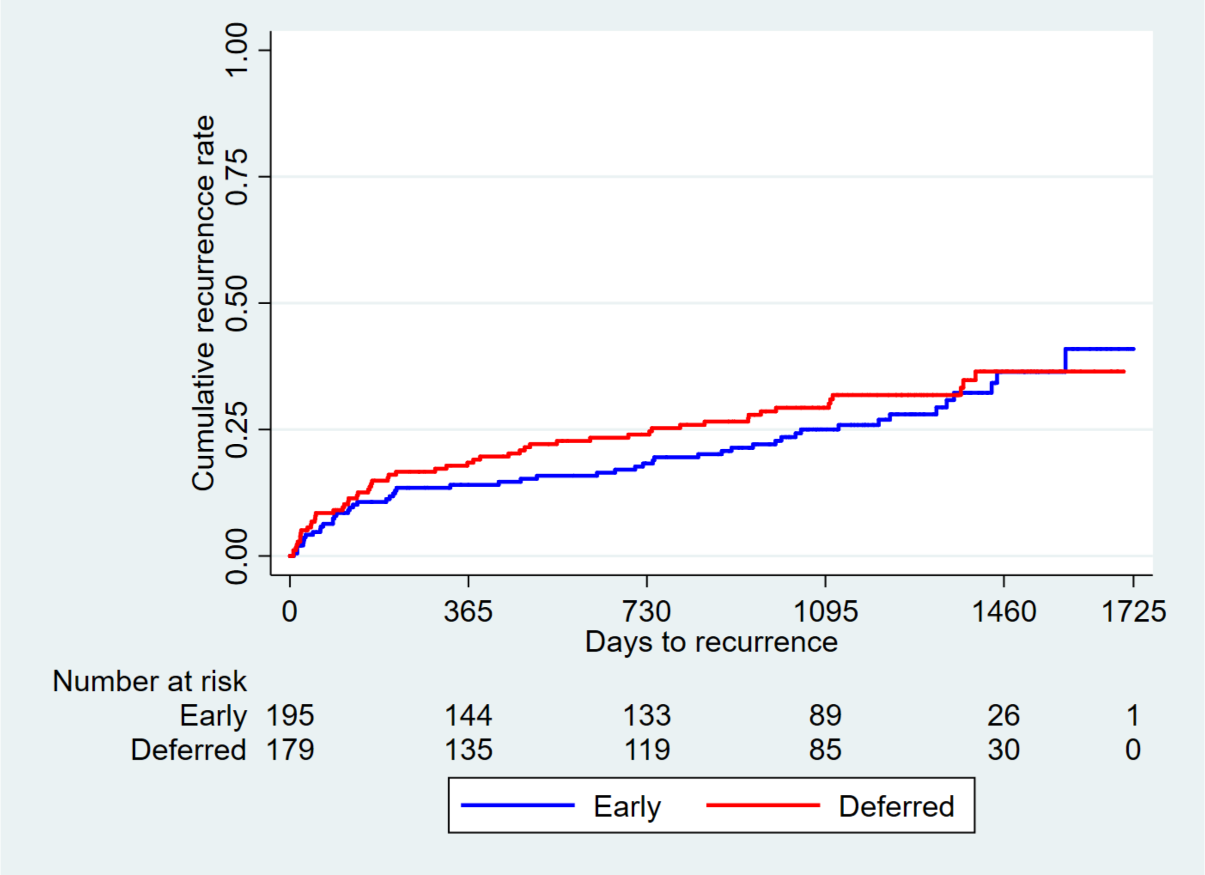

Hazard ratio for ulcer recurrence 0.86 (95% confidence interval 0.59 to 1.26)

© 2020 Gohel M et al. *JAMA Surg*.

eFigure 6. Cost-Effectiveness Acceptability Curve at 3 Years

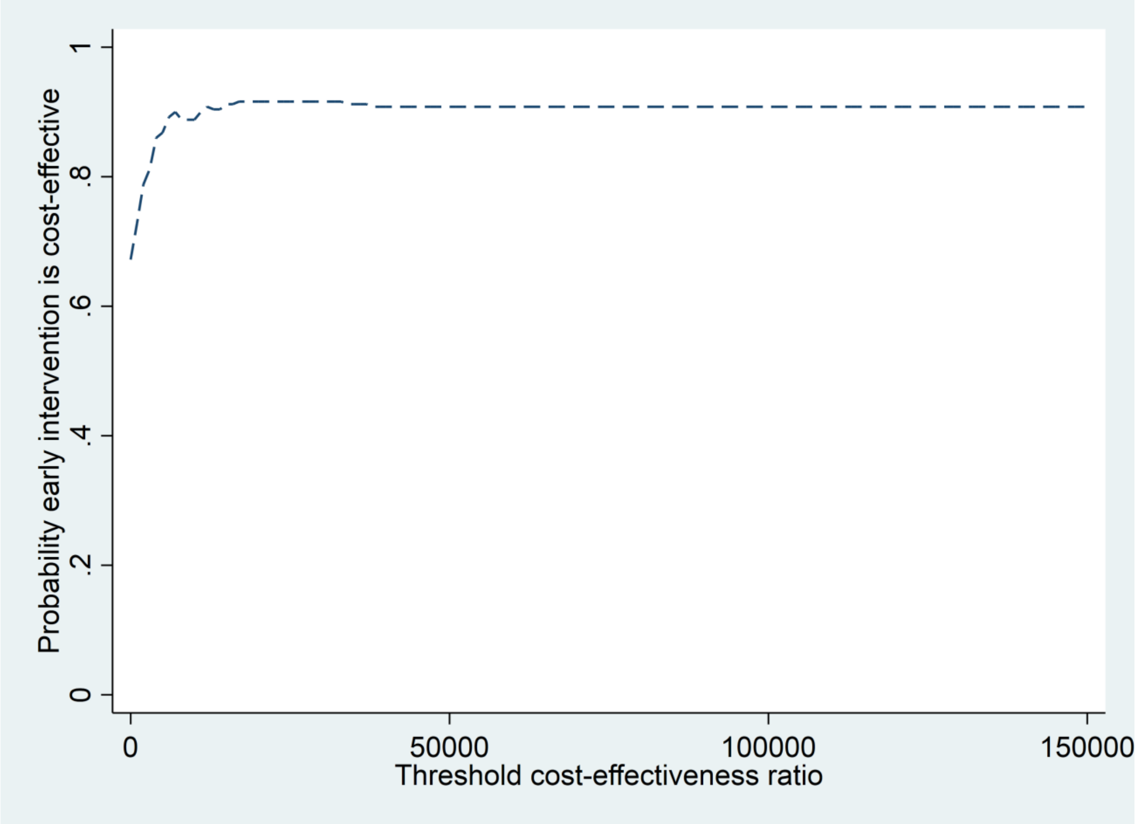

Cost-effectiveness threshold ratio in £ per QALY

© 2020 Gohel M et al. *JAMA Surg.*

eFigure 7. Sensitivity Analysis Using Monte Carlo Simulation

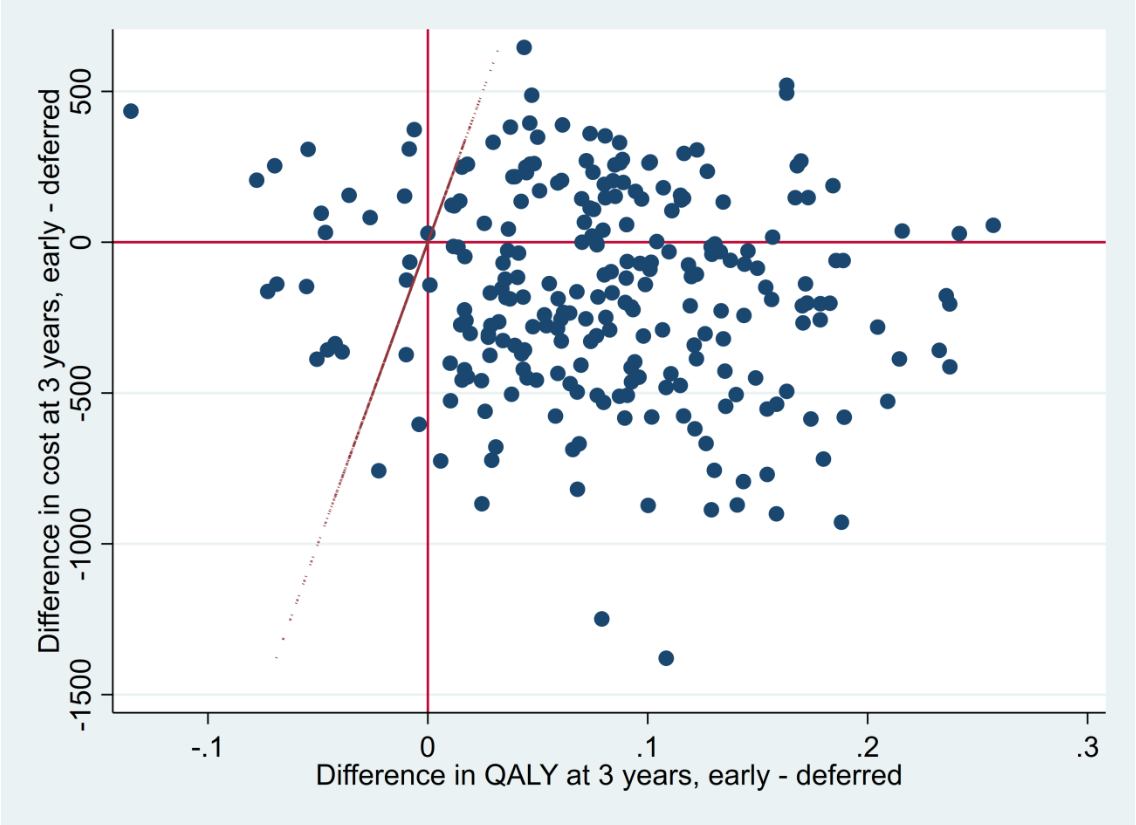

Dashed line shows the threshold for cost-effectiveness at a willingness to pay of £20,000 per quality-adjusted life year (QALY). Simulated points to the right of dashed line indicate cost-effectiveness at defined threshold

© 2020 Gohel M et al. *JAMA Surg.*

eTable 1. EVRA Study: Recruiting Centers

EVRA Site	Participants recruited
Gloucestershire Hospitals NHS Trust	124
Heart of England NHS Trust	51
Imperial College Healthcare NHS Trust	45
North Cumbria University Hospitals NHS Trust	32
North West London Hospitals NHS Trust	29
Cambridge University Hospitals NHS Foundation Trust	27
Plymouth Hospitals NHS Trust	23
Royal Bournemouth and Christchurch Hospitals NHS Foundation Trust	22
Taunton and Somerset NHS Foundation Trust	21
Worcestershire Acute Hospitals NHS Trust	20
University Hospitals Birmingham NHS Foundation Trust	9
The Dudley Group NHS Foundation Trust	8
Hull and East Yorkshire Hospitals NHS Trust	7
Frimley Park Hospital NHS Foundation Trust	6
Bradford Teaching Hospitals NHS Foundation Trust	6
Sheffield Teaching Hospitals NHS Foundation Trust	6
Salisbury NHS Foundation Trust	5
Leeds Teaching Hospitals NHS Trust	4
The Royal Wolverhampton Hospitals NHS Trust	3
York Hospitals NHS Foundation Trust	2

© 2020 Gohel M et al. *JAMA Surg.*

eTable 2. Summary of Secondary Outcome Measures and Quality of Life Tools Used in EVRA Study

**Details of outcome measure**	**Type of assessment**	**Range of scores**	**Comments**
Aberdeen Varicose Vein Questionnaire [12]	Patient reported disease specific quality of life	0 – 100 *	Higher scores indicate worse health related to varicose veins
EuroQol – 5 Dimension 5 Level (EQ-5D-5L) [10]	Patient reported generic quality of life	0 – 100 (health scale)	Consists of a health scale and health index, with higher scores indicating better health
Short-Form 36 (SF-36) [13]	Patient reported generic quality of life	0 – 100 (for each domain)	Eight scores covering different domains of health, with higher scores indicating better health

* previous studies have used 0.25SD as a clinically important difference [14]

© 2020 Gohel M et al. *JAMA Surg.*

eTable 3. Resource and Unit Costs Utilized for Health-Economic Analysis

**Resource**	**Unit Cost £**		**Source**
**Index procedure**			
**Staff procedure costs**			
EVLA	£5.49 / minute		Brittenden 2015, updated for inflation (Assumed same cost/minute for RFA)
UGFS	£4.67 / minute		Brittenden 2015, updated for inflation (Assumed same cost/minute for MOCA)
**Disposable kit or catheter prices**			
EVLA	£238.60		Angiodynamics list price (personal communication Caley Kitchen, Territory Manager – Vascular; 14/02/2018). List price catheter £200. Generator £22000- Assuming 2-year life, 600 procedures in total, cost of capital 3.5% per year. This gives an annuity cost per procedure of £38.60
RFA	£543		Medtronic list price. Personal communication (Harriet Ellis, vascular nurse specialist, 16/11/2017). Includes generator rental
MOCA	£375		Vascular Insights list price. Personal communication (Harriet Ellis, vascular nurse specialist, 16/11/2017)
**Other theatre consumables and anaesthetic**			
EVLA	£66		Brittenden 2015, adjusted for inflation
RFA	£66		Assumed same cost as EVLA
UGFS	£50		Brittenden 2015, adjusted for inflation
MOCA	£50		Assumed same cost as UGFS
**Other costs of vein ablations(pre-procedure and recovery)**			
EVLA	£72		Brittenden 2015, adjusted for inflation
RFA	£72		Assumed same cost as EVLA
UGFS	£42		Brittenden 2015, adjusted for inflation
MOCA	£42		Assumed same cost as UGFS

© 2020 Gohel M et al. *JAMA Surg.*

**Consumables ulcer healing**			
Urgo KTwo compression bandages	£7.84		NHS supply chain (Assumed changed 2 times per week until healing)
Ulcertec compression stockings	£27.10		NHS supply chain (Assumed two pairs changed every 3 months until healing)
Ulcer dressing			Assumed changed 2 times per week until healing
NA dressing	£11.20 for 40		NHS supply chain
Inadine™ 9.5x9.5cm	£15 for 25		NHS supply chain
Atrauman® dressing	£10.89 for 30		NHS supply chain
**Consumables after healing to prevent recurrence**			
Class 2 compression stocking	£31.27		NHS supply chain (Assumed changed every 3 months)
**Admissions to hospital for vein related procedures**			
Overnight stay without procedure	£376 / night		Reference cost 2017-18
Ablation procedure day case	£1191		Reference cost 2017-18
Angiogram and stent	£1265		Reference cost 2017-18
**Admissions to hospital (other than vein procedures)**			
Spinal surgery	£4231		Reference cost 2017-18
Shoulder replacement	£5675		Reference cost 2017-18
Ankle surgery	£2778		Reference cost 2017-18
Hip replacement	£6061		Reference cost 2017-18
Knee replacement	£5793		Reference cost 2017-18
Cataract	£924		Reference cost 2017-18
Hernia repair	£1858		Reference cost 2017-18
Pacemaker	£1631		Reference cost 2017-18
**Follow-up outpatient visit**			
Without procedure	£ 138 / visit		Reference cost 2017-18
Office-based sclerotherapy	£245 / visit		Brittenden 2015, updated for inflation
**Primary care**			

© 2020 Gohel M et al. *JAMA Surg.*

Visit to district nurse	£ 38/ visit		Reference cost 2017-18
District nurse home visit	£ 76/visit		Reference cost 2017-18 plus 15mins travel time
Visit to GP	£ 37/ visit		PSSRU 2018
GP home visit	£ 88/ visit		PSSRU 2018 (includes travel time)
**Other healthcare**			
Occupational therapist	£ 81/ visit		Reference costs 2017-18
Physiotherapist	£57 / visit		Reference costs 2017-18
Home carer visit (nursing care)	£76/ visit		Assume same as district nurse
Home help visit (personal care)	£29 / visit		Personal Social Services: Expenditure and Unit Costs, England, 2014-15 (updated for inflation)
**Medicines** British National Formulary			
Apixiban 2.5mg	£4.40 / day		5mg BD every day
Aspirin 75mg	£0.03 / day		75 mg OD every day
Clopidogrel 75mg	£0.06 / day		75 mg OD every day
Dalteparin 12,500units/ml	£20.32 / day		For average weight 96kg, 18000units / day (male)
	£14.12 / day		For average weight 80kg, 12500 units / day (female)
Warfarin	£0.04 / day		
Rivaroxaban 10mg	£3.60 / day		20mg OD
Clexane (Enoxaparin):	£11.02 / day		1.5mg/kg OD (male 96kg)
	£7.84 / day		1.5mg/kg OD (female 80kg)
Dabigatran 150mg	£1.70 / day		150mg BD

Resource use and unit costs (Curtis & Burns[4,5], Brittenden 2015[3], British National Formulary 2017[15], NHS reference costs[7], NHS Supply Chain[8])

© 2020 Gohel M et al. *JAMA Surg.*

eTable 4. Summary of Quality of Life Outcomes for Extended Follow-Up

		Baseline	6 weeks	6 months	12 months	Extended follow-up
**AVVQ**						
Early intervention		28.8 (11.3)	22.9 (11.8)	16.9 (10.9)	14.6 (9.6)	12.8 (10.2)
Deferred intervention		28.8 (10.7)	25.2 (11.0)	22.6 (12.2)	17.1 (12.1)	15.3 (12.8)
Difference†		0.0 (-2.2, 2.2)	-2.6 (-4.9, -0.3)	-5.0 (-7.5, -2.5)	-2.1 (-4.7, 0.5)	-2.4 (-4.9, 0.0)
**EQ-5D Health Score (Visual Analogue Scale)**						
Early intervention		70.2 (17.7)	72.7 (18.6)	74.1 (15.8)	74.8 (16.9)	70.3 (18.7)
Deferred intervention		70.1 (17.1)	71.1 (18.7)	71.4 (19.6)	73.7 (17.4)	68.4 (19.3)
Difference†		0.1 (-3.1, 3.4)	1.7 (-1.6, 5.1)	1.8 (-1.6, 5.2)	1.3 (-2.1, 4.8)	1.3 (-2.4, 4.9)
**EQ-5D Index Value‡**						
Early intervention		0.73 (0.2)	0.79 (0.2)	0.81 (0.2)	0.83 (0.2)	0.85 (0.3)
Deferred intervention		0.73 (0.2)	0.75 (0.2)	0.76 (0.2)	0.80 (0.2)	0.85 (0.3)
Difference†		-0.01 (-0.04, 0.03)	0.04 (0.00, 0.08)	0.04 (0.00, 0.08)	0.03 (-0.01, 0.07)	-0.02 (-0.08, 0.04)
**SF-36 Physical Function**						
Early intervention		37.3 (12.0)	39.1 (12.7)	39.1 (12.8)	39.4 (12.9)	37.8 (13.8)
Deferred intervention		37.5 (12.5)	37.4 (13.0)	37.4 (13.7)	38.7 (13.4)	37.6 (13.4)
Difference†		-1.0 (-3.1, 1.1)	1.0 (-1.2, 3.1)	0.7 (-1.5, 2.8)	0.3 (-1.9, 2.6)	0.0 (-2.4, 2.4)

© 2020 Gohel M et al. *JAMA Surg.*

**SF-36 Role-Physical**						
Early intervention		39.0 (12.2)	40.3 (12.5)	43.6 (12.6)	43.0 (12.7)	42.0 (12.7)
Deferred intervention		39.7 (12.1)	41.4 (12.7)	42.4 (12.7)	44.3 (12.9)	43.3 (12.5)
Difference†		-1.3 (-3.5, 0.9)	-1.7 (-4.0, 0.6)	0.4 (-2.0, 2.7)	-1.7 (-4.1, 0.7)	-1.5 (-4.1, 1.0)
**SF-36 Body Pain**						
Early intervention		41.3 (11.1)	46.6 (10.6)	48.2 (11.0)	49.3 (11.0)	47.3 (12.2)
Deferred intervention		41.6 (11.9)	44.3 (12.3)	45.9 (12.2)	47.8 (11.2)	48.1 (12.7)
Difference†		-0.5 (-2.6, 1.6)	2.2 (0.1, 4.4)	2.1 (-0.2, 4.3)	1.1 (-1.1, 3.3)	-1.2 (-3.6, 1.2)
**SF-36 General Health**						
Early intervention		45.8 (9.2)	45.7 (9.1)	44.9 (9.8)	45.3 (10.0)	44.1 (10.8)
Deferred intervention		46.0 (9.8)	45.6 (9.2)	44.5 (10.1)	45.1 (10.0)	44.4 (11.0)
Difference†		-0.3 (-2.0, 1.5)	-0.0 (-1.8, 1.8)	0.0 (-1.9, 1.8)	0.4 (-1.5, 2.3)	-0.5 (-2.5, 1.5)
**SF-36 Vitality**						
Early intervention		48.2 (10.2)	49.1 (10.0)	49.4 (9.5)	50.5 (9.4)	48.9 (10.8)
Deferred intervention		47.8 (10.6)	47.5 (11.3)	48.8 (10.8)	49.6 (9.8)	49.8 (10.3)
Difference†		0.1 (-1.7, 2.0)	1.4 (-0.5, 3.3)	0.0 (-1.9, 2.0)	0.9 (-1.0, 2.9)	-1.5 (-3.5, 0.6)
**SF-36 Social Functioning**						
Early intervention		42.6 (12.4)	44.9 (11.6)	47.0 (10.5)	47.4 (10.7)	46.7 (11.5)
Deferred intervention		42.4 (13.5)	44.0 (12.1)	44.7 (12.5)	47.3 (11.4)	46.2 (12.9)
Difference†		-0.1 (-2.3, 2.0)	0.6 (-1.6, 2.8)	1.5 (-0.8, 3.7)	-0.4 (-2.7, 2.0)	0.3 (-2.2, 2.8)

© 2020 Gohel M et al. *JAMA Surg.*

**SF-36 Role-Emotional**						
Early intervention		42.7 (13.8)	46.1 (12.8)	47.2 (13.2)	45.9 (13.0)	44.9 (13.6)
Deferred intervention		43.7 (13.6)	45.9 (13.3)	45.1 (13.2)	47.5 (12.2)	45.5 (13.4)
Difference†		-1.4 (-3.8, 1.0)	0.0 (-2.5, 2.5)	1.7 (-0.9, 4.2)	-1.7 (-4.3, 0.9)	-0.8 (-3.6, 1.9)
**SF-36 Mental Health**						
Early intervention		49.2 (10.3)	50.6 (10.4)	51.7 (9.7)	51.0 (9.3)	51.0 (10.2)
Deferred intervention		49.3 (10.7)	49.2 (10.8)	49.5 (10.4)	50.7 (10.1)	51.1 (10.0)
Difference†		-0.2 (-2.1, 1.7)	1.3 (-0.7, 3.2)	1.7 (-0.3, 3.7)	-0.2 (-2.2, 1.8)	-0.5 (-2.6, 1.6)
**SF-36 Physical Component Summary**						
Early intervention		38.5 (9.9)	40.4 (10.2)	41.5 (11.5)	42.1 (12.0)	40.5 (11.9)
Deferred intervention		38.8 (10.8)	39.6 (11.6)	40.4 (11.5)	41.8 (12.0)	41.0 (12.8)
Difference†		-0.8 (-2.8, 1.1)	0.3 (-1.7, 2.2)	0.3 (-1.7, 2.3)	0.3 (-1.7, 2.3)	-0.9 (-3.0, 1.3)
**SF-36 Mental Component Summary**						
Early intervention		49.2 (10.9)	51 (10.4)	52.2 (9.8)	51.6 (9.5)	51.3 (10.5)
Deferred intervention		49.4 (11.6)	50 (11.0)	50.2 (10.4)	52.0 (10.0)	51.5 (10.6)
Difference†		-0.3 (-2.2, 1.7)	0.9 (-1.1, 2.9)	1.5 (-0.5, 3.6)	-0.7 (-2.7, 1.4)	-0.6 (-2.8, 1.6)

Data presented as mean (SD). Widths of the confidence intervals have not been adjusted for multiplicity and should not be used for formal inference

† Difference between two arms estimated by mixed model adjusting for time, age, ulcer size and chronicity as fixed-effect, and study center and patient as random-effect; deferred intervention arm as reference; the 95% confidence intervals have not been adjusted for multiplicity

‡ EQ-5D index calculated using the value set for England

© 2020 Gohel M et al. *JAMA Surg.*

eTable 5. Use of Hospital and Community Health Services and Non-NHS Expenses During Extended Follow-Up Period

	EVRA (n=186)	Delayed (n=193)
**NHS resource use (number of episodes or visits)**		
Hospital admission	0.06 (0.34)	0.07 (0.31)
Outpatient visit	1.91 (4.03)	2.46 (5.67)
GP clinic visits	0.63 (4.80)	0.23 (0.94)
GP home visits	0 (0)	0.02 (0.23)
DN clinic visits	10.99(46.12)	8.74(24.16)
DN home visits	2.18 (14.09)	6.31(34.38)
Occupational therapy sessions	0 (0)	0.05 (0.59)
Physiotherapy sessions	0.04 (0.59)	0 (0)
**Other expenses for patients and family**		
Out of pocket expenses (Pounds)	£7.58(46.67)	£7.16(38.24)
Paid carer (days)	6.24 (53.82)	4.03 (52.63)
Unpaid carer (days)	20.17(120.08)	10.08 (75.82)
Days lost from paid work	0.73 (7.21)	2.07 (19.07)
Days lost from usual activities	7.97(57.57)	22.66 (129.98)
**Use of compression to prevent recurrence at extension follow up**		
None	80	67
Bandage	9	15
Stocking	104	113
**Use of medicines at extension follow-up**		
Antiplatelets		
Aspirin	33	34
Clopidogrel	6	10
Anticoagulants		
Warfarin	16	18
New oral anticoagulants	13	14
Other	2	2

Data presented as mean (SD).

© 2020 Gohel M et al. *JAMA Surg.*

eTable 6. Undiscounted Costs Per Patient at 3 Years in Each Treatment Group

**Resource**	**Delayed, £**	**EVRA. £**	**Difference, £**	**95% CI**	
Procedure	413	539	126	-186	439
Dressings & compression	607	507	-100	-265	65
Hospital and community services	2426	2255	-171	-1208	865
Medicine	28	26	-2	-38	35
Total	3493	3329	-164	-1295	967

© 2020 Gohel M et al. *JAMA Surg.*

eTable 7. Difference in Discounted Total Mean Costs and Discounted QALY at Different Time Horizons Estimated Using Mixed Model

**Time horizon**	**Difference in cost, £**	**95% CI**		**Difference in QALY (a)**	**95% CI**		**Difference in QALY (b)**	**95% CI**	
1 year	-67	-1011	788	0.053	0.022	0.084	0.041	0.013	0.069
3 year	-155	-1262	953	0.073	-0.057	0.204	0.024	-0.094	0.141
4 year	-132	-1322	1058	0.044	-0.139	0.226	-0.018	-0.183	0.147
5 year	-88	-1365	1188	0.051	-0.156	0.259	-0.029	-0.207	0.167

(a) QALY calculated using tariff recommended by the National Institute for Care Excellence[9]

(b) QALY calculated using alternative tariff [10]

The incremental cost-effectiveness ratio is not calculable, as EVRA provides greater QALY benefit at lower cost

© 2020 Gohel M et al. *JAMA Surg.*

eTable 8. Difference in Discounted Total Mean Costs and Discounted QALY at Different Time Horizons Estimated Using Mixed Model: per Protocol (a)

**Time horizon**	**Difference in cost, £**	**95% CI**		**Difference in QALY (b)**	**95% CI**		**Incremental cost-effectiveness ratio (c)**
1 year	224	-617	1066	0.057	0.023	0.090	£3929 /QALY
3 year	222	-764	1209	0.098	-0.042	0.237	£2265/QALY
4 year	270	-791	1331	0.093	-0.102	0.288	£2903/QALY
5 year	309	-829	1447	0.120	-0.102	0.342	£2575/QALY

(a) The per-protocol analysis excludes patients with treatment-related protocol deviations (33 deferred intervention, 24 early intervention)

(b) QALY calculated using tariff recommended by the National Institute for Care Excellence (van Hout et al. 2012)

(c) The incremental cost- effectiveness ratio (ICER) is calculated as the difference in mean cost divided by the difference in mean QALY between the treatments, when the intervention costs more than the comparator and provides greater health benefit.

© 2020 Gohel M et al. *JAMA Surg.*

eReferences

1. Guide to the Methods of Technology Appraisal 2013. London; 2013.

2. Husereau D, Drummond M, Petrou S, et al. Consolidated Health Economic Evaluation Reporting Standards (CHEERS)--explanation and elaboration: a report of the ISPOR Health Economic Evaluation Publication Guidelines Good Reporting Practices Task Force. *Value Health* 2013; **16**(2): 231-50.

3. Brittenden J, Cotton SC, Elders A, et al. Clinical effectiveness and cost-effectiveness of foam sclerotherapy, endovenous laser ablation and surgery for varicose veins: results from the Comparison of LAser, Surgery and foam Sclerotherapy (CLASS) randomised controlled trial. *Health Technol Assess* 2015; **19**(27): 1-342.

4. Curtis LA, Burns A. Unit Costs of Health & Social Care 2015: PSSRU: Personal Social Services Research Unit (2015). 2015.

5. Curtis LA, Burns A. Unit costs of health and social care. doi:10.22024/UniKent/01.02.70995; 2018.

6. Improvement N. National Schedule of Reference Costs 2017/18; 2018.

7. NHS Improvement. National Schedule of Reference Costs 2017/18; 2018.

8. NHS Supply Chain. Advanced Wound Care. Dressings Price List 2017. Available at: https://www.supplychain.nhs.uk/product-information/contract-launch-brief/advanced-wound-care/. ; 2017.

9. van Hout B, Janssen MF, Feng YS, et al. Interim scoring for the EQ-5D-5L: mapping the EQ-5D-5L to EQ-5D-3L value sets. *Value Health* 2012; **15**(5): 708-15.

10. Devlin NJ, Shah KK, Feng Y, Mulhern B, van Hout B. Valuing health-related quality of life: An EQ-5D-5L value set for England. *Health Econ* 2018; **27**(1): 7-22.

11. Neumann PJ, Cohen JT, Weinstein MC. Updating cost-effectiveness--the curious resilience of the $50,000-per-QALY threshold. *N Engl J Med* 2014; **371**(9): 796-7.

© 2020 Gohel M et al. *JAMA Surg.*

12. Garratt AM, Macdonald LM, Ruta DA, Russell IT, Buckingham JK, Krukowski ZH. Towards measurement of outcome for patients with varicose veins. *Qual Health Care* 1993; **2**(1): 5-10.

13. Garratt AM, Ruta DA, Abdalla MI, Russell IT. Responsiveness of the SF-36 and a condition-specific measure of health for patients with varicose veins. *Qual Life Res* 1996; **5**(2): 223-34.

14. Brittenden J, Cotton SC, Elders A, et al. A randomized trial comparing treatments for varicose veins. *N Engl J Med* 2014; **371**(13): 1218-27.

15. British National Formulary. BNF 73; 2017.

© 2020 Gohel M et al. *JAMA Surg.*
