# Supplementary material for: Long-term Clinical and Cost-effectiveness of Early Endovenous Ablation in Venous Ulceration: A Randomized Clinical Trial
Source: JAMA Surg. 2020 Sep 23;155(12):1113–21. doi: 10.1001/jamasurg.2020.3845 (PMC7512122; doi:10.1001/jamasurg.2020.3845)
Supplement: Supplement 3. — Data Sharing Statement [file jamasurg-e203845-s003.pdf]

Data Sharing Statement

Gohel. Long-Term Clinical and Cost-Effectiveness of Early Endovenous Ablation in Venous Ulceration. *JAMA Surg*. Published September 23, 2020. 10.1001/jamasurg.2020.3845

Data
Data available: Yes
Data types: Deidentified participant data
How to access data: Imperial College, London
a.h.davies@imperial.ac.uk
When available: With publication

Supporting Documents
Document types: None

Additional Information
Who can access the data: Researchers request data In line with NIHR data release and Sponsor Imperial College
Types of analyses: Health care Medical
Mechanisms of data availability: Signed request and with approval of Imperial College, London the Sponsor of the study
Any additional restrictions: None
